# Analysis of the transcriptome of *Panax notoginseng *root uncovers putative triterpene saponin-biosynthetic genes and genetic markers

**DOI:** 10.1186/1471-2164-12-S5-S5

**Published:** 2011-12-23

**Authors:** Hongmei Luo, Chao Sun, Yongzhen Sun, Qiong Wu, Ying Li, Jingyuan Song, Yunyun Niu, Xianglin Cheng, Hongxi Xu, Chuyuan Li, Juyan Liu, André Steinmetz, Shilin Chen

**Affiliations:** 1 The Key Laboratory of Bioactive Substances and Resources Utilization of Chinese Herbal Medicine, Ministry of Education, Institute of Medicinal Plant Development, Chinese Academy of Medical Sciences & Peking Union Medical College, Beijing 100193, P. R. China; 2 School of Pharmacy, Guilin Medical University, Guilin 541004, China; 3 China National Center for Biotechnology Development, Beijing 100036, China; 4 Shanghai University of Traditional Chinese Medicine, Shanghai, China; 5 Guanzhou Pharmaceutical Holding Limited, Guangzhou, China; 6 Centre de Recherche Public-Santé, Luxembourg, L-1526 Luxembourg, Belgium

## Abstract

**Background:**

*Panax notoginseng *(Burk) F.H. Chen is important medicinal plant of the *Araliacease *family. Triterpene saponins are the bioactive constituents in *P. notoginseng*. However, available genomic information regarding this plant is limited. Moreover, details of triterpene saponin biosynthesis in the *Panax *species are largely unknown.

**Results:**

Using the 454 pyrosequencing technology, a one-quarter GS FLX titanium run resulted in 188,185 reads with an average length of 410 bases for *P. notoginseng *root. These reads were processed and assembled by 454 GS *De Novo *Assembler software into 30,852 unique sequences. A total of 70.2% of unique sequences were annotated by Basic Local Alignment Search Tool (BLAST) similarity searches against public sequence databases. The Kyoto Encyclopedia of Genes and Genomes (KEGG) assignment discovered 41 unique sequences representing 11 genes involved in triterpene saponin backbone biosynthesis in the 454-EST dataset. In particular, the transcript encoding dammarenediol synthase (DS), which is the first committed enzyme in the biosynthetic pathway of major triterpene saponins, is highly expressed in the root of four-year-old *P. notoginseng*. It is worth emphasizing that the candidate cytochrome P450 (Pn02132 and Pn00158) and UDP-glycosyltransferase (Pn00082) gene most likely to be involved in hydroxylation or glycosylation of aglycones for triterpene saponin biosynthesis were discovered from 174 cytochrome P450s and 242 glycosyltransferases by phylogenetic analysis, respectively. Putative transcription factors were detected in 906 unique sequences, including Myb, homeobox, WRKY, basic helix-loop-helix (bHLH), and other family proteins. Additionally, a total of 2,772 simple sequence repeat (SSR) were identified from 2,361 unique sequences, of which, di-nucleotide motifs were the most abundant motif.

**Conclusion:**

This study is the first to present a large-scale EST dataset for *P. notoginseng *root acquired by next-generation sequencing (NGS) technology. The candidate genes involved in triterpene saponin biosynthesis, including the putative CYP450s and UGTs, were obtained in this study. Additionally, the identification of SSRs provided plenty of genetic makers for molecular breeding and genetics applications in this species. These data will provide information on gene discovery, transcriptional regulation and marker-assisted selection for *P. notoginseng*. The dataset establishes an important foundation for the study with the purpose of ensuring adequate drug resources for this species.

## Background

*Panax notoginseng *(Burk) F.H. Chen is highly valued medicinal plant of the *Araliaceae *family [1]. *P. notoginseng *is distributed in the southwestern region of China, Burma and Nepal [2]. Presently, this species can only be found in cultivated forms [3]. In China, *P. notoginseng *is cultiviated commercially in Wenshan County, Yunnan province [4]. The roots of this plant, called notoginseng or sanchi, are commonly used as a hemostatic agent as well as a tonic to promote quality of life. In addition, the herb of sanqi possesses the bioactivities of antihypertensive, antithrombotic, anti-atherosclerotic and neuroprotective actions [3]. The ingredients detected in *P. notoginseng *include triterpene saponins, non-protein amino acids, polyacetylenes, phytosterols, flavonoids, and polysaccharides, many of which have pharmacological activities and are useful in the treatment of some diseases [2]. Among these compounds, triterpene saponins, a group of ginsenosides, are considered to be the principal bioactive components responsible for the pharmacological features [5-7]. Approximately 60 triterpene saponins have been isolated from *P. notoginseng *including ginsenosides, notoginsenosides, and gypenosides [2]. The major ginsenosides are the dammarane glycosides, and the ginsenoside Rg1, Rb1, Rd, and notoginsenoside R1 are considered as the major constituents found in the *P. notoginseng *root [8]. All the dammarane saponins have been classified as two groups: the protopanaxadiols group and the protopanaxatriols group [2]. The oleanane-type saponin, Ro, which exists in Asian ginseng (*Panax ginseng*) and American ginseng (*Panax quinquefolius*), have not been found in *P. notoginseng *based on the evidence from phytochemical studies [2].

**Figure 1:**
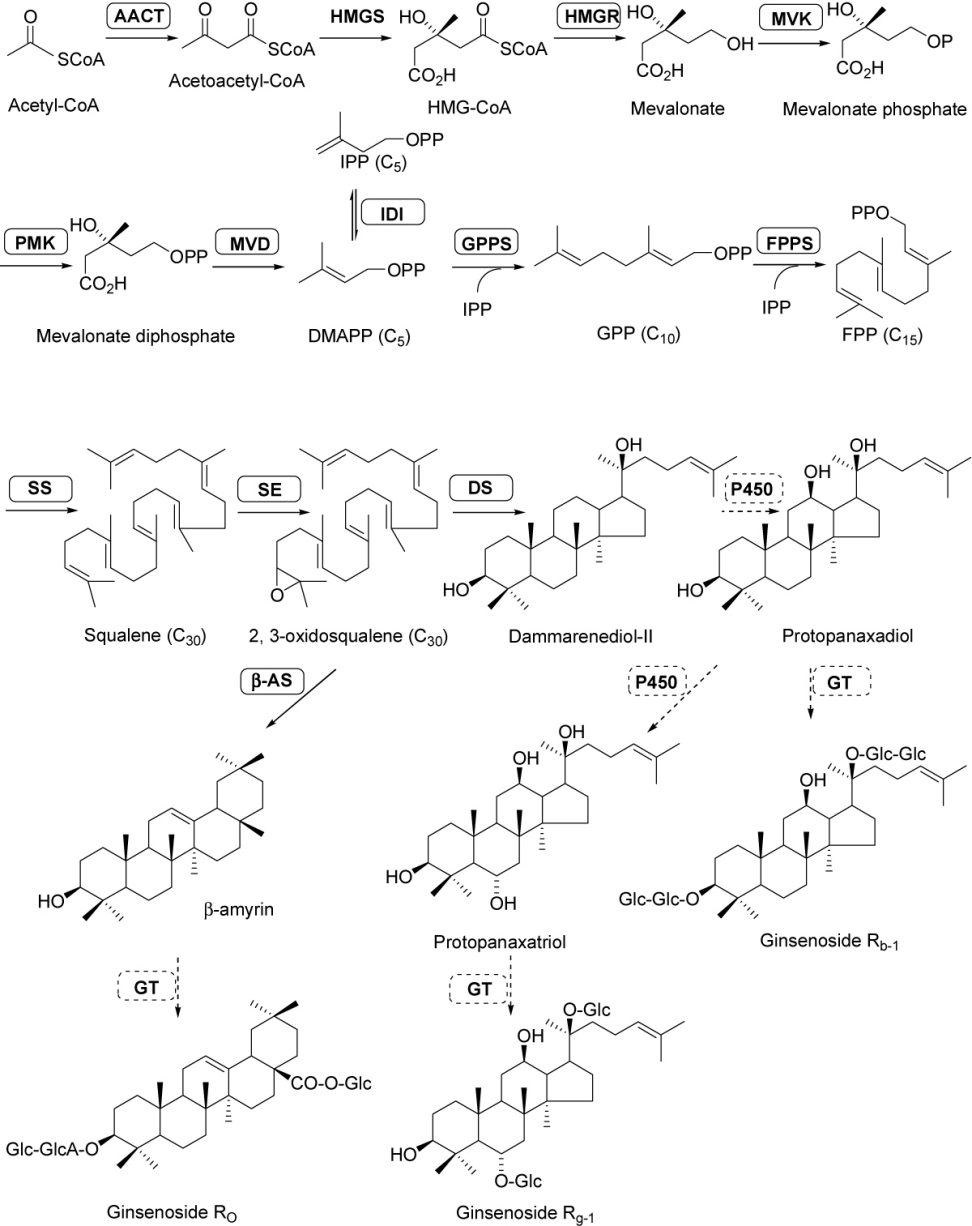
**Putative pathway for triterpene saponin biosynthesis in *P. notoginseng***. Enzymes found in this study are boxed. Abbreviations: AACT, acetyl-CoA acetyltransferase; β-AS, β-amyrin synthase; DMAPP, dimethylallyl diphosphate;DS, dammarenediol-II synthase; FPP, farnesyl diphosphate; FPPS, farnesyl diphosphate synthase; Glc, glucose; GPP, geranyl pyrophosphate; GGPP, geranylgeranyl diphosphate; GGPPS, geranylgeranyl pyrophosphate synthase; GT, glycosyltransferase; HMG-CoA, 3-hydroxy-3-methylglutaryl coenzyme A; HMGR, HMG-CoA reductase; HMGS, HMG-CoA synthase; IPP, isopentenyl diphosphate; IPPI, IPP isomerase; MVD, mevalonate diphosphate decarboxylase; MVK, mevalonate kinase; P450, cytochrome P450; PMK, phosphomevalonate kinase; SE, squalene epoxidase; SS, squalene synthase.

Triterpene saponins are synthesized via the mevalonic acid (MVA) pathway [9], which is ubiquitous in plants and provides the precursor 2,3-oxidosqualene for terpenoid biosynthesis. The cyclization of 2,3-oxidosqalene by oxidosqualene cyclase (OSC) combined with the following modifications on the triterpene skeletons including hydroxylation and glycosidation leads to the production of various ginsenosides (Figure 1). The OSC genes including *dammarenediol synthase *(*DS*), *β-amyrin *(*β-AS*), *lupeol synthase *(*LS*) and *cycloartenol synthase *(*CAS*) have been isolated in plants [10-13]. The characterization of DS, the first key enzyme committed in the biosynthesis of dammarane-type saponins, was profoundly advanced the studies on triterpene saponin biosynthesis in *P. ginseng *[10,14]. However, little is known about the molecular mechanism of the biosynthetic pathway downstream of cyclization involved in ginsenoside biosynthesis (Figure 1). Some specific cytochrome P450-dependent monoxygenases (CYP450s) and UDP-glycosyltransferases (UGTs), which might be existed in *Panax *plants, are proposed to catalyze the conversion of dammarenediol-II or β-amyrin to various ginsenosides and the modification on ginsenosides. In our previous study, one candidate *CYP450 *and four candidate *UGTs *most likely to be involved in ginsenoside biosynthesis have been selected from *P. quinquefolius *[15]. These candidate genes were screened from the 454-EST dataset of *P. quinquefolius *root based on the analysis of tissue-specific expression pattern and methyl jasmonate (MeJA) induction [15].

Despite its pharmacological importance, the transcriptomic and genomic data of *P. notoginseng *are very limited and only 95 ESTs are available in the National Center for Biotechnology Information (NCBI) database. The limited transcriptomic data hinder the study of triterpene saponin biosynthetic mechanisms in *P. notoginseng*. Expressed sequence tag (EST) analysis is a useful tool for the purposes of gene discovery especially in non-model plants for which no reference genome sequences are available [16]. ESTs represent the expressed portion of a genome [17,18] and can be used to characterize patterns of gene expression in special tissues [19]. The discovery and prediction of genes involved in triterpene saponin and other secondary metabolite biosynthesis was performed based on EST analysis [15,20,21]. The triterpene carboxylic acid glucosyltransferase was characterized by mining ESTs from the developing seeds of *Saponaria vaccaria *[22]. The licorice-amyrin 11-oxidase gene, which plays a key role in the biosynthesis of the triterpene sweetener glycyrrhizin, was identified from the ESTs generated from the stolons of *Glycyrrhiza uralensis *[23]. In addition, ESTs are a rich source of gene-derived molecular markers (e.g. simple sequence repeat, SSR) which will be used for germplasm breeding or physical mapping [24]. The next-generation sequencing (NGS) technologies improve sequencing depth and render large-scale EST projects more feasible [25-27].

Herein we present the results of the study designed to characterize the transcriptome of *P. notoginseng *root using NGS technology based on 454 GS FLX Titanium platform. Our ultimate goal is to discover the candidate genes that encode enzymes in the triterpene saponin biosynthetic pathway and provide an overview of transcriptome, as well as produce molecular markers of EST-SSRs for facilitation the marker-assisted breeding of this species.

## Results and discussion

### Transcriptome sequencing and sequence assembly

**Figure 2:**
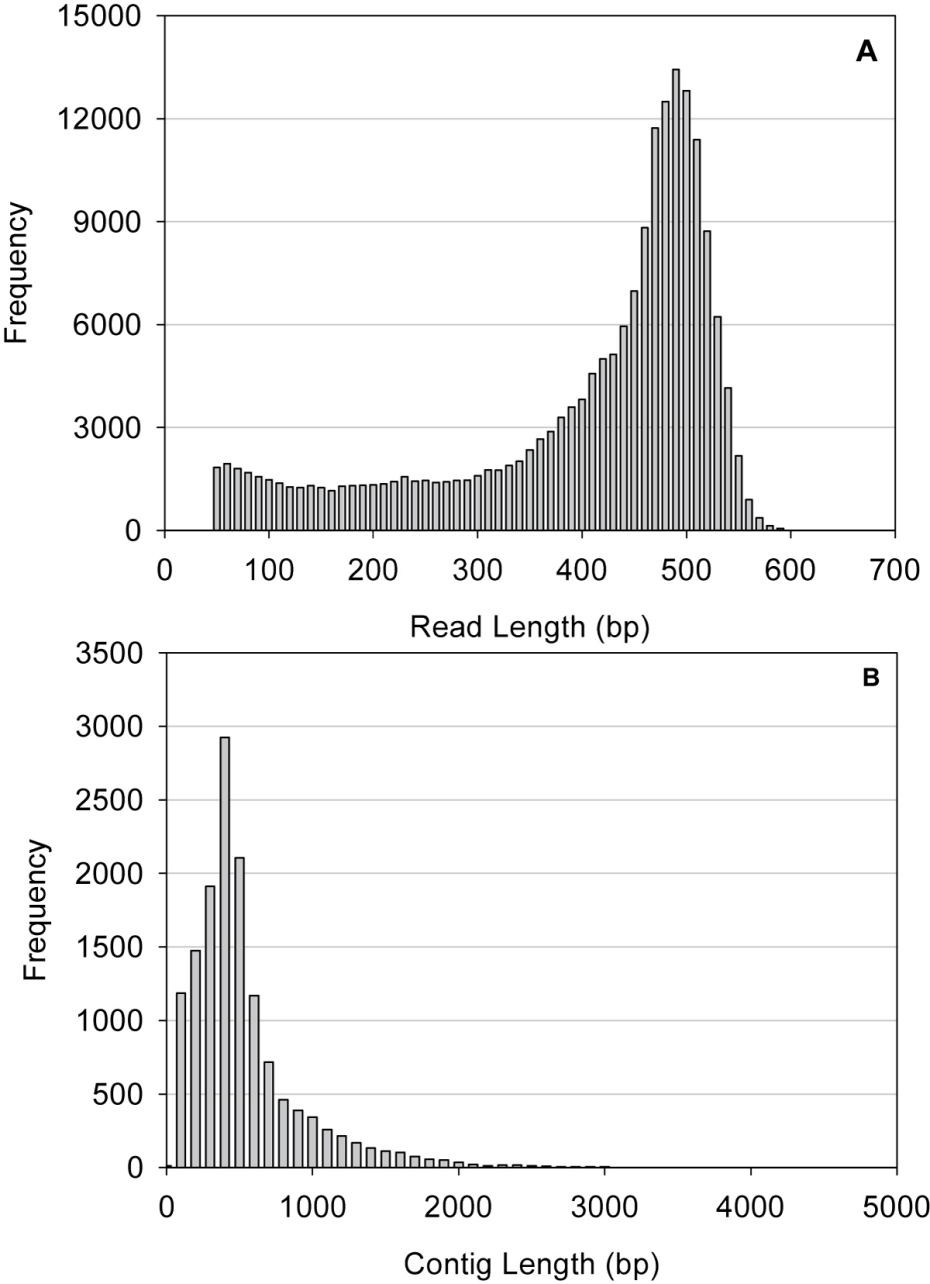
**Length distributions of reads and contigs of *P. notoginseng***. A. Size distribution of 454 sequencing reads of *P. notoginseng *after removal of adaptor sequences. B. Length distribution of contigs (assembled sequences) in *P. notoginseng *454-EST dataset.

A one-quarter 454 GS FLX Titanium run representing the cDNA library of 4-year old *P. notoginseng *root produced 188,185 reads with an average length of 410 bp (Table 1). After trimming adaptor sequences and removing those reads shorter than 50 bp, a total of 184,785 reads were assembled into 30,852 unique sequences including 14,005 contigs and 16,847 singletons (Table 1). The lengths of contigs ranged from 95 to 10,423 bp with average size of 581 bp (Table 1). The size distributions for these reads and contigs are shown in Figure 2. Singletons, which represent unique transcripts expressed at low levels in the samples and with only one read, exhibited an average length of 343 bp in *P. notoginseng *454-EST dataset (Table 1). The length of these unique sequences was sufficient to enable annotations with high accuracy [28]. An overview of 454 sequencing and assembly for *P. notoginseng *was summarized in Table 1.

**Table 1:** Summary of 454 sequencing and assembly for *P. notoginseng*

	No. of sequences	No. of bases
HQ reads	188,185	77,079,504
Average HQ read length	410 ± 138 bp	
Reads used in assembly	184,785	75,152,969

Reads assembled as contigs	164,855	65,067,207
Number of contigs	14,005	8,131,261
Average length of contigs	581 ± 404 bp	
Range of contig lengths	95 - 10,423 bp	
Contigs above 200 bp	12,809	7,956,026

Number of singletons	16,847	5,775,116
Average length of singletons	343 ± 166 bp	
Range of singleton lengths	50 - 728 bp	
Singletons above 200 bp	12,365	5,310,178

Number of unique sequences^a^	30,852	
Unique sequences above 200 bases	25,174	

^a ^The total number of contigs and singletons.

### Unique sequence annotation and highly expressed transcript analyses

The annotation for *P. notoginseng *unique sequences was based on sequence similarity searches against public databases. These databases included SwissProt, KEGG, The Arabidopsis Information Resource (TAIR), NCBI non-redundant protein (Nr), and NCBI non-redundant nucleotide (Nt) database. The number and percentage of the annotated unique sequences were summarized in Additional file 1. In total, 21,672 (70.2%) *P. notoginseng *unique sequences were annotated and the remaining (29.8%) unique sequences had no match to any sequences in the public databases (Table 1). The annotation rate for *P. notoginseng *unique sequences is similar to that of *P. quinquefolius *root [15] and *P. ginseng *root [29] transcriptomes. The comparisons of the 30,852 *P. notoginseng *unique sequences with all the *P. quinquefolius *[15] and *P. ginseng *[29] unique sequences derived from 454 sequencing using BLAST search (*E*-value <= 1e-10) were performed. The result of the comparison between *P. notoginseng *and *P. quinquefolius *[15] unique sequences showed that 19,226 *P. notoginseng *unique sequences had sequence similarity to *P. quinquefolius *unique sequences, and the remained 11,626 unique sequences were the *P. notoginseng *special transcripts. Similarly, a total of 19,479 *P. notoginseng *unique sequences had sequence similarity to *P. ginseng *unique sequences [29], and the rest 11,373 unique sequences were the *P. notoginseng *special transcripts.

The abundance of a transcript in a cDNA library from specific tissues/organs generally corresponds to its expression level in the original biological sample, which can indicate the ongoing biological processes [30]. The top 20 most frequent unique transcripts in the cDNA library of the 4-year old *P. notoginseng *root were shown in Table 2. The most abundant *P. notoginseng *transcript (with 3,358 reads) had no hit to any sequences of the public databases. The transcripts encoding enzymes associated with sugar and energy metabolism were highly expressed in this cDNA library, including 1,4-alpha-glucan-branching enzyme protein, sucrose synthase isoform, and formate dehydrogenase (Table 2). The abundance of these transcripts was consistent with the fact that starch is the major constituent in the *P. notoginseng *root. It is noteworthy that three transcripts encoding the key enzymes of Acetyl-CoA acetyltransferase (AACT), squalene epoxidase (SE) and dammarenediol synthase (DS) were also abundant in *P. notoginseng *root (Table 2). These enzymes play important roles in triterpene saponin biosynthesis, particularly for DS, which is the first committed enzyme in the biosynthetic pathway of major triterpene saponins. In comparison, *DS *was more abundant in *P. notoginseng *root than in *P. quinquefolius *[15] and *P. ginseng *root [29]. The high expression levels of these transcripts encoding DS indicated that triterpene saponin biosynthesis is active in the growth stage of 4-year old of this species. Therefore, the genes (e.g. some specific CYP450s and UGTs) involved in the downstream of triterpene saponin biosynthetic pathway are likely to be abundant in this 454-EST dataset. In addition, several transcripts encoding heat shock proteins, which have the functions in the abiotic stress response in plants [31], were also expressed highly in *P. notoginseng *root (Table 2).

**Table 2:** The 20 most abundant transcripts in *P. notoginseng *root

Rank	Contig	Gene ID	Gene name	No. of reads
1	contig14005	No hit		3,358
2	contig13784	sp|Q9SVG4|	Reticuline oxidase -like protein	3,043
3	contig13829	gb|AAX40471.1|	*P. ginseng*-specific abundant protein 3	2,114
4	contig13823	gb|AY829463.1|	*P. ginseng*-specific abundant protein 3	1,949
5	contig00208	sp|P30924|	1,4-alpha-glucan- branching enzyme	1,748
6	contig00313	dbj|BAF98277.1|	Acetyl-CoA acetyltransferase	1,301
7	contig00270	gb|EEF30081.1|	Heat shock protein, putative	1,129
8	contig00110	dbj|BAB68539.1|	(S)-reticuline oxidase-like protein	1,048
9	contig13785	dbj|BAF33291.1|	*P. ginseng *dammarenediol-II synthase	1,018
10	contig13417	gb|AAO38031.1|	Delta12-fatty acid acetylenase	1,009
11	contig00111	gb|ACJ24907.2|	*P. ginseng *squalene epoxidase	865
12	contig00323	No hit		778
13	contig13999	gb|DQ384527.1|	*P. ginseng *clone PG6L-4	747
14	contig00310	gb|EEF49052.1|	Heat shock protein, putative	742
15	contig00282	gb|AAR17080.1|	Heat shock protein 70-3	711
16	contig00123	sp|P49035|	Sucrose synthase isoform 1	616
17	contig13552	gb|ABB29477.1|	Tonoplast intrinsic protein	538
18	contig00095	sp|Q07511|	Formate dehydrogenase	537
19	contig00665	ref|YP_588403.1|	Hypothetical protein	527
20	contig13555	gb|EEF34649.1|	Heat shock protein	511

### GO analysis and KEGG assignment

**Figure 3:**
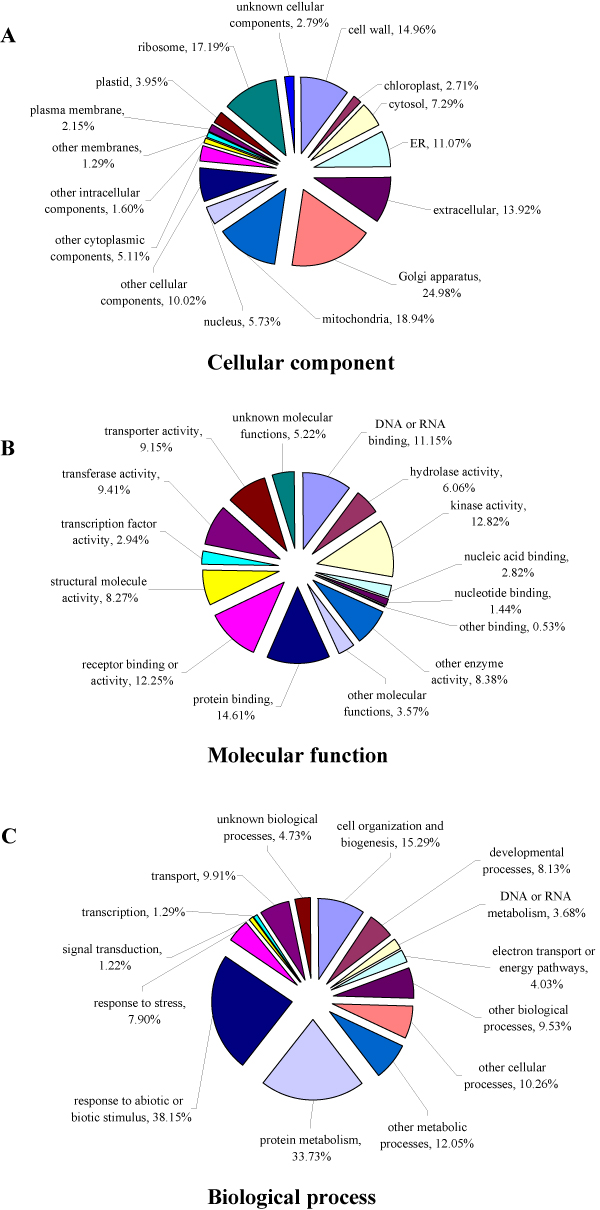
**GO analysis of *P. notoginseng *unique sequences based on cellular component (Figure 3A), molecular function (Figure 3B), and biological process (Figure 3C)**.

The GO annotation describes gene products according to their associated molecular functions, cellular components, and biological processes, illustrating the broad overview of the groups of genes cataloged in transcriptome [32]. A total of 18,689 *P. notoginseng *unique sequences were assigned with GO terms based on sequence similarity to proteins in TAIR database (Figure 3). In cellular component group, the unique sequences related to the Golgi apparatus, mitochondria, ribosome, and cell wall were well-represented categories (Figure 3A). The transcripts belonging to the major subgroups of molecular function category included protein binding, kinase activity, and receptor binding or activity (Figure 3B). The best-represented groups of biological processes were response to abiotic or biotic stimuli, protein metabolism, cell organization and biogenesis (Figure 3C). These GO annotations provide a comprehensive information on transcript functions of *P. notoginseng*.

The KEGG assignments provide an alternative functional annotation of genes associated biochemical pathways with their corresponding enzyme commission (EC) numbers [33]. 16,300 *P. notoginseng *unique sequences were assigned to KEGG annotations based on sequence similarity searches against the KEGG database. A total of 3,862 unique sequences among the 16,300 annotated sequences were assigned to biochemical pathways (Additional file 2). The remaining (12,438) unique sequences were not assigned to any known biochemical pathway (Additional file 2). In detail, a total of 1,349 unique sequences were assigned to the metabolic pathways, including the well-represented pathways of amino acid metabolism, carbohydrate metabolism, lipid metabolism, energy metabolism, and the biosynthesis of secondary metabolites (Additional file 2). The number of unique sequences involved in the biosynthesis of secondary metabolites was shown in Additional file 3. In particular, the transcripts encoding most of all the known enzymes (except the HMG-CoA synthase, HMGS) involved in the triterpene saponin biosynthesis were discovered in this 454-EST dataset, including acetyl-CoA acetyltransferase (AACT), HMG-CoA reductase (HMGR), mevalonate kinase (MVK), phosphomevalonate kinase (PMK), mevalonate diphosphate decarboxylase (MVD), IPP isomerase (IPPI), geranylgeranyl pyrophosphate synthase (GGPPS), farnesyl diphosphate synthase (FPPS), squalene synthase (SS), squalene epoxidase (SE), and dammarenediol-II synthase (DS) (Figure 1 andTable 3). Pathways related to genetic information processing, protein families, and cellular processes were also well represented by *P. notoginseng *unique sequences (Additional file 2).

**Table 3:** Genes involved in triterpene saponin biosynthesis in *P. notoginseng *454-EST dataset

Gene name	EC	Unique sequences
Acetyl-CoA acetyltransferase, *AACT*	2.3.1.9	contig00313|contig11368|FW1NBNE02C59HZ| FW1NBNE02DS7UV|FW1NBNE02EHI1C
HMG-CoA reductase, *HMGR*	1.1.1.34	contig00303|contig01157|contig13725|contig13678| contig00303|contig13324|FW1NBNE02DDW8P| FW1NBNE02DH5GW
Mevalonate kinase, *MVK*	2.7.1.36	contig06070
Phosphomevalonate kinase,*PMK*	2.7.4.2	FW1NBNE02DP41Y
Mevalonate diphosphate decarboxylase, *MVD*	4.1.1.33	contig00126
IPP isomerase, *IPPI*	5.3.3.2	contig02936|contig02937|contig06726|contig06727
Geranylgeranyl pyrophosphate synthase, *GGPPS*	2.5.1.29	contig07732
Farnesyl diphosphate synthase, *FPPS*	2.5.1.10	contig02597|contig10067
Squalene synthase, *SS*	2.5.1.21	contig04447|contig07285
Squalene epoxidase, *SE*	1.14.99.7	contig03811|contig13936|contig09188|contig00132| contig00111|FW1NBNE02DZW52|FW1NBNE02C3G4P|FW1NBNE02C1AS6
Dammarenediol-II synthase, *DS*		contig13785|FW1NBNE02EEIGL|FW1NBNE02DNRCV| FW1NBNE02DNEAO|FW1NBNE02DIULM|FW1NBNE02DEHXE|FW1NBNE02DB02T| FW1NBNE02D0GUT

### SSR detection

Simple sequence repeats (SSRs) are the most feasible genetic markers for plant breeding and genetic applications [34]. A total of 2,772 putative SSR motifs were identified from 2,361 *P. notoginseng *unique sequences with 8.98% (2,772/30,852) frequency (Additional file 4). The frequency of SSRs identified among *P. notoginseng *454-ESTs was similar to that of some dicotyledonous species [35]. These motifs included di-, tri-, tetra-, penta- and hexa-nucleotides with the lengths ranging from 2 to 6 bp. Among the SSR-containing unique sequences, the majority (85.51%) had a single SSR motif in every sequence.

The di-nucleotide motifs were the most abundant in 454-ESTs, which is similar to results obtained from other plants [36]. The occurrence of di- and tri-nucleotide SSR motifs and the number of repeats were presented in Table 4. With a frequency of over sixty percent (67.53%, 1,872/2,772), di-nucleotides were about two times more abundant than tri-nucleotides (25%, 693/2,772), followed by tetra-nucleotides (3.79%, 105/2,772) and penta- and hexa-nucleotides (3.68%, 102/2,772). Among the di-nucleotide repeat classes, AG/GA/CT/TC (57.3%) was the most frequent dimer motif. Other frequent dimer motifs included AT/TA and AC/CA/GT/TG (Table 4). The CG repeats were very infrequent in the plant (0.1%), which is consistent with previous observations [36-38]. Among the tri-nucleotide repeats, AAG/GAA/AGA/CTT/TTC/TCT was the largest repeat class followed by ATC/CAT/TCA/GAT/ATG/TGA and AGC/CAG/GCA/TGC/CTG/GCT (Table 4). Our findings indicated that unique sequences containing SSR markers were indeed abundant in *P. notoginseng*. In particular, several SSR motifs were linked with the unique sequences encoding enzymes (e.g. AACT, HMGR, SE, SS, DS) involved in triterpene saponin biosynthesis (Additional file 5). These unique sequence-derived markers generated in this study represent a valuable genetic resource for future studies of this species as well as related *Panax *species.

**Table 4:** Summary of di- and tri-nucleotide repeats in *P*

Repeat composition	No. of unique sequences (relative percentage)
Dinucleotide	
AC/CA/GT/TG	167 (8.9%)
AG/GA/CT/TC	1073 (57.3%)
AT/TA	630 (33.7%)
CG/GC	2 (0.1%)
**Total of dinucleotide**	**1872 (100%)**

Trinucleotide	
AAC/CAA/ACA/GTT/TTG/TGT	35 (5.1%)
AAG/GAA/AGA/CTT/TTC/TCT	152 (21.9%)
AAT/TAA/ATA/ATT/TTA/TAT	73 (10.5%)
ACC/CAC/CCA/GGT/GTG/TGG	62 (8.9%)
ACG/CGA/GAC/CGT/GTC/TCG	15 (2.2%)
ACT/CTA/TAC/AGT/TAG/GTA	28 (4%)
AGC/CAG/GCA/TGC/CTG/GCT	116 (16.7%)
AGG/GGA/GAG/TCC/CTC/CCT	74 (10.7%)
ATC/CAT/TCA/GAT/ATG/TGA	113 (16.3%)
CCG/CGC/GCC/GGC/GCG/CGG	25 (3.6%)
**Total of trinucleotide**	**693 (100%)**

### Discovery of transcripts encoding putative transcription factors in *P. notoginseng*

Transcription factors, the sequence-specific DNA-binding proteins, play important roles in the regulation of gene expression in response to developmental programs and environmental changes in plants [39]. Based on the searches of automated predictions using Inter-Pro, a total of 906 *P. notoginseng *unique sequences representing putative homologs belonging to different transcription factor (TF) families (Additional file 6), covering the ARF, AUX/IAA, B3, MYB, basic Helix-Loop-Helix (bHLH), bZIP, Homeobox, Homeodomain-like/related, pathogenesis-related/ERF, WRKY and Zinc finger family proteins (Additional file 7). Many protein members of the MYB, bZIP and WRKY transcription factors have been implicated in the regulation of stress responses [39]. The most abundant TF family in *P. notoginseng *454-EST dataset was the MYB family proteins characterized by DNA-binding domains. In *Arabidopsis*, the MYB family, comprising 163 genes, is also one of the largest transcription factor families [40]. The Homeobox proteins were another set of highly expressed transcription factors in *P. notoginseng*. Homeobox genes regulate various developmental aspects in plants, such as the regulation of stem cell specification and organogenesis [41]. The high expression level of MYB and Homeobox proteins in *P. notoginseng *may be linked to response to specific habitats and developmental regulation. Given that the functions of TFs vary in plants, the putative functions of these transcription factors potentially involved in environmental responses and/or developmental regulation in *P. notoginseng *will be characterized in a future study. It is noteworthy that the discovery of these candidate TFs in our 454-EST dataset may provide useful information for future research.

### Candidate genes encoding enzymes involved in the biosynthesis of triterpene saponins

#### Discovery of the transcripts encoding the known enzymes invioved in triterpene saponin biosynthesis

**Figure 4:**
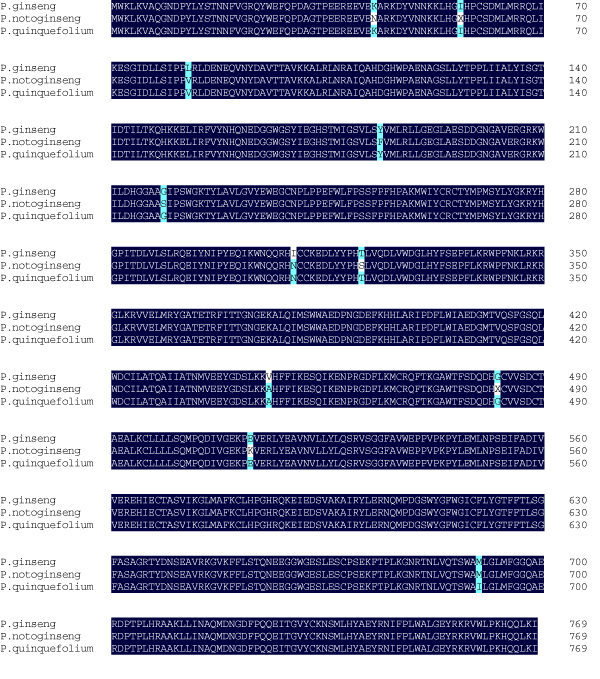
**Alignment of DS amino acid residues from *P. ginseng*, *P. quinquefolium*, and *P. notoginseng***. *P. ginseng *DS and *P. quinquefolius *DS showing 99.5% identity, *P. quinquefolius *DS and *P. notoginseng *DS showing 99.0% identity, *P. ginseng *DS and *P. notoginseng *DS showing 98.7% identity.

Most of all the known enzymes involved in MVA pathway for triterpene saponin biosynthesis were discovered in *P. notoginseng *454-EST dataset (Table 3). As shown in Figure 1, the oxidosqualene is a precursor in the biosynthesis of triterpenoids in higher plants [9]. The cyclization of 2,3-oxidosqualene, catalyzed by OSCs (e.g. DS or AS), is the rate-limited step for triterpene saponin biosynthesis (Figure 1). After the cyclization, the hydroxylation and glycosidation, which are catalyzed by CYP450s and UGTs in turn, play important roles in the production of various triterpene saponins (Figure 1). Dammarane-type saponins are major saponins in *P. notoginseng *root. DS participates in the cyclization of 2,3-oxidosqualene to form the dammarane skeletons in *P. ginseng *[10,14]. It is noteworthy that the transcript for the full-length *P. notoginseng DS *(containing 1,018 reads) was found in the cDNA library. The alignment of amino acid sequences of DS from *P. ginseng*, *P. notoginseng*, and *P. quinquefolius *was shown in Figure 4. DS sequences of *P. quinquefolium *and *P. notoginseng *were deposited in NCBI GenBank with Accession No. GU997679 and GU997680, respectively. All three DSs have 769 amino acid residues and display high similarity. At the protein level, DSs exhibit 99.5% identity between *P. ginseng *and *P. quinquefolius*, 99.0% identity between *P. quinquefolius *and *P. notoginseng*, and 98.7% identity between *P. ginseng *and *P. notoginseng*, indicating that *P. ginseng *and *P. quinquefolius *are more closely related. This conclusion agrees with that drawn from the alignment of *TrnK *and 18S rRNA sequences [42].

The oleanane-type saponin (Ro) has not been detected in *P. notoginseng *based on the phytochemical studies [2]. Surprisingly, two *P. notoginseng *singleton sequences (FW1NBNE02D50IR and FW1NBNE02D30JP) matched to *β-AS *of *Panax ginseng*. The presence of the *β-ASs *in *P. notoginseng *was further confirmed by RT-PCR and the PCR products were sequenced (Data not shown). The existence of the transcripts for *β-ASs *in *P. notoginseng *was seemingly conflicted with previous reports claiming that oleanane-type ginsenosides do not exist in *P. notoginseng *[2,43]. Therefore, we presumed that either oleanane-type ginsenosides were present in *P. notoginseng *at levels too low to be detected phytochemically, or oleanane-type ginsenosides in fact did not exist in *P. notoginseng*, despite the presence of *β-AS*, might due to the lack of biosynthetic genes downstream of β-AS.

#### Discovery of the candidate CYP450s and UGTs might be involved in triterpene saponin biosynthesis by phylogenetic analysis

Characterization of specific CYP450s or UGTs involved in triterpene saponin biosynthesis in *Panax *genus will facilitate to elucidation of the triterpene saponin biosynthetic pathway. CYP450s are generally involved in the biosynthesis of terpenoids, sterols, lignins, hormones, fatty acids, pigments, and phytoalexins in plants [44]. Some CYP450s are proposed to participate in the oxidation of the dammarane skeleton at C-12 and the other at C-6 toward the production of protopanaxadiol and protopanaxatriol, respectively [10,45]. Previous studies have characterized CYP88D6 from *Glycyrrhiza uralensis *(CYP85 clan) [23] and CYP93E1 from *Glycine max *(CYP71 clan) [46], both of which were involved in triterpene saponin biosynthesis. Therefore, the CYP450s belonging to CYP85 and CYP71 clan might be involved in ginsenoside biosynthesis in *Panax *genus. Glycosylation, catalyzed by glycosyltransferases (GTs), transfers the activated saccharides to an aglycone substrate in the modification on ginsenoside biosynthesis. This enzymatic conjugation based on glycosylation can stabilize the product and alter its physiological activity [47]. The dammarane- and oleanane-type aglycones have ginsenoside bioactivity after the glycosylation catalyzed by UGTs. UGT73K1 and UGT71G1 from *Medicago truncatula *[48] and UGT74M1 from *Saponaria vaccaria *[22] have been identified and characterized with functions in triterpene saponin biosynthesis. Recently, several CYP450s and UGTs were found as candidate genes involved in ginsenoside biosynthesis in *P. quinquefolius *and *P. ginseng *in our previous studies [15,29]. Particularly, one CYP450 (contig00248) and four UGTs (contig01001, contig14976, contig15451, and contig16321) were selected as candidate genes most likely to be involved in ginsenoside biosynthesis based on their MeJA-inducible and tissue specific expression patterns in *P. quinquefolius *[15].

In this study, a total of 174 CYP450 (Additional file 8) and 242 GT (Additional file 9) unique sequences were found in the *P. notoginseng *cDNA library. As reported for *P. ginseng *and other plants, enzymes in the same biosynthetic pathway are usually co-expressed [14,48,49]. Hence, *DS *was expressed abundantly (with 1,018 reads) in the root of *P. notoginseng*, indicating that other genes in the triterpene saponin biosynthetic pathway might also expressed at higher levels in the *P. notoginseng *root. Based on this knowledge, CYP450- and UGT-unique sequences that contain more than 10 reads from the *P. notoginseng *root cDNA library were found as candidate enzymes involved in triterpene saponin biosynthesis. Thus, 25 *CYP450*s and 16 *UGT*s were selected, among which 15 *CYP450*s and 8 *UGT*s had full-length sequences after assembly and using RACE (rapid amplification of cDNA end) method. The primers used for RACE were listed in Additional file 10. These cDNA sequences have been submitted to the NCBI database and given accession number GU997664-GU997678 for *CYP450*s and GU997656-GU997663 for *UGT*s.

**Figure 5:**
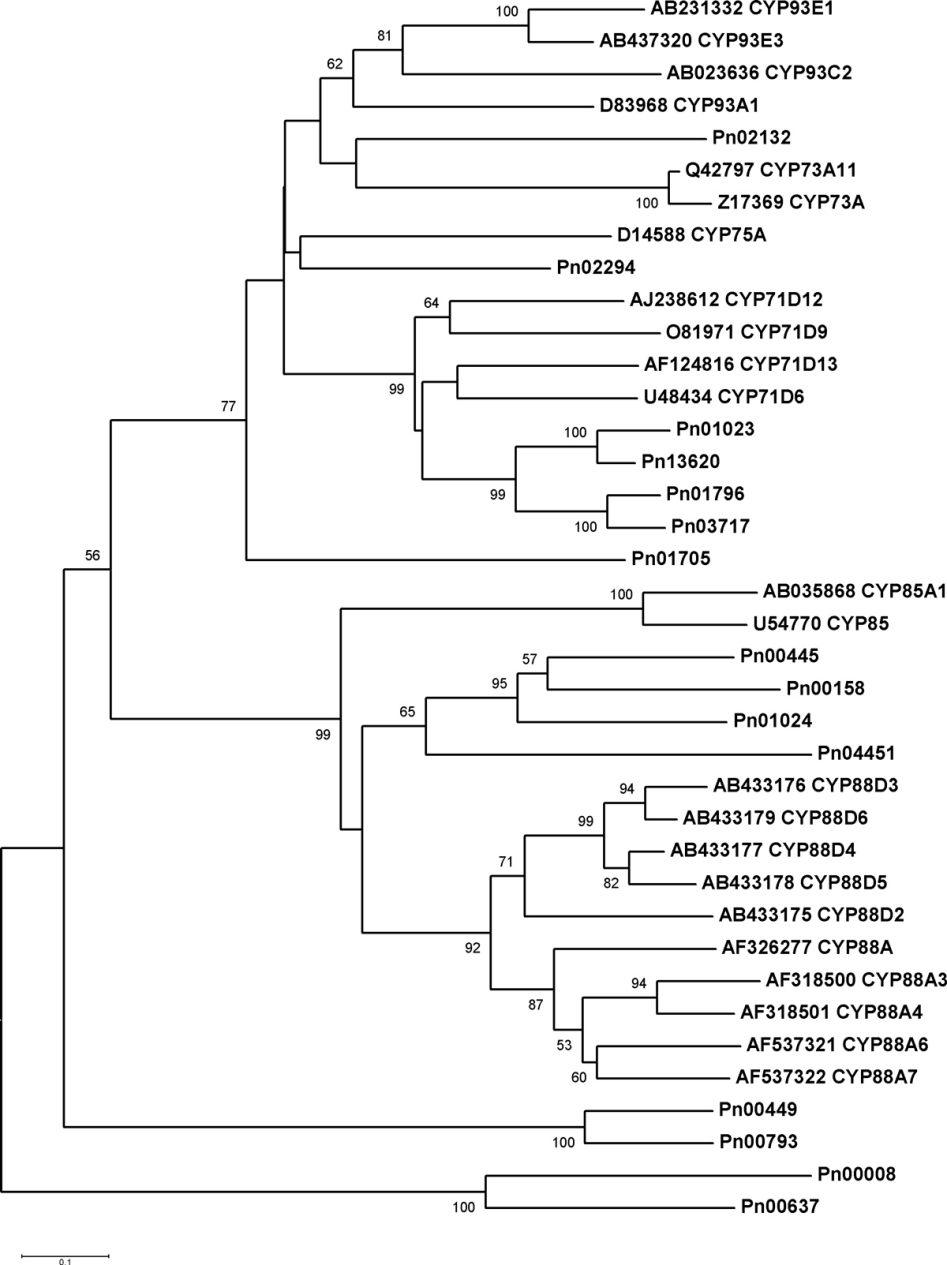
**Phylogenetic analysis of full-length CYP450s from *P. notoginseng *and characterized CYP450s from other plants**. Amino acid sequences were aligned using the CLUSTALW program, and evolutionary distances were computed using MEGA4 with the Poisson correction method. Bootstrap values obtained after 1000 replications are indicated on the branches. Values less than 50% are not shown. The GenBank/EMBL/DDBJ accession numbers of the sequences are: U48434 (*Solanum chacoense*), O81971 (*Glycine max*), AJ238612 (*Catharanthus roseus*), AF124816 (*Mentha × piperita*), Z17369 (*Helianthus tuberosus*), Q42797 (*Glycine max*), D14588 (*Petunia × hybrida*), AB035868 (*Arabidopsis thaliana*), U54770 (*Solanum lycopersicum*), AB433175 (*Medicago truncatula*), AB433176 (*Medicago truncatula*), AB433177 (*Lotus japonicus*), AB433178 (*Lotus japonicus*), AB433179 (*Glycyrrhiza uralensis*), AF537321 (*Pisum sativum*), AF537322 (*Pisum sativum*), AF318500 (*Arabidopsis thaliana*), AF318501 (*Arabidopsis thaliana*), AF326277 (*Hordeum vulgare*), AF135485 (*Glycine max*), AB231332 (*Glycine max*), AB023636 (*Glycyrrhiza echinata*), AB437320 (*Glycyrrhiza uralensis*), D83968 (*Glycine max*), X71657 (*Solanum melongena*), X71658 (*Solanum melongena*), X71656 (*Solanum melongena*), X71655 (*Solanum melongena*), L23209 (*Zea mays*), NM_001112599 (*Zea mays*), U29333 (*Pisum sativum*), U69134 (*Arabidopsis thaliana*), U18929 (*Arabidopsis thaliana*), AF150881 (*Solanum lycopersicum × Solanum peruvianum*), AF214008 (*Brassica napus*), U38416 (*Arabidopsis thaliana*), AF029856 (*Sorghum bicolor*), AF029858 (*Sorghum bicolor*), AJ583531 (*Triticum aestivum*).

The phylogenetic relationship between the15 full-length CYP450s of *P. notoginseng *and characterized CYP450s from other plants was depicted in Figure 5. Four CYP450s (Pn00445, Pn00158, Pn01024, and Pn04451) belonged to the group representing the CYP85 clan which comprises CYP88D6 from *G. uralensis*, a licorice β-amyrin 11-oxidase with a key role in the biosynthesis of the triterpene sweetener glycyrrhizin [23] (Figure 5). Thus, the four CYP450s constituted a subgroup and were related to CYP88D6 (Figure 5). Interestingly, the transcript of Pn00158 had high identity to the *P. quinquefolius *candidate CYP450 (contig00248) [15] (Data not shown), which was likely to be involved in ginsenoside biosynthesis. Seven CYP450s (Pn02132, Pn02294, Pn01023, Pn13620, Pn01796, Pn03717, and Pn01705) belonged to the group representing the CYP71 clan (Figure 5). It is noteworthy that Pn02132 was phylogenetically close to CYP93E1 (Figure 5), which was the first triterpene hydroxylase identified from a plant species [46]. Therefore, Pn02132 and Pn00158 were lead candidate CYP450s involved in triterpene saponin biosynthesis.

**Figure 6:**
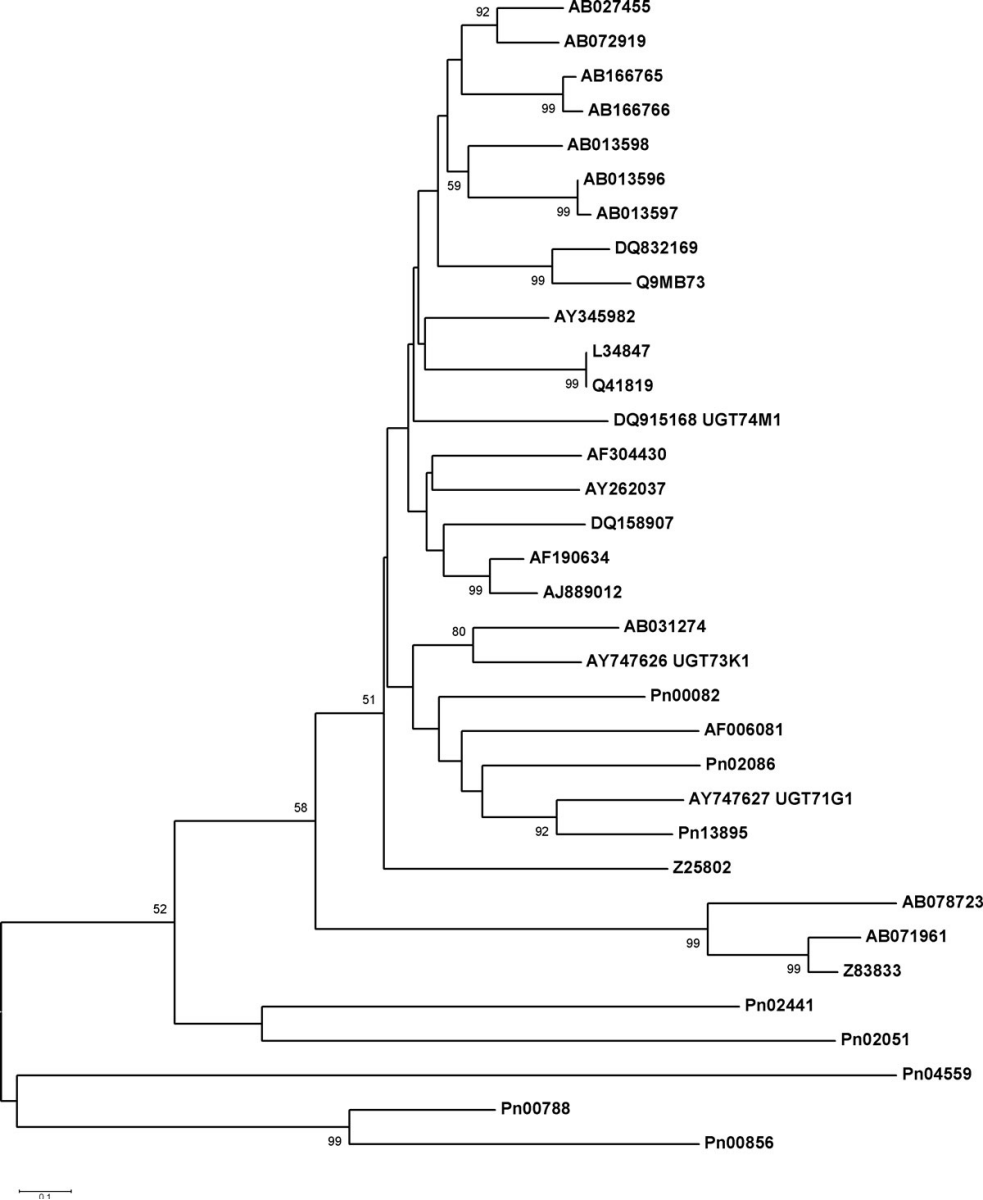
**Phylogenetic analysis between full-length UGTs of *P. notoginseng *and characterized UGTs from other plants**. Amino acid sequences were aligned using the program CLUSTALW and evolutionary distances were computed using the Poisson correction method with MEGA4. Bootstrap values obtained after 1000 replications are indicated on the branches. Values less than 50% are not shown. The GenBank/EMBL/DDBJ accession numbers of the sequences are AY747627 (*Medicago truncatula*), AY747626 (*Medicago truncatula*), DQ915168 (*Vaccaria hispanica*), AB013598 (*Glandularia × hybrida*), AB027455 (*Petunia × hybrida*), AB013597 (*Perilla frutescens *var. *crispa*), AB013596 (*Perilla frutescens *var. *crispa*), AY262037 (*Crocus sativus*), AY345982 (*Stevia rebaudiana*), Q41819 (*Zea mays*), DQ158907 (*Arabidopsis thaliana*), Q9MB73 (*Citrus unshiu*), AB166766 (*Eucalyptus perriniana*), AB166765 (*Eucalyptus perriniana*), AJ889012 (*Solanum lycopersicum*), AB072919 (*Nicotiana tabacum*), DQ832169 (*Vitis labrusca*), AF190634 (*Nicotiana tabacum*), AF304430 (*Brassica napus*), AB078723 (*Aspergillus oryzae*), Z83833 (*Arabidopsis thaliana*), AB071961 (*Panax ginseng*), AB031274 (*Scutellaria baicalensis*), Z25802 (*Petunia × hybrida*), L34847 (*Zea mays*).

Phylogenetic analysis using an unrooted distance tree showed the relationship of *P. notoginseng *UGT sequences to other functionally characterized members of plant UGT families (Figure 6). Three UGTs (Pn00082, Pn02086, and Pn13895) belonged to the group consisting of triterpene glycosyltransferases (UGT73K1, UGT71G1) from *Medicago truncatula *(Figure 6). Among them, Pn13895 was regarded as a lead candidate UGT responsible for triterpene saponin biosynthesis, because of its close relation to UGT71G1(Figure 6). Characterization of these candidate CYP450s and UGTs will pave the way to illustrate the biosynthetic pathways of triterpene saponins in *P. notoginseng *and other related *Panax *species. Although many of candidate genes involved in triterpene saponin biosynthesis were discovered in this study, the functional identification of these genes has not be carried out in this study. They will be the focus of study in the future

## Conclusions

In this study, a large-scale 454-EST investigation of *P. notoginseng *root was performed based on 454 pyrosequencing. This 454-EST dataset from *P. notoginseng *root contribute significantly to provide a large number of transcripts for gene discovery in this medicinal plant. The description of the expressed genes and distribution of gene functions was illustrated according to GO analysis and KEGG assignment. A number of genes involved in triterpene saponin biosynthesis, including cytochrome P450s and glycosyltransferases, were discovered in our EST dataset. More importantly, a handful of candidate CYP450s and UGTs that are most likely to be involved in the biosynthesis of triterpene saponins were found based on phylogenetic analysis. Many transcription factors and EST-SSR markers were identified as well. These data will provide comprehensive information on gene discovery, transcriptome profiling, transcriptional regulation, and molecular markers for *P. notoginseng*. This study will contribute to further improvements on this medicinal plant through marker-assisted breeding or genetic engineering on this species as well as for other medicinal plants in the *Araliaceae *family.

## Methods

### Plant material

The 4-year-old *Panax notoginseng *cultivated on farms was routinely harvested for medical purposes. The *P. notoginseng *was collected from the fields of Wenshan County, Yunnan Province, China. After cleaning, the root tissues were cut into small pieces and immediately frozen in liquid nitrogen, and stored at -80°C until further processing.

### RNA preparation

Total RNA was isolated using the Plant RNA Isolation Mini Kit (BioTeke, Beijing, China). The total RNA was treated with DNase I (TURBO DNase; Ambion, USA) at a concentration of 1.5 units/μg of total RNA. The RNA quality was tested using 1% ethidium bromide-stained (EtBr-stained) agarose gels and the concentration was assessed using a GeneQuant100 spectrophotometer (GE Healthcare, UK) prior to cDNA synthesis.

### cDNA synthesis and 454 pyrosequencing

The first-strand cDNA was produced using 2.1 μg of total RNA extracted from the root of *P. notoginseng *according to the instructions provided with Clontech's SMART cDNA synthesis kit (Clontech, USA) with slight modifications as our previous study [15]: in order to remove the long poly(A/T) tails in cDNA sequences, a modified synthetic poly (T) primer (5'-AAG CAG TGG TAT CAA CGC AGT GCA GT(20)VN-3') containing a *Bsg*I digestion site upstream of the poly (T) segment was used in combination with the Clontech SMART IV primer to synthesize the first-strand cDNA. The cDNA was amplified using PCR Advantage II polymerase (Clontech, USA) to synthesize the double-strand cDNA (ds cDNA) with the following thermal profile: 1 min at 95°C followed by 19 cycles of 95°C for 15 sec, 65°C for 30 sec, and 68°C for 6 min. And then, 5 μl of PCR product were electrophoresed in a 1% agarose gel to determine the amplification efficiency and quality. Approximately 13 μg of amplified ds cDNA was purified using the PureLink ™ PCR purification kit (Invitrogen, USA). Then the purified cDNA was treated with *Bsg*I (NEB, USA) overnight at 37°C and recovered by QIAquick PCR Purification Kit (Qiagen, USA). Finally, a total of 10 μg of ds cDNA was used for pyrosequencing with the GS FLX Titanium Kit.

### 454 EST assembly and annotation

The 454 raw read sequences were screened and trimmed for weak signals by GS FLX pyrosequencing software to yield high-quality (HQ) (> 99.5% accuracy of single-base reads) sequences. The resulting HQ reads were then submitted to the Short Read Archive at NCBI and assigned the accession number SRX017444. The primer and adapter sequences were trimmed from the HQ sequences, followed by removing the sequences shorter than 50 bp from the clean ESTs before assembly. Then, the data from the 454 read sequences were assembled into unique sequences (including contigs and singletons) using 454 GS *De Novo *Assembler software v2.0.01.14 (454 Life Sciences, Roche) with a quality score threshold set at 40.

The assembled unique sequences were first searched for sequence similarities against the NCBI non-redundant nucleotide (Nt) database using the BLASTN algorithm with *E-*value cut-off of 10^-5 ^to find and remove ribosomal RNA sequences [50]. And then, the remaining sequences were searched against the public databases including the *Arabidopsis *protein database at The Arabidopsis Information Resource (TAIR; http://www.arabidopsis.org) (version Tair9), SwissProt protein database (http://www.expasy.ch/sprot; released on 06/19/2009), and the NCBI non-redundant protein (Nr) database (http://www.ncbi.nlm.nih.gov; released on 06/23/2009) using the BLASTX algorithm with an E-value cut-off of 10^-5^. The functional categories of these unique sequences were further analyzed using the Gene Ontology (GO) database. The unique sequences were categorized according to GO terms based on AGI codes and TAIR GO slim provided by TAIR.

### Pathway assignment with KEGG database

Pathway assignments were carried out according to the Kyoto Encyclopedia of Genes and Genome (KEGG) mapping (http://www.genome.ad.jp/kegg/kegg2.html) (version KEGG 50) [33]. Enzyme commission (EC) numbers were assigned to unique sequences after BLASTX searches with an *E-*value cut-off of 10^-5 ^upon against the KEGG database. The unique sequences were assigned to special biochemical pathways according to the corresponding EC distribution in the KEGG database.

### Simple sequence repeat (SSR) detection

The total unique sequences were searched to determine the composition, frequency, and distribution of simple sequence repeats (SSRs) using an online SSR identification tool - SSRIT (Simple Sequence Repeat Identification Tool) (http://www.gramene.org/db/markers/ssrtool) [51]. The search parameters for the maximum motif-length group were set to recognize hexamers and the minimum number of repeats was set to five.

### Screening of CYP450 or UGT unique sequences encoding enzymes responsible for triterpene saponin biosynthesis

The *DS *transcript was much more abundant in *P. notoginseng *(1,018 reads), suggesting the other genes encoding enzymes in the same biosynthetic pathway were also expressed at much higher levels in the former species. For CYP450s and UGTs, each isozyme with more than ten reads in *P. notoginseng *is arbitrarily considered a candidate involved in the biosynthesis of triterpene saponins. The screening of CYP450s and UGTs was performed according to phylogenetic analysis.

### Production of full-length cDNA sequences for CYP450s and UGTs using RACE technology

Primers listed in Additional file 10 were synthesized according to selected CYP450s and UGTs EST sequences. The 5' or 3' ends of cDNAs were amplified using a SMART RACE cDNA amplification kit (Clontech, USA) from total RNA of *P. notoginseng *root and cloned into T easy Vector (Promega, USA) for Sanger sequencing. Then the full-length cDNA of each gene was generated by assembly of the corresponding EST sequence and 5' and/or 3'end sequences.

### Phylogenetic analysis

Distances between each clone were calculated with the CLUSTAL W program [52]. The indicated scale represents 0.1 amino acid substitutions per site. Amino acid sequences were aligned using the CLUSTAL W program and evolutionary distances were computed using the Poisson correction method, and a Neighbor-Joining (NJ) tree was constructed with MEGA4. Bootstrap values obtained after 1000 replications are given on the branches. Values less than 50% are not shown.

## List of abbreviations

β-AS: β-amyrin synthase; BLAST: basic local alignment search tool; bp: base pair; cDNA: complementary DNA; CYP450: cytochrome P450; DS: dammarenediol-II synthase; EST: expressed sequence tag; GGDP: geranylgeranyl diphosphate; GO: gene ontology; GT: glucosyltransferase; KEGG: Kyoto encyclopedia of genes and genomes; MeJA: methyl jasmonate; NCBI: National Center for Biotechnology Information; NGS: next-generation sequencing; RACE: rapid amplification of cDNA end; SSR: simple sequence repeat; TAIR: the *Arabidopsis *Information Resource; UGT: UDP- glucosyltransferase.

## Competing interests

The authors declare that they have no competing interests.

## Authors' contributions

HML contributed to the cDNA sample preparation, data analysis and manuscript writing. CS and YZS participated in the design of the study, the tissue sample collection, and the data analysis. QW helped with RNA extraction, gene cloning and the generation of the phylogenetic tree for CYP450s and UGTs. YL contributed to the bioinformatic analysis and helped with the RNA extraction. YYN helped with RNA extraction and cDNA amplification. JYS, XLC, HXX, CYL, JYL and AS participated in the study design and discussed the results. This work was conducted in the laboratory of SLC, who initiated the 454-sequencing projects and contributed to the evaluation and discussion of the results as well as to the revision of the manuscript. All authors have read and approved the final manuscript.

## Supplementary Material

Additional file 1 **Summary of the annotation percentage of *P. notoginseng *454-ESTs as compared to public databases**. The annotation for *P. notoginseng *unique sequences was based on sequence similarity searches against public databases including SwissProt, KEGG, TAIR, NCBI non-redundant protein (Nr), and NCBI non-redundant nucleotide (Nt) database. A total of 70.2% of *P. notoginseng *unique sequences were annotated by BLAST searches against the above public databases. Click here for file

Additional file 2 **Mapping of *P. notoginseng *unique sequences to KEGG biochemical pathways**. List of the number of *P. notoginseng *unique sequences involved in metabolism, genetic information processing, environmental information processing, cellular processes, human diseases, unclassified and unassigned in the 454-EST dataset. Click here for file

Additional file 3 **The *P. notoginseng *unique sequences involved in the biosynthesis of secondary metabolites**. The number of unique sequences involved in the biosynthesis of alkaloid, brassinosteroid, caffeine, carotenoid, diterpenoid, flavone and flavonol, flavonoid, limonene and pinene, monoterpenoid, novobiocin, phenylpropanoid, streptomycin, terpenoid, tetracycline and zeatin. Click here for file

Additional file 4 **The detection of SSR motifs in the unique sequences of *P. notoginseng***. The list of unique sequences (including contigs and singletons) containing potential microsatellite loci including the name of unique sequence, motif (SSR-repeat type), number of repeat, SSR start, SSR end and sequence length from *P. notoginseng *454-EST dataset. Click here for file

Additional file 5 **The discovery of SSR motifs in the putative triterpene saponin-biosynthetic genes**. The SSR motifs were detected in the putative triterpene saponin-biosynthetic genes including *AACT *(acetyl-CoA acetyltransferase), *HMGR *(HMG-CoA reductase), *SS *(squalene synthase), *SE *(squalene epoxidase) and *DS *(dammarenediol-II synthase). Click here for file

Additional file 6 **The *P. notoginseng *unique sequences encoding putative transcription factors based on Inter-Pro searches**. The 906 unique sequences of *P. notoginseng *containing transcription factor domains using Inter-Pro searches. Click here for file

Additional file 7 **Major transcription factor families identified from *P. notoginseng *using Inter-Pro**. The unique sequences from *P. notoginseng *with similarities to genes encoding transcription factors. Click here for file

Additional file 8 **Cytochrome P450 discovery**. The unique sequences from *P. notoginseng *with sequence similarities to cytochrome P450s. Click here for file

Additional file 9 **Glycosyltransferase discovery**. The unique sequences from *P. notoginseng *with sequence similarities to glycosyltransferase. Click here for file

Additional file 10 **The primers used for RACE in this study**. The primers used in 5'-RACE for the amplification of unique sequences including Pn01024, Pn02132, Pn03717, and Pn00788. Click here for file
