# Supplementary material for: Analysis of the transcriptome of Panax notoginseng root uncovers putative triterpene saponin-biosynthetic genes and genetic markers
Source: BMC Genomics. 2011 Dec 23;12(Suppl 5):S5. doi: 10.1186/1471-2164-12-S5-S5 (PMC3287501; doi:10.1186/1471-2164-12-S5-S5)
Supplement: Additional file 1 — Summary of the annotation percentage of P. notoginseng 454-ESTs as compared to public databases. The annotation for P. notoginseng unique sequences was based on sequence similarity searches against public databases including SwissProt, KEGG, TAIR, NCBI non-redundant protein (Nr), and NCBI non-redundant nucleotide (Nt) database. A total of 70.2% of P. notoginseng unique sequences were annotated by BLAST searches against the above public databases. [file 1471-2164-12-S5-S5-S1.doc]

**Additional file 1 Summary of the annotation percentage of *P. notoginseng* 454-EST as compared to public databases**

| Database | No. of unique sequences | Annotation percentage (%) |
| --- | --- | --- |
| TAIR | 18,813 | 61.0 |
| SwissProt | 10,355 | 33.6 |
| KEGG | 16,300 | 52.8 |
| Nr | 20,188 | 65.4 |
| Nt | 17,954 | 58.2 |
| **Total annotated** | **21,672** | **70.2** |
| Total unique sequences | 30,852 |  |
