# Supplementary material for: Analysis of the transcriptome of Panax notoginseng root uncovers putative triterpene saponin-biosynthetic genes and genetic markers
Source: BMC Genomics. 2011 Dec 23;12(Suppl 5):S5. doi: 10.1186/1471-2164-12-S5-S5 (PMC3287501; doi:10.1186/1471-2164-12-S5-S5)
Supplement: Additional file 2 — Mapping of P. notoginseng unique sequences to KEGG biochemical pathways. List of the number of P. notoginseng unique sequences involved in metabolism, genetic information processing, environmental information processing, cellular processes, human diseases, unclassified and unassigned in the 454-EST dataset. [file 1471-2164-12-S5-S5-S2.doc]

**Additional file 2 Mapping of *P*. *notoginseng*** unique sequences to KEGG biochemical pathways

| **KEGG categories represented** | **No. of unique sequences** |
| --- | --- |
| **Metabolism** | **1,349** |
| Amino Acid Metabolism | 306 |
| Biosynthesis of Polyketides and Non-ribosomal Peptides | 10 |
| Biosynthesis of Secondary Metabolites | 131 |
| Carbohydrate Metabolism | 295 |
| Energy Metabolism | 145 |
| Glycan Biosynthesis and Metabolism | 76 |
| Lipid Metabolism | 211 |
| Metabolism of Cofactors and Vitamins | 104 |
| Metabolism of Other Amino Acids | 17 |
| Nucleotide Metabolism | 50 |
| Xenobiotic Biodegradation and Metabolism | 4 |
| **Genetic Information Processing** | **735** |
| Folding, Sorting, and Degradation | 255 |
| Replication and Repair | 73 |
| Transcription | 84 |
| Translation | 323 |
| **Environmental Information Processing** | **134** |
| Membrane Transport | 31 |
| Signal Transduction | 92 |
| Signaling Molecules and Interaction | 11 |
| **Cellular Processes** | **355** |
| Behavior | 1 |
| Cell Communication | 65 |
| Cell Growth and Death | 81 |
| Cell Motility | 25 |
| Endocrine System | 133 |
| Immune System | 40 |
| **Protein Families** | **776** |
| Cellular Processes and Signaling | 179 |
| Genetic Information Processing | 466 |
| Metabolism | 131 |
| **Human Diseases** | **200** |
| **Unclassified** | **323** |
| **Unassigned^a^** | **12,438** |

^a^Unassigned unique sequences are those that have significant similarities to known sequences in the KEGG database, but whose functions in biochemical pathways are unclear.
