# Supplementary material for: Analysis of the transcriptome of Panax notoginseng root uncovers putative triterpene saponin-biosynthetic genes and genetic markers
Source: BMC Genomics. 2011 Dec 23;12(Suppl 5):S5. doi: 10.1186/1471-2164-12-S5-S5 (PMC3287501; doi:10.1186/1471-2164-12-S5-S5)
Supplement: Additional file 3 — The P. notoginseng unique sequences involved in the biosynthesis of secondary metabolites. The number of unique sequences involved in the biosynthesis of alkaloid, brassinosteroid, caffeine, carotenoid, diterpenoid, flavone and flavonol, flavonoid, limonene and pinene, monoterpenoid, novobiocin, phenylpropanoid, streptomycin, terpenoid, tetracycline and zeatin. [file 1471-2164-12-S5-S5-S3.doc]

**Additional file 3 The *P*. *notoginseng* unique sequences involved in the biosynthesis of secondary metabolites**
