# Supplementary material for: Analysis of the transcriptome of Panax notoginseng root uncovers putative triterpene saponin-biosynthetic genes and genetic markers
Source: BMC Genomics. 2011 Dec 23;12(Suppl 5):S5. doi: 10.1186/1471-2164-12-S5-S5 (PMC3287501; doi:10.1186/1471-2164-12-S5-S5)
Supplement: Additional file 4 — The detection of SSR motifs in the unique sequences of P. notoginseng. The list of unique sequences (including contigs and singletons) containing potential microsatellite loci including the name of unique sequence, motif (SSR-repeat type), number of repeat, SSR start, SSR end and sequence length from P. notoginseng 454-EST dataset. [file 1471-2164-12-S5-S5-S4.doc]

**Additional file 4 The detection of SSR motifs in the unique sequences of *P. notoginseng***

| **Unique sequence** | **Motif** | **No.of repeats** | **SSR start** | **SSR end** | **Sequence length** |
| --- | --- | --- | --- | --- | --- |
| contig09425-1 | aaaacc | 5 | 1 | 30 | 218 |
| FW1NBNE02DC7MX-1 | aaac | 6 | 76 | 99 | 433 |
| contig00093-1 | aaacc | 6 | 187 | 216 | 665 |
| FW1NBNE02DJO3K-1 | aaacc | 5 | 2 | 26 | 390 |
| contig02076-1 | aaag | 5 | 193 | 212 | 775 |
| contig11388-1 | aaag | 5 | 163 | 182 | 609 |
| contig00099-1 | aaat | 5 | 986 | 1005 | 1251 |
| FW1NBNE02D4TDD-1 | aaat | 5 | 16 | 35 | 102 |
| FW1NBNE02EDQJ2-2 | aac | 5 | 214 | 228 | 375 |
| FW1NBNE02DNHXO-1 | aac | 6 | 132 | 149 | 186 |
| FW1NBNE02D97KA-1 | aac | 9 | 66 | 92 | 474 |
| FW1NBNE02ECSRZ-1 | aac | 8 | 269 | 292 | 543 |
| FW1NBNE02DM1VG-1 | aac | 5 | 390 | 404 | 502 |
| FW1NBNE02DP18C-1 | aacc | 5 | 12 | 31 | 472 |
| FW1NBNE02DATJU-1 | aact | 5 | 48 | 67 | 69 |
| contig05913-2 | aag | 6 | 505 | 522 | 567 |
| FW1NBNE02DD42P-2 | aag | 5 | 435 | 449 | 450 |
| FW1NBNE02ESAEF-2 | aag | 7 | 135 | 155 | 168 |
| contig00080-1 | aag | 8 | 45 | 68 | 315 |
| contig00134-1 | aag | 6 | 195 | 212 | 1338 |
| contig00765-1 | aag | 5 | 328 | 342 | 559 |
| contig03958-1 | aag | 5 | 883 | 897 | 952 |
| contig04027-1 | aag | 6 | 139 | 156 | 722 |
| contig04999-1 | aag | 10 | 2927 | 2956 | 3010 |
| contig06831-1 | aag | 5 | 181 | 195 | 795 |
| contig07624-1 | aag | 5 | 140 | 154 | 165 |
| contig12373-1 | aag | 5 | 19 | 33 | 471 |
| contig12565-1 | aag | 5 | 169 | 183 | 365 |
| FW1NBNE02EIDX6-1 | aag | 6 | 313 | 330 | 438 |
| FW1NBNE02DR3FU-1 | aag | 7 | 451 | 471 | 529 |
| FW1NBNE02DMUMO-1 | aag | 5 | 40 | 54 | 503 |
| FW1NBNE02DU2J2-1 | aag | 6 | 130 | 147 | 529 |
| FW1NBNE02D6XVO-1 | aag | 5 | 86 | 100 | 238 |
| FW1NBNE02DXJ5E-1 | aag | 5 | 57 | 71 | 519 |
| FW1NBNE02D9BHM-1 | aag | 9 | 215 | 241 | 365 |
| contig02913-1 | aagaca | 5 | 6 | 35 | 170 |
| contig01480-1 | aaggg | 5 | 191 | 215 | 1945 |
| contig03531-2 | aat | 6 | 836 | 853 | 1295 |
| FW1NBNE02D85XI-3 | aat | 6 | 263 | 280 | 517 |
| contig00844-1 | aat | 5 | 498 | 512 | 757 |
| contig00992-1 | aat | 8 | 200 | 223 | 1206 |
| contig02016-1 | aat | 6 | 77 | 94 | 646 |
| contig03376-1 | aat | 7 | 1 | 21 | 709 |
| FW1NBNE02DBFSC-1 | aat | 5 | 318 | 332 | 378 |
| FW1NBNE02DVNMQ-1 | aat | 6 | 315 | 332 | 389 |
| FW1NBNE02ES9RG-1 | aat | 7 | 30 | 50 | 552 |
| FW1NBNE02DWSMQ-1 | aat | 6 | 129 | 146 | 529 |
| FW1NBNE02ESAEF-1 | aat | 6 | 117 | 134 | 168 |
| FW1NBNE02EGT97-1 | aat | 6 | 222 | 239 | 495 |
| FW1NBNE02DPHPG-1 | aat | 5 | 318 | 332 | 453 |
| FW1NBNE02EEED5-1 | aat | 6 | 24 | 41 | 386 |
| FW1NBNE02DZIH5-1 | aat | 5 | 187 | 201 | 391 |
| FW1NBNE02C7NVN-1 | aat | 6 | 160 | 177 | 436 |
| FW1NBNE02DU74M-1 | aat | 7 | 166 | 186 | 474 |
| FW1NBNE02EL5X2-1 | aat | 5 | 37 | 51 | 225 |
| contig10080-1 | aatgg | 6 | 230 | 259 | 462 |
| contig03928-2 | ac | 5 | 1481 | 1490 | 1495 |
| contig09466-2 | ac | 5 | 150 | 159 | 527 |
| contig10789-2 | ac | 5 | 342 | 351 | 487 |
| contig12320-2 | ac | 5 | 247 | 256 | 697 |
| contig12587-2 | ac | 5 | 70 | 79 | 267 |
| contig13274-2 | ac | 5 | 139 | 148 | 149 |
| FW1NBNE02EN3QL-2 | ac | 8 | 24 | 39 | 55 |
| FW1NBNE02DVYHQ-2 | ac | 5 | 72 | 81 | 509 |
| FW1NBNE02CZUT9-2 | ac | 10 | 128 | 147 | 154 |
| FW1NBNE02D1Z3L-2 | ac | 5 | 273 | 282 | 297 |
| FW1NBNE02DDNTH-2 | ac | 9 | 27 | 44 | 75 |
| FW1NBNE02ENM0R-2 | ac | 7 | 117 | 130 | 134 |
| FW1NBNE02CZOSL-2 | ac | 5 | 44 | 53 | 177 |
| FW1NBNE02DEQL5-3 | ac | 7 | 207 | 220 | 221 |
| FW1NBNE02C8CU1-3 | ac | 8 | 74 | 89 | 93 |
| FW1NBNE02C5TD5-3 | ac | 5 | 119 | 128 | 267 |
| contig00096-1 | ac | 5 | 101 | 110 | 491 |
| contig00553-1 | ac | 5 | 9 | 18 | 1590 |
| contig02452-1 | ac | 5 | 59 | 68 | 465 |
| contig02649-1 | ac | 5 | 11 | 20 | 1107 |
| contig03109-1 | ac | 5 | 8 | 17 | 535 |
| contig03209-1 | ac | 6 | 1516 | 1527 | 1612 |
| contig03656-1 | ac | 5 | 104 | 113 | 541 |
| contig03853-1 | ac | 5 | 886 | 895 | 1405 |
| contig04179-1 | ac | 5 | 212 | 221 | 285 |
| contig04460-1 | ac | 5 | 19 | 28 | 645 |
| contig04866-1 | ac | 6 | 1679 | 1690 | 1736 |
| contig08149-1 | ac | 9 | 146 | 163 | 622 |
| contig08384-1 | ac | 5 | 628 | 637 | 742 |
| contig08730-1 | ac | 5 | 53 | 62 | 506 |
| contig09285-1 | ac | 8 | 375 | 390 | 432 |
| contig09891-1 | ac | 5 | 126 | 135 | 746 |
| contig11874-1 | ac | 7 | 41 | 54 | 546 |
| contig12409-1 | ac | 7 | 329 | 342 | 461 |
| contig12716-1 | ac | 7 | 357 | 370 | 446 |
| contig13694-1 | ac | 23 | 195 | 240 | 286 |
| FW1NBNE02C6YQL-1 | ac | 22 | 39 | 82 | 121 |
| FW1NBNE02DURZJ-1 | ac | 10 | 169 | 188 | 239 |
| FW1NBNE02DPZ1P-1 | ac | 23 | 8 | 53 | 85 |
| FW1NBNE02EDG0Y-1 | ac | 6 | 7 | 18 | 289 |
| FW1NBNE02D5RPG-1 | ac | 5 | 63 | 72 | 483 |
| FW1NBNE02D8AD2-1 | ac | 5 | 298 | 307 | 440 |
| FW1NBNE02DEZD5-1 | ac | 6 | 73 | 84 | 142 |
| FW1NBNE02C521M-1 | ac | 8 | 88 | 103 | 224 |
| FW1NBNE02EW71D-1 | ac | 8 | 85 | 100 | 183 |
| FW1NBNE02D3C6T-1 | ac | 6 | 44 | 55 | 482 |
| FW1NBNE02DJUR9-1 | ac | 6 | 65 | 76 | 493 |
| FW1NBNE02EX29A-1 | ac | 7 | 3 | 16 | 484 |
| FW1NBNE02D5X6J-1 | ac | 6 | 329 | 340 | 387 |
| FW1NBNE02D74IV-1 | ac | 5 | 208 | 217 | 499 |
| FW1NBNE02DT559-1 | ac | 6 | 2 | 13 | 385 |
| FW1NBNE02DYVQC-1 | ac | 8 | 268 | 283 | 309 |
| FW1NBNE02EMCQD-1 | ac | 8 | 88 | 103 | 195 |
| FW1NBNE02D62E6-1 | ac | 5 | 99 | 108 | 372 |
| FW1NBNE02ESJJB-1 | ac | 22 | 8 | 51 | 397 |
| FW1NBNE02DAGV4-1 | ac | 18 | 3 | 38 | 70 |
| FW1NBNE02EVLUZ-2 | aca | 5 | 161 | 175 | 391 |
| contig10228-1 | aca | 10 | 191 | 220 | 855 |
| contig13050-1 | aca | 6 | 146 | 163 | 291 |
| contig00518-1 | acc | 5 | 1184 | 1198 | 1240 |
| contig01733-1 | acc | 6 | 261 | 278 | 901 |
| contig03696-1 | acc | 5 | 762 | 776 | 897 |
| FW1NBNE02C3HR0-1 | acc | 5 | 249 | 263 | 399 |
| FW1NBNE02DUOCC-1 | acc | 7 | 45 | 65 | 401 |
| contig12325-1 | accag | 5 | 90 | 114 | 463 |
| contig04041-1 | accg | 6 | 77 | 100 | 1027 |
| FW1NBNE02EUL5K-2 | acct | 7 | 92 | 119 | 145 |
| contig03413-1 | acct | 8 | 6 | 37 | 417 |
| contig00639-1 | acga | 6 | 1887 | 1910 | 2150 |
| FW1NBNE02DMBS2-2 | acgt | 6 | 115 | 138 | 423 |
| contig02152-1 | act | 8 | 181 | 204 | 1204 |
| contig02646-1 | act | 5 | 122 | 136 | 544 |
| contig06356-1 | act | 8 | 76 | 99 | 190 |
| contig06514-1 | act | 5 | 91 | 105 | 645 |
| FW1NBNE02DBVQ8-1 | act | 5 | 162 | 176 | 386 |
| FW1NBNE02CZBLT-3 | acta | 5 | 41 | 60 | 62 |
| FW1NBNE02EU0F4-1 | acta | 13 | 87 | 138 | 138 |
| contig05893-1 | actgcc | 6 | 556 | 591 | 1097 |
| contig01726-2 | ag | 7 | 1666 | 1679 | 1858 |
| contig02747-2 | ag | 11 | 1040 | 1061 | 1068 |
| contig02755-2 | ag | 6 | 33 | 44 | 455 |
| contig05049-2 | ag | 8 | 526 | 541 | 657 |
| contig05640-2 | ag | 5 | 1663 | 1672 | 1728 |
| contig06072-2 | ag | 5 | 1210 | 1219 | 1415 |
| contig06612-2 | ag | 6 | 529 | 540 | 599 |
| contig09021-2 | ag | 5 | 169 | 178 | 547 |
| contig09541-2 | ag | 6 | 276 | 287 | 333 |
| contig09692-2 | ag | 5 | 205 | 214 | 251 |
| contig09779-2 | ag | 5 | 140 | 149 | 184 |
| contig13595-2 | ag | 9 | 38 | 55 | 507 |
| FW1NBNE02EDMQ3-2 | ag | 5 | 382 | 391 | 406 |
| FW1NBNE02DX2FU-2 | ag | 6 | 233 | 244 | 593 |
| FW1NBNE02DDA1N-2 | ag | 6 | 385 | 396 | 402 |
| FW1NBNE02C4VKG-2 | ag | 6 | 64 | 75 | 430 |
| FW1NBNE02D3WR3-2 | ag | 6 | 28 | 39 | 53 |
| FW1NBNE02EJB48-2 | ag | 5 | 68 | 77 | 513 |
| FW1NBNE02DK4ZL-2 | ag | 5 | 43 | 52 | 99 |
| FW1NBNE02DQM8V-2 | ag | 6 | 179 | 190 | 191 |
| FW1NBNE02C0BJR-2 | ag | 9 | 97 | 114 | 120 |
| FW1NBNE02D5BJ0-2 | ag | 9 | 414 | 431 | 434 |
| FW1NBNE02EL5XE-2 | ag | 8 | 42 | 57 | 157 |
| FW1NBNE02DLIZ2-2 | ag | 5 | 61 | 70 | 499 |
| FW1NBNE02DXPPT-2 | ag | 11 | 93 | 114 | 157 |
| FW1NBNE02DI32B-2 | ag | 7 | 471 | 484 | 496 |
| FW1NBNE02EFJYD-2 | ag | 6 | 34 | 45 | 388 |
| FW1NBNE02D71AZ-2 | ag | 5 | 47 | 56 | 184 |
| FW1NBNE02DFLW0-2 | ag | 16 | 125 | 156 | 159 |
| contig01726-3 | ag | 6 | 1826 | 1837 | 1858 |
| FW1NBNE02D1Z3L-3 | ag | 6 | 283 | 294 | 297 |
| FW1NBNE02DCA9P-3 | ag | 5 | 360 | 369 | 384 |
| FW1NBNE02EJYAI-3 | ag | 6 | 41 | 52 | 90 |
| contig04423-4 | ag | 5 | 799 | 808 | 813 |
| contig00004-1 | ag | 5 | 735 | 744 | 1515 |
| contig00041-1 | ag | 6 | 55 | 66 | 458 |
| contig00173-1 | ag | 5 | 44 | 53 | 1425 |
| contig00194-1 | ag | 6 | 65 | 76 | 2064 |
| contig00217-1 | ag | 8 | 53 | 68 | 186 |
| contig00273-1 | ag | 6 | 267 | 278 | 402 |
| contig00310-1 | ag | 5 | 2453 | 2462 | 2606 |
| contig00320-1 | ag | 6 | 291 | 302 | 490 |
| contig00418-1 | ag | 5 | 2 | 11 | 545 |
| contig00424-1 | ag | 6 | 506 | 517 | 689 |
| contig00454-1 | ag | 5 | 1531 | 1540 | 1579 |
| contig00541-1 | ag | 5 | 511 | 520 | 666 |
| contig00646-1 | ag | 8 | 64 | 79 | 883 |
| contig00707-1 | ag | 5 | 1396 | 1405 | 1498 |
| contig00736-1 | ag | 5 | 159 | 168 | 1479 |
| contig00780-1 | ag | 5 | 690 | 699 | 719 |
| contig00881-1 | ag | 13 | 1 | 26 | 126 |
| contig00913-1 | ag | 5 | 1453 | 1462 | 1510 |
| contig00942-1 | ag | 5 | 1721 | 1730 | 1787 |
| contig00966-1 | ag | 6 | 1364 | 1375 | 1442 |
| contig01329-1 | ag | 7 | 692 | 705 | 730 |
| contig01453-1 | ag | 6 | 942 | 953 | 1004 |
| contig01497-1 | ag | 6 | 246 | 257 | 1506 |
| contig01522-1 | ag | 5 | 521 | 530 | 547 |
| contig01570-1 | ag | 5 | 1088 | 1097 | 1152 |
| contig01634-1 | ag | 7 | 411 | 424 | 485 |
| contig01638-1 | ag | 5 | 755 | 764 | 1638 |
| contig01726-1 | ag | 5 | 1104 | 1113 | 1858 |
| contig01784-1 | ag | 5 | 194 | 203 | 746 |
| contig01946-1 | ag | 7 | 48 | 61 | 2055 |
| contig02176-1 | ag | 6 | 763 | 774 | 792 |
| contig02317-1 | ag | 9 | 386 | 403 | 595 |
| contig02532-1 | ag | 8 | 167 | 182 | 443 |
| contig02764-1 | ag | 8 | 1284 | 1299 | 1330 |
| contig02784-1 | ag | 11 | 793 | 814 | 846 |
| contig02822-1 | ag | 5 | 374 | 383 | 481 |
| contig02927-1 | ag | 5 | 682 | 691 | 743 |
| contig03117-1 | ag | 7 | 31 | 44 | 3025 |
| contig03233-1 | ag | 5 | 99 | 108 | 426 |
| contig03278-1 | ag | 8 | 1664 | 1679 | 1748 |
| contig03380-1 | ag | 5 | 306 | 315 | 533 |
| contig03531-1 | ag | 5 | 1268 | 1277 | 1295 |
| contig03573-1 | ag | 5 | 349 | 358 | 596 |
| contig03928-1 | ag | 6 | 1430 | 1441 | 1495 |
| contig03934-1 | ag | 6 | 2558 | 2569 | 2623 |
| contig03981-1 | ag | 5 | 8 | 17 | 1198 |
| contig03986-1 | ag | 5 | 26 | 35 | 207 |
| contig04074-1 | ag | 5 | 464 | 473 | 531 |
| contig04477-1 | ag | 5 | 422 | 431 | 638 |
| contig04532-1 | ag | 6 | 81 | 92 | 304 |
| contig04534-1 | ag | 6 | 82 | 93 | 228 |
| contig04750-1 | ag | 5 | 529 | 538 | 539 |
| contig04813-1 | ag | 5 | 1312 | 1321 | 1344 |
| contig04873-1 | ag | 5 | 619 | 628 | 864 |
| contig04892-1 | ag | 5 | 24 | 33 | 356 |
| contig04941-1 | ag | 5 | 443 | 452 | 481 |
| contig05029-1 | ag | 5 | 474 | 483 | 486 |
| contig05119-1 | ag | 5 | 6 | 15 | 717 |
| contig05179-1 | ag | 8 | 13 | 28 | 241 |
| contig05376-1 | ag | 8 | 394 | 409 | 540 |
| contig05672-1 | ag | 7 | 7 | 20 | 208 |
| contig05692-1 | ag | 5 | 230 | 239 | 1095 |
| contig06139-1 | ag | 5 | 480 | 489 | 552 |
| contig06240-1 | ag | 5 | 415 | 424 | 502 |
| contig06292-1 | ag | 5 | 39 | 48 | 403 |
| contig06301-1 | ag | 5 | 33 | 42 | 440 |
| contig06617-1 | ag | 6 | 50 | 61 | 1068 |
| contig06627-1 | ag | 5 | 62 | 71 | 124 |
| contig06686-1 | ag | 6 | 95 | 106 | 508 |
| contig06835-1 | ag | 5 | 54 | 63 | 544 |
| contig06904-1 | ag | 5 | 839 | 848 | 1414 |
| contig06980-1 | ag | 9 | 787 | 804 | 805 |
| contig07028-1 | ag | 5 | 427 | 436 | 457 |
| contig07029-1 | ag | 7 | 524 | 537 | 539 |
| contig07292-1 | ag | 6 | 654 | 665 | 712 |
| contig07451-1 | ag | 7 | 665 | 678 | 742 |
| contig07492-1 | ag | 5 | 548 | 557 | 1427 |
| contig07961-1 | ag | 5 | 603 | 612 | 692 |
| contig08056-1 | ag | 5 | 419 | 428 | 467 |
| contig08242-1 | ag | 7 | 4 | 17 | 1144 |
| contig08473-1 | ag | 5 | 659 | 668 | 739 |
| contig08570-1 | ag | 5 | 537 | 546 | 641 |
| contig08963-1 | ag | 5 | 1076 | 1085 | 1154 |
| contig09516-1 | ag | 5 | 445 | 454 | 496 |
| contig09531-1 | ag | 5 | 508 | 517 | 581 |
| contig09730-1 | ag | 5 | 593 | 602 | 660 |
| contig09779-1 | ag | 7 | 123 | 136 | 184 |
| contig09816-1 | ag | 13 | 411 | 436 | 436 |
| contig10097-1 | ag | 5 | 409 | 418 | 585 |
| contig10100-1 | ag | 5 | 205 | 214 | 780 |
| contig10304-1 | ag | 7 | 47 | 60 | 464 |
| contig10349-1 | ag | 5 | 66 | 75 | 494 |
| contig10806-1 | ag | 5 | 292 | 301 | 475 |
| contig10865-1 | ag | 5 | 438 | 447 | 463 |
| contig11224-1 | ag | 5 | 404 | 413 | 488 |
| contig12030-1 | ag | 5 | 247 | 256 | 428 |
| contig12035-1 | ag | 8 | 1 | 16 | 253 |
| contig12046-1 | ag | 5 | 70 | 79 | 498 |
| contig12098-1 | ag | 5 | 80 | 89 | 414 |
| contig12166-1 | ag | 5 | 310 | 319 | 416 |
| contig12297-1 | ag | 9 | 425 | 442 | 461 |
| contig12335-1 | ag | 5 | 673 | 682 | 781 |
| contig12460-1 | ag | 9 | 32 | 49 | 464 |
| contig12491-1 | ag | 6 | 525 | 536 | 544 |
| contig12548-1 | ag | 6 | 447 | 458 | 738 |
| contig12619-1 | ag | 11 | 120 | 141 | 285 |
| contig12651-1 | ag | 11 | 329 | 350 | 449 |
| contig12652-1 | ag | 9 | 32 | 49 | 412 |
| contig12829-1 | ag | 7 | 21 | 34 | 458 |
| contig12831-1 | ag | 5 | 44 | 53 | 442 |
| contig12951-1 | ag | 5 | 576 | 585 | 587 |
| contig12990-1 | ag | 5 | 41 | 50 | 397 |
| contig13230-1 | ag | 5 | 1 | 10 | 241 |
| contig13250-1 | ag | 7 | 29 | 42 | 257 |
| contig13363-1 | ag | 5 | 285 | 294 | 2734 |
| contig13369-1 | ag | 5 | 694 | 703 | 746 |
| contig13559-1 | ag | 11 | 52 | 73 | 159 |
| contig13569-1 | ag | 5 | 29 | 38 | 215 |
| contig13595-1 | ag | 6 | 1 | 12 | 507 |
| contig13633-1 | ag | 5 | 145 | 154 | 165 |
| contig13722-1 | ag | 8 | 1 | 16 | 148 |
| contig13878-1 | ag | 5 | 136 | 145 | 225 |
| contig13936-1 | ag | 9 | 41 | 58 | 335 |
| FW1NBNE02DU9TC-1 | ag | 5 | 196 | 205 | 433 |
| FW1NBNE02EEW7T-1 | ag | 5 | 192 | 201 | 479 |
| FW1NBNE02EBQAX-1 | ag | 5 | 291 | 300 | 371 |
| FW1NBNE02C2XKH-1 | ag | 5 | 118 | 127 | 479 |
| FW1NBNE02DB1WL-1 | ag | 6 | 69 | 80 | 263 |
| FW1NBNE02DO1FC-1 | ag | 6 | 379 | 390 | 445 |
| FW1NBNE02DMO0H-1 | ag | 5 | 463 | 472 | 521 |
| FW1NBNE02D4IVZ-1 | ag | 5 | 78 | 87 | 379 |
| FW1NBNE02DRYHR-1 | ag | 7 | 203 | 216 | 484 |
| FW1NBNE02C7XV7-1 | ag | 7 | 482 | 495 | 517 |
| FW1NBNE02C7HP1-1 | ag | 6 | 470 | 481 | 521 |
| FW1NBNE02EU7LA-1 | ag | 5 | 213 | 222 | 554 |
| FW1NBNE02DYL29-1 | ag | 5 | 331 | 340 | 474 |
| FW1NBNE02D6OLP-1 | ag | 6 | 402 | 413 | 517 |
| FW1NBNE02C4I7D-1 | ag | 11 | 363 | 384 | 389 |
| FW1NBNE02C7EX8-1 | ag | 11 | 35 | 56 | 65 |
| FW1NBNE02D5J7F-1 | ag | 5 | 11 | 20 | 516 |
| FW1NBNE02DW7OG-1 | ag | 13 | 11 | 36 | 51 |
| FW1NBNE02ELF21-1 | ag | 9 | 40 | 57 | 182 |
| FW1NBNE02DCA9P-1 | ag | 5 | 321 | 330 | 384 |
| FW1NBNE02DGQW6-1 | ag | 5 | 55 | 64 | 449 |
| FW1NBNE02EBZXI-1 | ag | 12 | 49 | 72 | 94 |
| FW1NBNE02DCC54-1 | ag | 10 | 476 | 495 | 496 |
| FW1NBNE02D62BK-1 | ag | 5 | 176 | 185 | 439 |
| FW1NBNE02EQ0JR-1 | ag | 16 | 348 | 379 | 402 |
| FW1NBNE02EFMT6-1 | ag | 5 | 381 | 390 | 435 |
| FW1NBNE02C5IQZ-1 | ag | 6 | 50 | 61 | 127 |
| FW1NBNE02DHKGX-1 | ag | 5 | 343 | 352 | 362 |
| FW1NBNE02EFHOZ-1 | ag | 7 | 467 | 480 | 489 |
| FW1NBNE02D4RYA-1 | ag | 6 | 578 | 589 | 593 |
| FW1NBNE02DSR4L-1 | ag | 7 | 374 | 387 | 439 |
| FW1NBNE02DDE4S-1 | ag | 7 | 167 | 180 | 490 |
| FW1NBNE02DK4ZL-1 | ag | 7 | 17 | 30 | 99 |
| FW1NBNE02C9CON-1 | ag | 5 | 497 | 506 | 528 |
| FW1NBNE02DTPU5-1 | ag | 7 | 6 | 19 | 431 |
| FW1NBNE02D2XYX-1 | ag | 8 | 244 | 259 | 428 |
| FW1NBNE02EYH3T-1 | ag | 9 | 324 | 341 | 356 |
| FW1NBNE02EN2FQ-1 | ag | 5 | 158 | 167 | 517 |
| FW1NBNE02D6CDR-1 | ag | 5 | 10 | 19 | 63 |
| FW1NBNE02DIQLQ-1 | ag | 10 | 143 | 162 | 251 |
| FW1NBNE02DDGDB-1 | ag | 5 | 28 | 37 | 198 |
| FW1NBNE02DXR8F-1 | ag | 7 | 37 | 50 | 116 |
| FW1NBNE02C302A-1 | ag | 6 | 49 | 60 | 92 |
| FW1NBNE02DVQ2Y-1 | ag | 5 | 341 | 350 | 367 |
| FW1NBNE02C5GNS-1 | ag | 5 | 12 | 21 | 431 |
| FW1NBNE02D28SA-1 | ag | 5 | 497 | 506 | 520 |
| FW1NBNE02DLIZ2-1 | ag | 6 | 24 | 35 | 499 |
| FW1NBNE02C9ZIT-1 | ag | 6 | 65 | 76 | 373 |
| FW1NBNE02EYDI5-1 | ag | 5 | 17 | 26 | 497 |
| FW1NBNE02DV9UC-1 | ag | 7 | 82 | 95 | 477 |
| FW1NBNE02EPBYF-1 | ag | 9 | 70 | 87 | 118 |
| FW1NBNE02EK9GH-1 | ag | 5 | 386 | 395 | 553 |
| FW1NBNE02DCSSP-1 | ag | 5 | 36 | 45 | 175 |
| FW1NBNE02DP8EW-1 | ag | 5 | 93 | 102 | 273 |
| FW1NBNE02DLS1C-1 | ag | 5 | 179 | 188 | 532 |
| FW1NBNE02EEJL1-1 | ag | 5 | 11 | 20 | 499 |
| FW1NBNE02D684V-1 | ag | 5 | 404 | 413 | 502 |
| FW1NBNE02D2TMO-1 | ag | 5 | 59 | 68 | 451 |
| FW1NBNE02D0AJO-1 | ag | 5 | 512 | 521 | 521 |
| FW1NBNE02D6YH7-1 | ag | 5 | 20 | 29 | 466 |
| FW1NBNE02C7Y90-1 | ag | 6 | 35 | 46 | 449 |
| FW1NBNE02D1E4S-1 | ag | 6 | 385 | 396 | 420 |
| FW1NBNE02EKEAY-1 | ag | 8 | 8 | 23 | 64 |
| FW1NBNE02CZXYG-1 | ag | 9 | 156 | 173 | 195 |
| FW1NBNE02DLSB6-1 | ag | 5 | 56 | 65 | 427 |
| FW1NBNE02ECN74-1 | ag | 5 | 388 | 397 | 484 |
| FW1NBNE02DFS7D-1 | ag | 5 | 272 | 281 | 528 |
| FW1NBNE02EW5MQ-1 | ag | 5 | 12 | 21 | 410 |
| FW1NBNE02EU33A-1 | ag | 5 | 238 | 247 | 509 |
| FW1NBNE02ETMHZ-1 | ag | 10 | 419 | 438 | 445 |
| FW1NBNE02D9CR2-1 | ag | 5 | 37 | 46 | 420 |
| FW1NBNE02C2HX4-1 | ag | 5 | 51 | 60 | 204 |
| FW1NBNE02D71AZ-1 | ag | 7 | 21 | 34 | 184 |
| FW1NBNE02C74OL-1 | ag | 5 | 204 | 213 | 467 |
| FW1NBNE02DAYHQ-1 | ag | 5 | 461 | 470 | 475 |
| FW1NBNE02DAOFI-1 | ag | 8 | 12 | 27 | 319 |
| FW1NBNE02D5BIH-1 | ag | 5 | 1 | 10 | 266 |
| FW1NBNE02EEKL9-1 | ag | 7 | 498 | 511 | 543 |
| FW1NBNE02DFW7P-1 | ag | 5 | 45 | 54 | 502 |
| FW1NBNE02DOXNM-1 | ag | 5 | 1 | 10 | 415 |
| FW1NBNE02C9HRY-1 | ag | 5 | 378 | 387 | 411 |
| FW1NBNE02C1SL4-1 | ag | 5 | 349 | 358 | 421 |
| FW1NBNE02D4IZ9-1 | ag | 5 | 57 | 66 | 389 |
| FW1NBNE02D09CC-1 | ag | 6 | 17 | 28 | 438 |
| FW1NBNE02DLY50-1 | ag | 5 | 94 | 103 | 239 |
| FW1NBNE02DR7MD-1 | ag | 5 | 431 | 440 | 479 |
| FW1NBNE02C1PVH-1 | ag | 6 | 86 | 97 | 247 |
| FW1NBNE02ENUO5-1 | ag | 8 | 92 | 107 | 409 |
| FW1NBNE02EZQBG-1 | ag | 9 | 380 | 397 | 397 |
| FW1NBNE02C9YO8-1 | ag | 10 | 25 | 44 | 152 |
| FW1NBNE02C08C2-1 | ag | 6 | 72 | 83 | 195 |
| FW1NBNE02EPFFF-1 | ag | 6 | 29 | 40 | 100 |
| FW1NBNE02DT9GE-1 | ag | 12 | 49 | 72 | 86 |
| FW1NBNE02C4L5U-1 | ag | 5 | 84 | 93 | 112 |
| FW1NBNE02EYNHX-1 | ag | 6 | 69 | 80 | 212 |
| FW1NBNE02D9OWE-1 | ag | 7 | 264 | 277 | 359 |
| contig06409-2 | aga | 5 | 272 | 286 | 673 |
| contig00474-3 | aga | 5 | 605 | 619 | 1207 |
| contig01320-1 | aga | 6 | 17 | 34 | 1555 |
| contig04083-1 | aga | 8 | 617 | 640 | 668 |
| contig04590-1 | aga | 6 | 218 | 235 | 729 |
| contig04886-1 | aga | 11 | 1 | 33 | 788 |
| contig06002-1 | aga | 6 | 548 | 565 | 664 |
| contig07951-1 | aga | 6 | 101 | 118 | 255 |
| contig09117-1 | aga | 6 | 348 | 365 | 435 |
| contig10698-1 | aga | 6 | 59 | 76 | 491 |
| contig13337-1 | aga | 6 | 14 | 31 | 856 |
| FW1NBNE02C2HJO-1 | aga | 5 | 295 | 309 | 368 |
| FW1NBNE02C1PDY-1 | aga | 6 | 222 | 239 | 250 |
| FW1NBNE02DD7HS-1 | aga | 5 | 209 | 223 | 535 |
| FW1NBNE02EG8YH-1 | aga | 5 | 17 | 31 | 456 |
| FW1NBNE02ES9WF-1 | aga | 5 | 31 | 45 | 477 |
| FW1NBNE02DSJ1N-1 | aga | 5 | 61 | 75 | 471 |
| FW1NBNE02DNOSD-1 | aga | 5 | 303 | 317 | 361 |
| FW1NBNE02DKDTY-1 | agac | 7 | 52 | 79 | 475 |
| FW1NBNE02DEV60-2 | agagc | 5 | 17 | 41 | 82 |
| FW1NBNE02EERBX-1 | agagg | 5 | 198 | 222 | 310 |
| contig11754-2 | agat | 7 | 35 | 62 | 472 |
| contig02194-1 | agat | 5 | 72 | 91 | 2219 |
| contig03680-2 | agc | 5 | 435 | 449 | 1060 |
| contig06891-2 | agc | 5 | 251 | 265 | 345 |
| FW1NBNE02DTPU5-2 | agc | 5 | 279 | 293 | 431 |
| contig01876-1 | agc | 8 | 350 | 373 | 1353 |
| contig02252-1 | agc | 5 | 103 | 117 | 280 |
| contig02293-1 | agc | 6 | 74 | 91 | 960 |
| contig02879-1 | agc | 5 | 320 | 334 | 1231 |
| contig03596-1 | agc | 6 | 569 | 586 | 711 |
| contig04803-1 | agc | 7 | 66 | 86 | 1714 |
| contig05667-1 | agc | 9 | 252 | 278 | 1707 |
| contig06403-1 | agc | 6 | 200 | 217 | 442 |
| contig10803-1 | agc | 6 | 322 | 339 | 350 |
| contig11814-1 | agc | 5 | 446 | 460 | 477 |
| contig12120-1 | agc | 6 | 329 | 346 | 476 |
| contig12782-1 | agc | 7 | 175 | 195 | 485 |
| FW1NBNE02C4GIZ-1 | agc | 6 | 374 | 391 | 494 |
| FW1NBNE02EJXZ6-1 | agc | 5 | 37 | 51 | 400 |
| FW1NBNE02D4QH2-1 | agc | 5 | 2 | 16 | 455 |
| FW1NBNE02ER7IK-1 | agc | 5 | 74 | 88 | 201 |
| FW1NBNE02D6QMF-1 | agc | 6 | 237 | 254 | 422 |
| FW1NBNE02EH6N9-1 | agc | 7 | 40 | 60 | 226 |
| FW1NBNE02C5RZ4-1 | agc | 6 | 111 | 128 | 396 |
| contig03589-1 | agcacc | 5 | 195 | 224 | 595 |
| FW1NBNE02DHZE1-1 | agct | 5 | 23 | 42 | 497 |
| FW1NBNE02EU32P-2 | agcta | 6 | 330 | 359 | 488 |
| contig01557-2 | agg | 6 | 230 | 247 | 549 |
| contig00148-1 | agg | 5 | 95 | 109 | 658 |
| contig00559-1 | agg | 5 | 319 | 333 | 702 |
| contig00683-1 | agg | 6 | 440 | 457 | 1046 |
| contig02589-1 | agg | 5 | 1330 | 1344 | 1465 |
| contig03562-1 | agg | 7 | 406 | 426 | 461 |
| contig12125-1 | agg | 5 | 292 | 306 | 317 |
| contig12886-1 | agg | 5 | 328 | 342 | 430 |
| FW1NBNE02DAB4V-1 | agg | 5 | 45 | 59 | 528 |
| contig07492-2 | aggc | 6 | 96 | 119 | 1427 |
| contig11676-1 | aggc | 7 | 238 | 265 | 655 |
| contig00963-1 | aggga | 5 | 158 | 182 | 1238 |
| contig03569-1 | agt | 5 | 212 | 226 | 397 |
| FW1NBNE02C0QQL-1 | agt | 6 | 7 | 24 | 494 |
| contig00118-2 | at | 5 | 488 | 497 | 621 |
| contig03817-2 | at | 5 | 636 | 645 | 686 |
| contig08758-2 | at | 5 | 346 | 355 | 368 |
| contig10252-2 | at | 8 | 286 | 301 | 492 |
| contig10629-2 | at | 5 | 67 | 76 | 247 |
| contig12098-2 | at | 5 | 121 | 130 | 414 |
| contig13212-2 | at | 11 | 99 | 120 | 269 |
| contig13249-2 | at | 31 | 173 | 234 | 235 |
| contig13694-2 | at | 23 | 241 | 286 | 286 |
| FW1NBNE02C2XN4-2 | at | 6 | 125 | 136 | 257 |
| FW1NBNE02C6YQL-2 | at | 19 | 83 | 120 | 121 |
| FW1NBNE02DPZ1P-2 | at | 15 | 56 | 85 | 85 |
| FW1NBNE02C2XKH-2 | at | 5 | 374 | 383 | 479 |
| FW1NBNE02C9FZT-2 | at | 7 | 191 | 204 | 387 |
| FW1NBNE02D5RPG-2 | at | 5 | 77 | 86 | 483 |
| FW1NBNE02DMN18-2 | at | 11 | 58 | 79 | 221 |
| FW1NBNE02D0438-2 | at | 7 | 154 | 167 | 306 |
| FW1NBNE02DD658-2 | at | 10 | 60 | 79 | 96 |
| FW1NBNE02D3WL6-2 | at | 164 | 68 | 395 | 396 |
| FW1NBNE02DCA9P-2 | at | 8 | 344 | 359 | 384 |
| FW1NBNE02EM67O-2 | at | 19 | 203 | 240 | 258 |
| FW1NBNE02ESZ0O-2 | at | 8 | 56 | 71 | 72 |
| FW1NBNE02EWJLQ-2 | at | 8 | 445 | 460 | 475 |
| FW1NBNE02D7A6S-2 | at | 7 | 232 | 245 | 293 |
| FW1NBNE02DSN6P-2 | at | 6 | 265 | 276 | 359 |
| FW1NBNE02D7AYG-2 | at | 18 | 31 | 66 | 66 |
| FW1NBNE02DQK2F-2 | at | 11 | 58 | 79 | 211 |
| FW1NBNE02DBI6A-2 | at | 10 | 58 | 77 | 79 |
| FW1NBNE02DL98R-2 | at | 17 | 371 | 404 | 404 |
| FW1NBNE02ESJJB-2 | at | 158 | 54 | 369 | 397 |
| FW1NBNE02DAGV4-2 | at | 12 | 47 | 70 | 70 |
| FW1NBNE02D5RPG-3 | at | 5 | 244 | 253 | 483 |
| FW1NBNE02C521M-3 | at | 14 | 163 | 190 | 224 |
| FW1NBNE02DTLAB-3 | at | 11 | 67 | 88 | 89 |
| FW1NBNE02EW71D-3 | at | 8 | 156 | 171 | 183 |
| FW1NBNE02EMCQD-3 | at | 13 | 161 | 186 | 195 |
| FW1NBNE02ESJJB-3 | at | 13 | 371 | 396 | 397 |
| contig12716-4 | at | 16 | 415 | 446 | 446 |
| FW1NBNE02EGFRF-5 | at | 6 | 287 | 298 | 349 |
| contig00007-1 | at | 5 | 6069 | 6078 | 10423 |
| contig00018-1 | at | 5 | 1823 | 1832 | 1964 |
| contig00091-1 | at | 6 | 1083 | 1094 | 1171 |
| contig00118-1 | at | 5 | 321 | 330 | 621 |
| contig00120-1 | at | 5 | 553 | 562 | 1202 |
| contig00445-1 | at | 10 | 5 | 24 | 1815 |
| contig00477-1 | at | 5 | 1617 | 1626 | 1645 |
| contig00508-1 | at | 9 | 390 | 407 | 1877 |
| contig00537-1 | at | 11 | 27 | 48 | 479 |
| contig00680-1 | at | 7 | 34 | 47 | 852 |
| contig00735-1 | at | 8 | 1348 | 1363 | 1542 |
| contig00783-1 | at | 16 | 1 | 32 | 825 |
| contig00797-1 | at | 5 | 83 | 92 | 165 |
| contig00850-1 | at | 5 | 843 | 852 | 938 |
| contig00902-1 | at | 13 | 220 | 245 | 1696 |
| contig01103-1 | at | 5 | 545 | 554 | 685 |
| contig01165-1 | at | 7 | 187 | 200 | 409 |
| contig01390-1 | at | 11 | 234 | 255 | 255 |
| contig01458-1 | at | 7 | 270 | 283 | 1029 |
| contig01832-1 | at | 7 | 1272 | 1285 | 1292 |
| contig01948-1 | at | 5 | 377 | 386 | 1315 |
| contig01973-1 | at | 5 | 253 | 262 | 2086 |
| contig02117-1 | at | 5 | 531 | 540 | 636 |
| contig02215-1 | at | 5 | 46 | 55 | 766 |
| contig02288-1 | at | 6 | 329 | 340 | 458 |
| contig02425-1 | at | 5 | 110 | 119 | 196 |
| contig02439-1 | at | 6 | 173 | 184 | 596 |
| contig02516-1 | at | 10 | 872 | 891 | 1025 |
| contig02598-1 | at | 8 | 996 | 1011 | 1090 |
| contig02675-1 | at | 5 | 315 | 324 | 465 |
| contig02707-1 | at | 5 | 142 | 151 | 492 |
| contig02881-1 | at | 5 | 1030 | 1039 | 2159 |
| contig02892-1 | at | 5 | 211 | 220 | 241 |
| contig02918-1 | at | 9 | 636 | 653 | 692 |
| contig02938-1 | at | 5 | 98 | 107 | 797 |
| contig03522-1 | at | 7 | 424 | 437 | 477 |
| contig03655-1 | at | 5 | 1834 | 1843 | 2000 |
| contig03680-1 | at | 15 | 1 | 30 | 1060 |
| contig03889-1 | at | 6 | 348 | 359 | 373 |
| contig03900-1 | at | 9 | 427 | 444 | 445 |
| contig04241-1 | at | 5 | 164 | 173 | 270 |
| contig04423-1 | at | 7 | 197 | 210 | 813 |
| contig04589-1 | at | 7 | 94 | 107 | 521 |
| contig04593-1 | at | 18 | 1658 | 1693 | 1693 |
| contig04684-1 | at | 7 | 92 | 105 | 1450 |
| contig04806-1 | at | 5 | 154 | 163 | 383 |
| contig05177-1 | at | 5 | 601 | 610 | 1169 |
| contig05636-1 | at | 7 | 16 | 29 | 465 |
| contig05696-1 | at | 5 | 158 | 167 | 306 |
| contig05799-1 | at | 5 | 394 | 403 | 494 |
| contig06077-1 | at | 6 | 402 | 413 | 487 |
| contig06409-1 | at | 10 | 452 | 471 | 673 |
| contig06418-1 | at | 9 | 644 | 661 | 947 |
| contig06427-1 | at | 8 | 229 | 244 | 1267 |
| contig06486-1 | at | 8 | 130 | 145 | 1547 |
| contig06525-1 | at | 5 | 46 | 55 | 424 |
| contig06612-1 | at | 5 | 519 | 528 | 599 |
| contig06771-1 | at | 5 | 89 | 98 | 672 |
| contig07022-1 | at | 5 | 45 | 54 | 1361 |
| contig07272-1 | at | 8 | 412 | 427 | 429 |
| contig07393-1 | at | 5 | 55 | 64 | 585 |
| contig07765-1 | at | 5 | 72 | 81 | 871 |
| contig07767-1 | at | 5 | 10 | 19 | 503 |
| contig08151-1 | at | 7 | 53 | 66 | 1626 |
| contig08172-1 | at | 5 | 111 | 120 | 812 |
| contig08191-1 | at | 6 | 8 | 19 | 105 |
| contig08212-1 | at | 5 | 188 | 197 | 1587 |
| contig08541-1 | at | 6 | 3 | 14 | 442 |
| contig08779-1 | at | 9 | 515 | 532 | 532 |
| contig09096-1 | at | 5 | 451 | 460 | 779 |
| contig09261-1 | at | 6 | 343 | 354 | 501 |
| contig09343-1 | at | 14 | 455 | 482 | 501 |
| contig09466-1 | at | 7 | 53 | 66 | 527 |
| contig09495-1 | at | 6 | 465 | 476 | 500 |
| contig10154-1 | at | 9 | 24 | 41 | 384 |
| contig10344-1 | at | 5 | 181 | 190 | 282 |
| contig10585-1 | at | 6 | 53 | 64 | 652 |
| contig12157-1 | at | 7 | 120 | 133 | 533 |
| contig12320-1 | at | 6 | 229 | 240 | 697 |
| contig12364-1 | at | 5 | 518 | 527 | 671 |
| contig12546-1 | at | 7 | 93 | 106 | 537 |
| contig12670-1 | at | 9 | 426 | 443 | 444 |
| contig12761-1 | at | 6 | 199 | 210 | 287 |
| contig12769-1 | at | 6 | 401 | 412 | 471 |
| contig12895-1 | at | 10 | 408 | 427 | 427 |
| contig12977-1 | at | 10 | 378 | 397 | 419 |
| contig13087-1 | at | 12 | 368 | 391 | 393 |
| contig13212-1 | at | 5 | 52 | 61 | 269 |
| contig13238-1 | at | 9 | 259 | 276 | 276 |
| contig13245-1 | at | 12 | 205 | 228 | 276 |
| contig13274-1 | at | 5 | 20 | 29 | 149 |
| contig13444-1 | at | 8 | 51 | 66 | 127 |
| contig13463-1 | at | 8 | 119 | 134 | 159 |
| contig13605-1 | at | 5 | 28 | 37 | 173 |
| contig13610-1 | at | 5 | 51 | 60 | 117 |
| contig13632-1 | at | 14 | 8 | 35 | 104 |
| contig13674-1 | at | 5 | 10 | 19 | 1074 |
| contig13772-1 | at | 5 | 106 | 115 | 483 |
| contig13793-1 | at | 5 | 668 | 677 | 726 |
| contig13919-1 | at | 12 | 82 | 105 | 749 |
| FW1NBNE02DZTHB-1 | at | 7 | 147 | 160 | 502 |
| FW1NBNE02ECEJ8-1 | at | 10 | 1 | 20 | 88 |
| FW1NBNE02C2XN4-1 | at | 6 | 102 | 113 | 257 |
| FW1NBNE02D15D7-1 | at | 12 | 35 | 58 | 59 |
| FW1NBNE02C7OV5-1 | at | 7 | 8 | 21 | 129 |
| FW1NBNE02C3UJ2-1 | at | 5 | 79 | 88 | 189 |
| FW1NBNE02D2K4F-1 | at | 7 | 310 | 323 | 486 |
| FW1NBNE02ECVEQ-1 | at | 14 | 25 | 52 | 54 |
| FW1NBNE02C03G4-1 | at | 9 | 6 | 23 | 543 |
| FW1NBNE02C261O-1 | at | 11 | 1 | 22 | 68 |
| FW1NBNE02D6UFH-1 | at | 9 | 29 | 46 | 188 |
| FW1NBNE02EW7S4-1 | at | 5 | 466 | 475 | 494 |
| FW1NBNE02EM9FK-1 | at | 5 | 60 | 69 | 501 |
| FW1NBNE02DNPOT-1 | at | 9 | 251 | 268 | 482 |
| FW1NBNE02DDJBN-1 | at | 22 | 144 | 187 | 196 |
| FW1NBNE02D8NWY-1 | at | 15 | 197 | 226 | 228 |
| FW1NBNE02DTJFW-1 | at | 5 | 201 | 210 | 551 |
| FW1NBNE02EPARP-1 | at | 5 | 130 | 139 | 185 |
| FW1NBNE02EW2KR-1 | at | 10 | 25 | 44 | 52 |
| FW1NBNE02DPDLP-1 | at | 5 | 14 | 23 | 512 |
| FW1NBNE02DCAT6-1 | at | 9 | 1 | 18 | 128 |
| FW1NBNE02DWK95-1 | at | 10 | 85 | 104 | 104 |
| FW1NBNE02DKP3I-1 | at | 5 | 506 | 515 | 579 |
| FW1NBNE02DIMKF-1 | at | 197 | 1 | 394 | 395 |
| FW1NBNE02D8KZW-1 | at | 11 | 43 | 64 | 65 |
| FW1NBNE02D4T5J-1 | at | 25 | 1 | 50 | 56 |
| FW1NBNE02DVYHQ-1 | at | 6 | 60 | 71 | 509 |
| FW1NBNE02D7WUD-1 | at | 11 | 195 | 216 | 488 |
| FW1NBNE02DEII4-1 | at | 5 | 424 | 433 | 449 |
| FW1NBNE02EFD8R-1 | at | 6 | 6 | 17 | 203 |
| FW1NBNE02EJKY1-1 | at | 5 | 78 | 87 | 172 |
| FW1NBNE02DXZ3L-1 | at | 197 | 1 | 394 | 395 |
| FW1NBNE02DIOCL-1 | at | 7 | 370 | 383 | 461 |
| FW1NBNE02DUKXW-1 | at | 7 | 360 | 373 | 513 |
| FW1NBNE02D2C78-1 | at | 8 | 267 | 282 | 492 |
| FW1NBNE02DMN18-1 | at | 5 | 34 | 43 | 221 |
| FW1NBNE02D8B59-1 | at | 12 | 35 | 58 | 67 |
| FW1NBNE02DX2FU-1 | at | 7 | 219 | 232 | 593 |
| FW1NBNE02D3DKV-1 | at | 6 | 16 | 27 | 345 |
| FW1NBNE02EAZWE-1 | at | 9 | 45 | 62 | 126 |
| FW1NBNE02DW2N1-1 | at | 9 | 38 | 55 | 57 |
| FW1NBNE02D2HZK-1 | at | 10 | 410 | 429 | 434 |
| FW1NBNE02DJZQR-1 | at | 6 | 164 | 175 | 474 |
| FW1NBNE02DO2Z0-1 | at | 6 | 427 | 438 | 450 |
| FW1NBNE02DVLU7-1 | at | 6 | 11 | 22 | 318 |
| FW1NBNE02DD658-1 | at | 5 | 36 | 45 | 96 |
| FW1NBNE02C4VKG-1 | at | 5 | 54 | 63 | 430 |
| FW1NBNE02EENVX-1 | at | 6 | 23 | 34 | 492 |
| FW1NBNE02D3WL6-1 | at | 33 | 1 | 66 | 396 |
| FW1NBNE02DYPFM-1 | at | 15 | 287 | 316 | 317 |
| FW1NBNE02D1Z3L-1 | at | 15 | 243 | 272 | 297 |
| FW1NBNE02EU24I-1 | at | 5 | 32 | 41 | 132 |
| FW1NBNE02C8HRX-1 | at | 6 | 31 | 42 | 417 |
| FW1NBNE02DO1SW-1 | at | 7 | 431 | 444 | 457 |
| FW1NBNE02D6S66-1 | at | 7 | 51 | 64 | 521 |
| FW1NBNE02DMGEH-1 | at | 9 | 497 | 514 | 550 |
| FW1NBNE02D3WR3-1 | at | 7 | 14 | 27 | 53 |
| FW1NBNE02EVER5-1 | at | 5 | 177 | 186 | 513 |
| FW1NBNE02DEXMV-1 | at | 7 | 468 | 481 | 533 |
| FW1NBNE02ED4PO-1 | at | 7 | 109 | 122 | 486 |
| FW1NBNE02DPL4E-1 | at | 9 | 332 | 349 | 400 |
| FW1NBNE02D8NDO-1 | at | 7 | 24 | 37 | 163 |
| FW1NBNE02DS5BA-1 | at | 11 | 127 | 148 | 182 |
| FW1NBNE02DUU8P-1 | at | 10 | 134 | 153 | 436 |
| FW1NBNE02D6EYM-1 | at | 11 | 419 | 440 | 441 |
| FW1NBNE02DBZJJ-1 | at | 5 | 108 | 117 | 482 |
| FW1NBNE02DRJHE-1 | at | 10 | 1 | 20 | 66 |
| FW1NBNE02EIOBE-1 | at | 7 | 12 | 25 | 533 |
| FW1NBNE02EB28J-1 | at | 5 | 36 | 45 | 66 |
| FW1NBNE02DU4RX-1 | at | 5 | 174 | 183 | 476 |
| FW1NBNE02D4KVD-1 | at | 11 | 365 | 386 | 469 |
| FW1NBNE02EG3NV-1 | at | 13 | 4 | 29 | 75 |
| FW1NBNE02DTZ39-1 | at | 12 | 107 | 130 | 144 |
| FW1NBNE02EMJJF-1 | at | 5 | 205 | 214 | 481 |
| FW1NBNE02EN0SF-1 | at | 6 | 59 | 70 | 509 |
| FW1NBNE02ENDMX-1 | at | 156 | 135 | 446 | 447 |
| FW1NBNE02EQP39-1 | at | 5 | 117 | 126 | 512 |
| FW1NBNE02EP28D-1 | at | 10 | 134 | 153 | 392 |
| FW1NBNE02DV8TL-1 | at | 11 | 68 | 89 | 95 |
| FW1NBNE02DCCXQ-1 | at | 7 | 32 | 45 | 101 |
| FW1NBNE02EHMK8-1 | at | 5 | 71 | 80 | 527 |
| FW1NBNE02DA2VN-1 | at | 26 | 1 | 52 | 61 |
| FW1NBNE02DIVR7-1 | at | 5 | 180 | 189 | 467 |
| FW1NBNE02C29CZ-1 | at | 9 | 231 | 248 | 369 |
| FW1NBNE02D5BJ0-1 | at | 10 | 394 | 413 | 434 |
| FW1NBNE02EVG91-1 | at | 5 | 7 | 16 | 458 |
| FW1NBNE02EP45J-1 | at | 9 | 414 | 431 | 436 |
| FW1NBNE02EPPWQ-1 | at | 12 | 74 | 97 | 171 |
| FW1NBNE02D1RKH-1 | at | 32 | 214 | 277 | 277 |
| FW1NBNE02C67ZF-1 | at | 21 | 150 | 191 | 196 |
| FW1NBNE02D5WPC-1 | at | 10 | 63 | 82 | 118 |
| FW1NBNE02DV77P-1 | at | 12 | 120 | 143 | 154 |
| FW1NBNE02D2W6Z-1 | at | 6 | 55 | 66 | 479 |
| FW1NBNE02EQLK7-1 | at | 5 | 505 | 514 | 536 |
| FW1NBNE02D50IR-1 | at | 6 | 44 | 55 | 396 |
| FW1NBNE02EXJZW-1 | at | 7 | 108 | 121 | 406 |
| FW1NBNE02EO0ZC-1 | at | 8 | 390 | 405 | 420 |
| FW1NBNE02DI32B-1 | at | 6 | 457 | 468 | 496 |
| FW1NBNE02DCDXP-1 | at | 15 | 413 | 442 | 442 |
| FW1NBNE02C1RX5-1 | at | 5 | 375 | 384 | 447 |
| FW1NBNE02DL92A-1 | at | 5 | 481 | 490 | 516 |
| FW1NBNE02EVEE4-1 | at | 10 | 52 | 71 | 143 |
| FW1NBNE02EJ9K8-1 | at | 5 | 53 | 62 | 62 |
| FW1NBNE02DG98L-1 | at | 12 | 38 | 61 | 65 |
| FW1NBNE02EVQAA-1 | at | 7 | 11 | 24 | 392 |
| FW1NBNE02DZ3CA-1 | at | 6 | 42 | 53 | 60 |
| FW1NBNE02D08QZ-1 | at | 11 | 99 | 120 | 265 |
| FW1NBNE02D0OLO-1 | at | 14 | 164 | 191 | 192 |
| FW1NBNE02EC41L-1 | at | 46 | 1 | 92 | 93 |
| FW1NBNE02EOVQB-1 | at | 9 | 394 | 411 | 412 |
| FW1NBNE02DPFJ0-1 | at | 8 | 38 | 53 | 58 |
| FW1NBNE02EYG8R-1 | at | 9 | 358 | 375 | 458 |
| FW1NBNE02DPIYR-1 | at | 5 | 18 | 27 | 495 |
| FW1NBNE02D9J1Y-1 | at | 11 | 90 | 111 | 178 |
| FW1NBNE02EG5F3-1 | at | 5 | 47 | 56 | 147 |
| FW1NBNE02DGDZD-1 | at | 13 | 134 | 159 | 310 |
| FW1NBNE02C9736-1 | at | 5 | 438 | 447 | 450 |
| FW1NBNE02EDY6D-1 | at | 7 | 224 | 237 | 434 |
| FW1NBNE02DVYO1-1 | at | 5 | 52 | 61 | 61 |
| FW1NBNE02C6SAN-1 | at | 5 | 88 | 97 | 111 |
| FW1NBNE02DD41O-1 | at | 66 | 1 | 132 | 133 |
| FW1NBNE02D7IY3-1 | at | 5 | 39 | 48 | 404 |
| FW1NBNE02EDDIT-1 | at | 7 | 30 | 43 | 79 |
| FW1NBNE02EJ99N-1 | at | 10 | 70 | 89 | 190 |
| FW1NBNE02D3LJU-1 | at | 10 | 54 | 73 | 79 |
| FW1NBNE02EBJDM-1 | at | 8 | 435 | 450 | 451 |
| FW1NBNE02EBZYY-1 | at | 8 | 78 | 93 | 450 |
| FW1NBNE02DFCRP-1 | at | 6 | 53 | 64 | 125 |
| FW1NBNE02CZOSL-1 | at | 6 | 32 | 43 | 177 |
| FW1NBNE02DH4WP-1 | at | 10 | 303 | 322 | 348 |
| FW1NBNE02EWNT4-1 | at | 5 | 41 | 50 | 432 |
| FW1NBNE02EMEHI-1 | at | 6 | 211 | 222 | 294 |
| FW1NBNE02DD3PD-1 | at | 5 | 130 | 139 | 451 |
| FW1NBNE02C3KQJ-1 | at | 5 | 131 | 140 | 344 |
| FW1NBNE02DZ3BG-1 | at | 9 | 20 | 37 | 112 |
| FW1NBNE02ENQEQ-1 | at | 6 | 120 | 131 | 208 |
| FW1NBNE02EFEHR-1 | at | 5 | 50 | 59 | 59 |
| FW1NBNE02DVEVF-1 | at | 5 | 164 | 173 | 341 |
| FW1NBNE02DSO8U-1 | at | 6 | 35 | 46 | 241 |
| FW1NBNE02DMBS2-1 | at | 5 | 296 | 305 | 423 |
| FW1NBNE02EHPRT-1 | at | 45 | 1 | 90 | 91 |
| FW1NBNE02DQK2F-1 | at | 5 | 34 | 43 | 211 |
| FW1NBNE02EC3S6-1 | at | 10 | 171 | 190 | 191 |
| FW1NBNE02C0NQL-1 | at | 6 | 352 | 363 | 446 |
| FW1NBNE02C571U-1 | at | 6 | 40 | 51 | 52 |
| FW1NBNE02DP1RS-1 | at | 8 | 143 | 158 | 183 |
| FW1NBNE02DFLW0-1 | at | 9 | 107 | 124 | 159 |
| FW1NBNE02DBZ6W-1 | at | 7 | 46 | 59 | 85 |
| FW1NBNE02C7K2J-1 | at | 5 | 107 | 116 | 237 |
| FW1NBNE02CZW0O-1 | at | 6 | 499 | 510 | 511 |
| FW1NBNE02DKPKP-1 | at | 12 | 191 | 214 | 217 |
| FW1NBNE02EIRPI-1 | at | 5 | 172 | 181 | 323 |
| FW1NBNE02DV9MY-1 | at | 12 | 153 | 176 | 177 |
| FW1NBNE02EIXAW-1 | at | 5 | 269 | 278 | 355 |
| FW1NBNE02D892Y-1 | at | 5 | 443 | 452 | 508 |
| FW1NBNE02D85XI-2 | ata | 5 | 248 | 262 | 517 |
| FW1NBNE02ESZ0O-3 | ata | 9 | 32 | 58 | 72 |
| contig00412-1 | ata | 6 | 253 | 270 | 423 |
| contig00497-1 | ata | 7 | 78 | 98 | 1014 |
| contig01582-1 | ata | 7 | 95 | 115 | 366 |
| contig02917-1 | ata | 8 | 61 | 84 | 691 |
| contig04650-1 | ata | 12 | 178 | 213 | 533 |
| contig10320-1 | ata | 7 | 16 | 36 | 619 |
| contig11943-1 | ata | 5 | 58 | 72 | 548 |
| contig12108-1 | ata | 5 | 53 | 67 | 175 |
| contig13565-1 | ata | 5 | 122 | 136 | 676 |
| FW1NBNE02D7N46-1 | ata | 6 | 56 | 73 | 395 |
| FW1NBNE02DROB9-1 | ata | 8 | 308 | 331 | 533 |
| contig11288-1 | atac | 5 | 266 | 285 | 380 |
| FW1NBNE02D4BKY-1 | atac | 5 | 77 | 96 | 398 |
| FW1NBNE02DPKN1-1 | atatag | 5 | 396 | 425 | 483 |
| contig04608-2 | atc | 5 | 850 | 864 | 1253 |
| FW1NBNE02DZIH5-2 | atc | 7 | 200 | 220 | 391 |
| contig04506-3 | atc | 5 | 106 | 120 | 2261 |
| contig10945-3 | atc | 5 | 227 | 241 | 556 |
| contig00082-1 | atc | 7 | 125 | 145 | 2034 |
| contig00853-1 | atc | 8 | 296 | 319 | 1530 |
| contig03043-1 | atc | 7 | 15 | 35 | 372 |
| contig09725-1 | atc | 5 | 382 | 396 | 433 |
| contig11471-1 | atc | 5 | 2 | 16 | 299 |
| FW1NBNE02DGX2V-1 | atc | 5 | 70 | 84 | 509 |
| FW1NBNE02DF51W-1 | atc | 6 | 224 | 241 | 473 |
| FW1NBNE02DUFYM-1 | atc | 6 | 270 | 287 | 334 |
| FW1NBNE02EV6RX-1 | atc | 6 | 258 | 275 | 444 |
| FW1NBNE02D2LU9-1 | atccc | 6 | 2 | 31 | 337 |
| contig07021-1 | atct | 5 | 81 | 100 | 121 |
| contig06301-2 | atg | 5 | 315 | 329 | 440 |
| FW1NBNE02D09CC-2 | atg | 5 | 375 | 389 | 438 |
| contig04273-1 | atg | 6 | 164 | 181 | 364 |
| contig05434-1 | atg | 7 | 180 | 200 | 307 |
| contig11001-1 | atg | 6 | 638 | 655 | 670 |
| contig12406-1 | atg | 5 | 66 | 80 | 344 |
| contig12644-1 | atg | 5 | 26 | 40 | 367 |
| FW1NBNE02EL23I-1 | atg | 5 | 172 | 186 | 524 |
| FW1NBNE02EBFR1-1 | atg | 5 | 171 | 185 | 485 |
| FW1NBNE02D1SXB-1 | atg | 8 | 249 | 272 | 400 |
| FW1NBNE02DRZMS-1 | atg | 6 | 229 | 246 | 461 |
| FW1NBNE02DZ152-2 | atgc | 5 | 252 | 271 | 505 |
| contig05375-2 | atgg | 5 | 1087 | 1106 | 1130 |
| contig11314-1 | atggag | 5 | 951 | 980 | 1011 |
| contig00491-1 | atgt | 5 | 836 | 855 | 971 |
| FW1NBNE02EOVQB-2 | att | 7 | 14 | 34 | 412 |
| contig01167-1 | att | 6 | 617 | 634 | 649 |
| contig01228-1 | att | 6 | 13 | 30 | 925 |
| contig02189-1 | att | 5 | 826 | 840 | 1960 |
| contig10332-1 | att | 7 | 332 | 352 | 461 |
| contig11706-1 | att | 5 | 106 | 120 | 580 |
| contig12359-1 | att | 18 | 198 | 251 | 349 |
| FW1NBNE02C6LM6-1 | att | 9 | 195 | 221 | 600 |
| FW1NBNE02DTJ34-1 | att | 6 | 199 | 216 | 252 |
| FW1NBNE02C9R0C-1 | att | 5 | 104 | 118 | 248 |
| FW1NBNE02EL96P-1 | att | 6 | 180 | 197 | 273 |
| FW1NBNE02ESVYX-1 | att | 5 | 46 | 60 | 490 |
| contig00751-1 | attt | 5 | 9 | 28 | 527 |
| FW1NBNE02C5JL2-2 | atttt | 8 | 1 | 40 | 105 |
| contig00746-2 | ca | 14 | 1298 | 1325 | 1325 |
| contig12275-2 | ca | 5 | 139 | 148 | 460 |
| contig12716-2 | ca | 10 | 372 | 391 | 446 |
| FW1NBNE02DEVZ6-2 | ca | 6 | 35 | 46 | 322 |
| FW1NBNE02DTLAB-2 | ca | 22 | 22 | 65 | 89 |
| contig12716-3 | ca | 9 | 394 | 411 | 446 |
| FW1NBNE02DT559-3 | ca | 5 | 38 | 47 | 385 |
| contig04327-1 | ca | 5 | 62 | 71 | 560 |
| contig06167-1 | ca | 6 | 1 | 12 | 903 |
| contig06790-1 | ca | 7 | 28 | 41 | 819 |
| contig06850-1 | ca | 5 | 120 | 129 | 527 |
| contig08111-1 | ca | 5 | 302 | 311 | 440 |
| contig08828-1 | ca | 9 | 28 | 45 | 670 |
| contig10040-1 | ca | 5 | 66 | 75 | 480 |
| contig13249-1 | ca | 6 | 161 | 172 | 235 |
| FW1NBNE02C6A5U-1 | ca | 9 | 15 | 32 | 455 |
| FW1NBNE02DFB1B-1 | ca | 5 | 67 | 76 | 134 |
| FW1NBNE02DEO73-1 | ca | 5 | 79 | 88 | 417 |
| FW1NBNE02DUKTY-1 | ca | 5 | 121 | 130 | 503 |
| FW1NBNE02DLHZN-1 | ca | 5 | 18 | 27 | 534 |
| FW1NBNE02EEB19-1 | ca | 5 | 44 | 53 | 513 |
| FW1NBNE02D7A6S-1 | ca | 8 | 217 | 232 | 293 |
| FW1NBNE02DDRJD-1 | ca | 5 | 250 | 259 | 453 |
| FW1NBNE02EJJBX-1 | ca | 6 | 37 | 48 | 507 |
| FW1NBNE02D6QMC-1 | ca | 5 | 436 | 445 | 506 |
| FW1NBNE02DBS5P-1 | ca | 10 | 29 | 48 | 383 |
| FW1NBNE02EJYAI-1 | ca | 6 | 12 | 23 | 90 |
| FW1NBNE02D7AYG-1 | ca | 9 | 10 | 27 | 66 |
| FW1NBNE02EON5O-1 | ca | 5 | 89 | 98 | 360 |
| FW1NBNE02DL98R-1 | ca | 9 | 354 | 371 | 404 |
| contig12638-2 | caa | 5 | 267 | 281 | 416 |
| FW1NBNE02DG760-2 | caa | 5 | 14 | 28 | 524 |
| FW1NBNE02DV5AL-3 | caa | 6 | 317 | 334 | 466 |
| contig00055-1 | caa | 9 | 158 | 184 | 321 |
| contig06859-1 | caa | 11 | 131 | 163 | 401 |
| FW1NBNE02DRRIZ-1 | caa | 7 | 180 | 200 | 422 |
| contig01313-1 | caaacc | 5 | 303 | 332 | 999 |
| contig00707-3 | caaata | 5 | 119 | 148 | 1498 |
| FW1NBNE02DYA0Q-2 | caacag | 5 | 309 | 338 | 338 |
| FW1NBNE02C3HR0-2 | cac | 9 | 278 | 304 | 399 |
| FW1NBNE02DBVQ8-2 | cac | 7 | 237 | 257 | 386 |
| contig01556-1 | cac | 5 | 259 | 273 | 542 |
| contig02377-1 | cac | 7 | 72 | 92 | 452 |
| contig08464-1 | cac | 5 | 68 | 82 | 753 |
| contig08845-1 | cac | 5 | 104 | 118 | 603 |
| contig12202-1 | cac | 5 | 450 | 464 | 465 |
| contig12725-1 | cac | 6 | 74 | 91 | 394 |
| contig13223-1 | cac | 5 | 362 | 376 | 661 |
| FW1NBNE02D4Q17-1 | cac | 6 | 99 | 116 | 292 |
| FW1NBNE02EESV2-1 | cac | 8 | 159 | 182 | 384 |
| FW1NBNE02EAVUZ-1 | cac | 5 | 191 | 205 | 519 |
| FW1NBNE02EVLUZ-1 | cac | 5 | 19 | 33 | 391 |
| FW1NBNE02DGBAQ-1 | cac | 8 | 48 | 71 | 141 |
| contig01598-1 | cact | 5 | 842 | 861 | 911 |
| FW1NBNE02DNLSJ-2 | cag | 5 | 171 | 185 | 414 |
| FW1NBNE02D2KZU-2 | cag | 5 | 228 | 242 | 410 |
| FW1NBNE02EXIMI-2 | cag | 8 | 380 | 403 | 403 |
| FW1NBNE02DMP9Q-2 | cag | 5 | 270 | 284 | 469 |
| contig04029-1 | cag | 5 | 184 | 198 | 477 |
| contig04472-1 | cag | 5 | 221 | 235 | 528 |
| contig05747-1 | cag | 6 | 309 | 326 | 730 |
| contig05810-1 | cag | 5 | 429 | 443 | 667 |
| contig06243-1 | cag | 5 | 317 | 331 | 432 |
| contig06803-1 | cag | 6 | 323 | 340 | 2845 |
| contig08474-1 | cag | 6 | 108 | 125 | 658 |
| contig09451-1 | cag | 6 | 402 | 419 | 419 |
| contig10251-1 | cag | 5 | 6 | 20 | 1560 |
| contig11278-1 | cag | 5 | 255 | 269 | 462 |
| contig11986-1 | cag | 6 | 335 | 352 | 418 |
| contig12848-1 | cag | 6 | 30 | 47 | 419 |
| contig12906-1 | cag | 5 | 51 | 65 | 411 |
| contig13807-1 | cag | 5 | 41 | 55 | 282 |
| FW1NBNE02DT4Q1-1 | cag | 5 | 33 | 47 | 52 |
| FW1NBNE02DADIS-1 | cag | 6 | 176 | 193 | 445 |
| FW1NBNE02D8ZNJ-1 | cag | 7 | 134 | 154 | 358 |
| FW1NBNE02EXIMI-1 | cag | 12 | 299 | 334 | 403 |
| FW1NBNE02ENJL2-1 | cag | 7 | 141 | 161 | 364 |
| FW1NBNE02EYDX4-1 | cag | 7 | 216 | 236 | 258 |
| FW1NBNE02DYA0Q-1 | cag | 5 | 120 | 134 | 338 |
| FW1NBNE02D6YXB-1 | cag | 6 | 39 | 56 | 412 |
| FW1NBNE02EC1KB-1 | cag | 5 | 4 | 18 | 478 |
| FW1NBNE02ELEG0-1 | cag | 5 | 180 | 194 | 461 |
| FW1NBNE02EOPAN-1 | cag | 6 | 137 | 154 | 435 |
| contig04435-1 | cagg | 5 | 203 | 222 | 652 |
| FW1NBNE02DMCIZ-1 | cagt | 5 | 333 | 352 | 435 |
| contig00444-1 | cat | 5 | 163 | 177 | 502 |
| contig01084-1 | cat | 5 | 89 | 103 | 469 |
| contig01627-1 | cat | 5 | 1122 | 1136 | 1323 |
| contig03811-1 | cat | 7 | 76 | 96 | 355 |
| contig04381-1 | cat | 5 | 122 | 136 | 379 |
| contig05719-1 | cat | 5 | 233 | 247 | 961 |
| contig06188-1 | cat | 5 | 1127 | 1141 | 1142 |
| contig06429-1 | cat | 6 | 829 | 846 | 857 |
| contig09731-1 | cat | 5 | 41 | 55 | 414 |
| contig09921-1 | cat | 6 | 100 | 117 | 283 |
| contig11510-1 | cat | 5 | 463 | 477 | 478 |
| contig11911-1 | cat | 5 | 348 | 362 | 422 |
| FW1NBNE02DNNGK-1 | cat | 6 | 157 | 174 | 511 |
| FW1NBNE02EWP9B-1 | cat | 5 | 473 | 487 | 494 |
| FW1NBNE02C1NML-1 | cat | 8 | 340 | 363 | 366 |
| FW1NBNE02EIBRC-1 | cat | 5 | 39 | 53 | 492 |
| FW1NBNE02EW5MJ-1 | cat | 7 | 91 | 111 | 371 |
| FW1NBNE02EC29A-1 | cat | 5 | 361 | 375 | 492 |
| FW1NBNE02DPZSV-1 | cat | 5 | 16 | 30 | 113 |
| FW1NBNE02D0NZS-1 | cat | 11 | 168 | 200 | 204 |
| FW1NBNE02DTNMY-1 | cat | 5 | 25 | 39 | 78 |
| contig11172-1 | catt | 5 | 4 | 23 | 218 |
| contig02528-1 | cca | 5 | 99 | 113 | 694 |
| contig03102-1 | cca | 5 | 463 | 477 | 1404 |
| contig06022-1 | cca | 5 | 257 | 271 | 885 |
| contig07204-1 | cca | 5 | 126 | 140 | 1133 |
| contig10556-1 | cca | 5 | 377 | 391 | 489 |
| contig13375-1 | cca | 5 | 67 | 81 | 205 |
| FW1NBNE02D2KZU-1 | cca | 7 | 197 | 217 | 410 |
| FW1NBNE02C0RAA-1 | cca | 5 | 125 | 139 | 349 |
| FW1NBNE02D0G61-1 | cca | 5 | 454 | 468 | 544 |
| FW1NBNE02D6N9I-1 | cca | 5 | 224 | 238 | 468 |
| FW1NBNE02D4L0L-1 | cca | 8 | 274 | 297 | 378 |
| FW1NBNE02C2FIH-1 | cca | 6 | 265 | 282 | 484 |
| contig00869-2 | ccg | 5 | 319 | 333 | 2487 |
| contig01667-1 | ccg | 6 | 186 | 203 | 1020 |
| FW1NBNE02DSHR6-1 | ccg | 6 | 303 | 320 | 526 |
| FW1NBNE02EQS17-1 | ccg | 9 | 102 | 128 | 151 |
| FW1NBNE02DLBB5-1 | ccg | 6 | 118 | 135 | 523 |
| FW1NBNE02EENBR-1 | ccg | 5 | 143 | 157 | 206 |
| FW1NBNE02DA5PH-1 | ccgctc | 5 | 298 | 327 | 378 |
| contig12011-2 | cct | 5 | 147 | 161 | 431 |
| contig00489-1 | cct | 5 | 207 | 221 | 1121 |
| contig02201-1 | cct | 5 | 252 | 266 | 515 |
| contig02862-1 | cct | 5 | 58 | 72 | 182 |
| contig07024-1 | cct | 5 | 253 | 267 | 295 |
| contig09008-1 | cct | 5 | 318 | 332 | 861 |
| contig09034-1 | cct | 5 | 105 | 119 | 416 |
| contig09111-1 | cct | 7 | 156 | 176 | 1281 |
| FW1NBNE02C4WCM-1 | cct | 5 | 139 | 153 | 517 |
| FW1NBNE02DKJ81-1 | cct | 5 | 63 | 77 | 532 |
| FW1NBNE02DFNT0-1 | cct | 6 | 10 | 27 | 530 |
| FW1NBNE02DSYCI-1 | cct | 5 | 251 | 265 | 333 |
| FW1NBNE02DM52O-1 | cct | 5 | 296 | 310 | 460 |
| FW1NBNE02EAQYS-1 | cct | 5 | 136 | 150 | 497 |
| FW1NBNE02DLSLS-1 | cct | 5 | 122 | 136 | 499 |
| FW1NBNE02EUVCE-1 | cctcaa | 9 | 46 | 99 | 520 |
| contig08517-1 | cctg | 6 | 187 | 210 | 748 |
| contig12966-1 | cctt | 5 | 27 | 46 | 418 |
| contig06013-1 | cg | 5 | 448 | 457 | 517 |
| contig08606-1 | cga | 7 | 137 | 157 | 1824 |
| FW1NBNE02D9Q3D-1 | cga | 9 | 190 | 216 | 496 |
| contig01298-1 | cgc | 6 | 250 | 267 | 710 |
| contig03161-1 | cgc | 5 | 138 | 152 | 1155 |
| FW1NBNE02EOVZZ-1 | cgc | 5 | 35 | 49 | 51 |
| FW1NBNE02EDL7A-1 | cgc | 7 | 257 | 277 | 302 |
| contig04850-1 | cgct | 5 | 246 | 265 | 397 |
| contig01309-1 | cgg | 6 | 15 | 32 | 549 |
| contig12471-1 | cgg | 5 | 32 | 46 | 353 |
| FW1NBNE02C1YTP-2 | cggaag | 17 | 344 | 445 | 454 |
| contig09631-1 | cggagt | 5 | 159 | 188 | 437 |
| FW1NBNE02C36O2-1 | cgt | 6 | 246 | 263 | 511 |
| contig03873-2 | ct | 6 | 559 | 570 | 571 |
| contig04316-2 | ct | 5 | 493 | 502 | 568 |
| contig04866-2 | ct | 9 | 1690 | 1707 | 1736 |
| contig04981-2 | ct | 5 | 1109 | 1118 | 1152 |
| contig07753-2 | ct | 5 | 986 | 995 | 1001 |
| contig10624-2 | ct | 11 | 302 | 323 | 367 |
| contig12947-2 | ct | 10 | 63 | 82 | 415 |
| contig13049-2 | ct | 6 | 241 | 252 | 276 |
| FW1NBNE02C080M-2 | ct | 8 | 202 | 217 | 252 |
| FW1NBNE02EDA38-2 | ct | 5 | 109 | 118 | 502 |
| FW1NBNE02EQNWY-2 | ct | 9 | 50 | 67 | 74 |
| FW1NBNE02D018T-2 | ct | 7 | 420 | 433 | 439 |
| FW1NBNE02D01S4-2 | ct | 5 | 80 | 89 | 338 |
| FW1NBNE02EHUYI-2 | ct | 5 | 228 | 237 | 505 |
| FW1NBNE02EKBWX-2 | ct | 5 | 101 | 110 | 470 |
| FW1NBNE02CZKHT-2 | ct | 9 | 203 | 220 | 238 |
| FW1NBNE02CY79A-2 | ct | 7 | 69 | 82 | 93 |
| FW1NBNE02DI2YQ-2 | ct | 7 | 33 | 46 | 61 |
| FW1NBNE02EK9AW-2 | ct | 5 | 340 | 349 | 432 |
| FW1NBNE02ESBZR-2 | ct | 5 | 83 | 92 | 130 |
| FW1NBNE02EIRPI-2 | ct | 6 | 294 | 305 | 323 |
| FW1NBNE02DEOHK-2 | ct | 7 | 60 | 73 | 84 |
| contig04423-3 | ct | 5 | 740 | 749 | 813 |
| contig00056-1 | ct | 7 | 32 | 45 | 1399 |
| contig00152-1 | ct | 9 | 248 | 265 | 373 |
| contig00352-1 | ct | 5 | 59 | 68 | 584 |
| contig00485-1 | ct | 6 | 72 | 83 | 2689 |
| contig00743-1 | ct | 5 | 139 | 148 | 1859 |
| contig00869-1 | ct | 9 | 2 | 19 | 2487 |
| contig00887-1 | ct | 5 | 810 | 819 | 1539 |
| contig00971-1 | ct | 5 | 180 | 189 | 1329 |
| contig01088-1 | ct | 5 | 104 | 113 | 963 |
| contig01342-1 | ct | 9 | 204 | 221 | 834 |
| contig01438-1 | ct | 8 | 76 | 91 | 1328 |
| contig01518-1 | ct | 8 | 1397 | 1412 | 1504 |
| contig01519-1 | ct | 6 | 932 | 943 | 974 |
| contig01675-1 | ct | 6 | 113 | 124 | 2184 |
| contig01682-1 | ct | 7 | 1357 | 1370 | 1423 |
| contig01721-1 | ct | 10 | 1897 | 1916 | 2000 |
| contig01853-1 | ct | 6 | 80 | 91 | 538 |
| contig01885-1 | ct | 11 | 995 | 1016 | 1017 |
| contig01889-1 | ct | 5 | 73 | 82 | 389 |
| contig01927-1 | ct | 6 | 267 | 278 | 472 |
| contig01998-1 | ct | 6 | 21 | 32 | 404 |
| contig02077-1 | ct | 6 | 46 | 57 | 138 |
| contig02250-1 | ct | 8 | 458 | 473 | 1579 |
| contig02272-1 | ct | 5 | 1305 | 1314 | 1410 |
| contig02281-1 | ct | 5 | 137 | 146 | 398 |
| contig02351-1 | ct | 5 | 474 | 483 | 487 |
| contig02354-1 | ct | 6 | 1062 | 1073 | 1108 |
| contig02372-1 | ct | 5 | 364 | 373 | 1241 |
| contig02383-1 | ct | 5 | 92 | 101 | 618 |
| contig02902-1 | ct | 5 | 75 | 84 | 1348 |
| contig03082-1 | ct | 5 | 134 | 143 | 1479 |
| contig03371-1 | ct | 6 | 54 | 65 | 285 |
| contig03528-1 | ct | 6 | 32 | 43 | 1047 |
| contig03820-1 | ct | 5 | 34 | 43 | 529 |
| contig03988-1 | ct | 5 | 87 | 96 | 393 |
| contig04014-1 | ct | 5 | 75 | 84 | 699 |
| contig04071-1 | ct | 8 | 449 | 464 | 624 |
| contig04506-1 | ct | 5 | 177 | 186 | 2261 |
| contig04545-1 | ct | 5 | 4 | 13 | 480 |
| contig04817-1 | ct | 5 | 105 | 114 | 523 |
| contig05212-1 | ct | 5 | 649 | 658 | 1002 |
| contig05358-1 | ct | 5 | 79 | 88 | 563 |
| contig05375-1 | ct | 9 | 1059 | 1076 | 1130 |
| contig05685-1 | ct | 5 | 34 | 43 | 320 |
| contig05763-1 | ct | 6 | 693 | 704 | 743 |
| contig06374-1 | ct | 6 | 732 | 743 | 768 |
| contig06389-1 | ct | 6 | 760 | 771 | 773 |
| contig06660-1 | ct | 5 | 60 | 69 | 812 |
| contig06740-1 | ct | 5 | 154 | 163 | 259 |
| contig06969-1 | ct | 8 | 1292 | 1307 | 1344 |
| contig06978-1 | ct | 6 | 482 | 493 | 495 |
| contig06990-1 | ct | 5 | 86 | 95 | 179 |
| contig07184-1 | ct | 6 | 140 | 151 | 1025 |
| contig07263-1 | ct | 5 | 74 | 83 | 1283 |
| contig07569-1 | ct | 5 | 56 | 65 | 534 |
| contig07710-1 | ct | 6 | 57 | 68 | 485 |
| contig07820-1 | ct | 7 | 87 | 100 | 498 |
| contig07908-1 | ct | 7 | 35 | 48 | 1144 |
| contig08132-1 | ct | 5 | 645 | 654 | 789 |
| contig08236-1 | ct | 6 | 758 | 769 | 790 |
| contig08262-1 | ct | 7 | 905 | 918 | 1138 |
| contig08394-1 | ct | 5 | 361 | 370 | 406 |
| contig08412-1 | ct | 6 | 667 | 678 | 706 |
| contig08461-1 | ct | 5 | 33 | 42 | 706 |
| contig08511-1 | ct | 6 | 39 | 50 | 795 |
| contig08600-1 | ct | 11 | 425 | 446 | 481 |
| contig08618-1 | ct | 5 | 312 | 321 | 567 |
| contig08625-1 | ct | 5 | 302 | 311 | 1886 |
| contig08632-1 | ct | 5 | 140 | 149 | 508 |
| contig08643-1 | ct | 5 | 13 | 22 | 492 |
| contig08843-1 | ct | 5 | 215 | 224 | 480 |
| contig09322-1 | ct | 5 | 531 | 540 | 569 |
| contig09502-1 | ct | 6 | 393 | 404 | 460 |
| contig10457-1 | ct | 7 | 29 | 42 | 459 |
| contig10898-1 | ct | 5 | 64 | 73 | 220 |
| contig11041-1 | ct | 5 | 17 | 26 | 421 |
| contig11646-1 | ct | 6 | 56 | 67 | 670 |
| contig11772-1 | ct | 6 | 407 | 418 | 444 |
| contig11825-1 | ct | 9 | 282 | 299 | 331 |
| contig11861-1 | ct | 5 | 74 | 83 | 161 |
| contig11873-1 | ct | 5 | 3 | 12 | 423 |
| contig12012-1 | ct | 9 | 264 | 281 | 281 |
| contig12084-1 | ct | 6 | 92 | 103 | 471 |
| contig12295-1 | ct | 6 | 449 | 460 | 465 |
| contig12339-1 | ct | 5 | 265 | 274 | 914 |
| contig12499-1 | ct | 5 | 325 | 334 | 369 |
| contig12587-1 | ct | 9 | 48 | 65 | 267 |
| contig12664-1 | ct | 5 | 38 | 47 | 272 |
| contig12729-1 | ct | 6 | 16 | 27 | 440 |
| contig12891-1 | ct | 7 | 289 | 302 | 307 |
| contig13049-1 | ct | 6 | 9 | 20 | 276 |
| contig13192-1 | ct | 5 | 307 | 316 | 355 |
| contig13216-1 | ct | 5 | 48 | 57 | 253 |
| contig13259-1 | ct | 8 | 42 | 57 | 294 |
| contig13333-1 | ct | 9 | 242 | 259 | 344 |
| contig13725-1 | ct | 7 | 607 | 620 | 787 |
| contig13941-1 | ct | 5 | 75 | 84 | 121 |
| contig14003-1 | ct | 5 | 71 | 80 | 172 |
| FW1NBNE02DV10E-1 | ct | 8 | 37 | 52 | 53 |
| FW1NBNE02DVO53-1 | ct | 5 | 21 | 30 | 225 |
| FW1NBNE02ENMVV-1 | ct | 5 | 163 | 172 | 373 |
| FW1NBNE02EMULS-1 | ct | 6 | 303 | 314 | 345 |
| FW1NBNE02DP0QR-1 | ct | 5 | 68 | 77 | 332 |
| FW1NBNE02C9FZT-1 | ct | 5 | 174 | 183 | 387 |
| FW1NBNE02D75Y8-1 | ct | 5 | 54 | 63 | 199 |
| FW1NBNE02DEVZ6-1 | ct | 7 | 21 | 34 | 322 |
| FW1NBNE02D4MTD-1 | ct | 15 | 89 | 118 | 118 |
| FW1NBNE02DL5CE-1 | ct | 6 | 71 | 82 | 225 |
| FW1NBNE02D18QE-1 | ct | 5 | 276 | 285 | 514 |
| FW1NBNE02EPYQJ-1 | ct | 5 | 88 | 97 | 491 |
| FW1NBNE02DGCVP-1 | ct | 5 | 2 | 11 | 486 |
| FW1NBNE02D5G8T-1 | ct | 9 | 27 | 44 | 87 |
| FW1NBNE02ETIZL-1 | ct | 5 | 138 | 147 | 152 |
| FW1NBNE02DUJE7-1 | ct | 6 | 509 | 520 | 521 |
| FW1NBNE02C7B21-1 | ct | 5 | 75 | 84 | 441 |
| FW1NBNE02DDA1N-1 | ct | 5 | 93 | 102 | 402 |
| FW1NBNE02DE7XT-1 | ct | 16 | 33 | 64 | 65 |
| FW1NBNE02DFUEX-1 | ct | 8 | 1 | 16 | 250 |
| FW1NBNE02EANLD-1 | ct | 9 | 312 | 329 | 414 |
| FW1NBNE02DDSON-1 | ct | 7 | 33 | 46 | 451 |
| FW1NBNE02DP3ON-1 | ct | 7 | 82 | 95 | 490 |
| FW1NBNE02DI4GZ-1 | ct | 5 | 3 | 12 | 57 |
| FW1NBNE02DK23E-1 | ct | 6 | 251 | 262 | 341 |
| FW1NBNE02C67IU-1 | ct | 9 | 11 | 28 | 483 |
| FW1NBNE02EM67O-1 | ct | 5 | 90 | 99 | 258 |
| FW1NBNE02EF054-1 | ct | 8 | 10 | 25 | 467 |
| FW1NBNE02DOFHD-1 | ct | 5 | 56 | 65 | 191 |
| FW1NBNE02EHRB1-1 | ct | 9 | 109 | 126 | 197 |
| FW1NBNE02DUNBY-1 | ct | 8 | 44 | 59 | 77 |
| FW1NBNE02D7KU1-1 | ct | 6 | 40 | 51 | 448 |
| FW1NBNE02EUBP1-1 | ct | 5 | 56 | 65 | 208 |
| FW1NBNE02DTLAB-1 | ct | 5 | 4 | 13 | 89 |
| FW1NBNE02EHAL3-1 | ct | 6 | 41 | 52 | 362 |
| FW1NBNE02DRFFY-1 | ct | 5 | 90 | 99 | 531 |
| FW1NBNE02EOOXC-1 | ct | 6 | 25 | 36 | 252 |
| FW1NBNE02C6LIC-1 | ct | 5 | 12 | 21 | 513 |
| FW1NBNE02D01V5-1 | ct | 5 | 334 | 343 | 498 |
| FW1NBNE02D01S4-1 | ct | 6 | 11 | 22 | 338 |
| FW1NBNE02ERX49-1 | ct | 11 | 81 | 102 | 108 |
| FW1NBNE02D2D3D-1 | ct | 5 | 285 | 294 | 488 |
| FW1NBNE02DDJ1Z-1 | ct | 5 | 2 | 11 | 443 |
| FW1NBNE02EMH3J-1 | ct | 7 | 19 | 32 | 81 |
| FW1NBNE02EDN8B-1 | ct | 5 | 522 | 531 | 534 |
| FW1NBNE02ESE29-1 | ct | 8 | 7 | 22 | 70 |
| FW1NBNE02DNPQT-1 | ct | 6 | 537 | 548 | 567 |
| FW1NBNE02D2NXX-1 | ct | 6 | 112 | 123 | 388 |
| FW1NBNE02EQGDT-1 | ct | 5 | 255 | 264 | 520 |
| FW1NBNE02EWLP1-1 | ct | 7 | 32 | 45 | 362 |
| FW1NBNE02EID0C-1 | ct | 5 | 82 | 91 | 127 |
| FW1NBNE02D5E2J-1 | ct | 5 | 87 | 96 | 495 |
| FW1NBNE02ED9XU-1 | ct | 7 | 1 | 14 | 462 |
| FW1NBNE02DMV2M-1 | ct | 8 | 48 | 63 | 63 |
| FW1NBNE02DPL5F-1 | ct | 6 | 72 | 83 | 353 |
| FW1NBNE02EC0LV-1 | ct | 5 | 60 | 69 | 411 |
| FW1NBNE02DLDG6-1 | ct | 5 | 347 | 356 | 502 |
| FW1NBNE02DQ7Z6-1 | ct | 6 | 6 | 17 | 266 |
| FW1NBNE02EVVPZ-1 | ct | 6 | 16 | 27 | 251 |
| FW1NBNE02DXU9C-1 | ct | 5 | 88 | 97 | 482 |
| FW1NBNE02EPCKY-1 | ct | 5 | 33 | 42 | 190 |
| FW1NBNE02EKBWX-1 | ct | 5 | 58 | 67 | 470 |
| FW1NBNE02D3SXB-1 | ct | 7 | 17 | 30 | 465 |
| FW1NBNE02D9CKX-1 | ct | 9 | 41 | 58 | 61 |
| FW1NBNE02DUBKK-1 | ct | 5 | 30 | 39 | 479 |
| FW1NBNE02DQIYQ-1 | ct | 6 | 56 | 67 | 456 |
| FW1NBNE02DDCY3-1 | ct | 5 | 26 | 35 | 153 |
| FW1NBNE02DV64I-1 | ct | 7 | 34 | 47 | 474 |
| FW1NBNE02D5U69-1 | ct | 11 | 224 | 245 | 363 |
| FW1NBNE02DETTC-1 | ct | 8 | 167 | 182 | 469 |
| FW1NBNE02D2M74-1 | ct | 5 | 14 | 23 | 424 |
| FW1NBNE02DHY2V-1 | ct | 5 | 196 | 205 | 490 |
| FW1NBNE02DV5AL-1 | ct | 6 | 396 | 407 | 466 |
| FW1NBNE02DYAOT-1 | ct | 5 | 15 | 24 | 168 |
| FW1NBNE02DG7N4-1 | ct | 7 | 35 | 48 | 106 |
| FW1NBNE02DAA0W-1 | ct | 6 | 400 | 411 | 411 |
| FW1NBNE02DLL5G-1 | ct | 5 | 184 | 193 | 259 |
| FW1NBNE02CZ2CR-1 | ct | 7 | 18 | 31 | 343 |
| FW1NBNE02DQL6Z-1 | ct | 5 | 58 | 67 | 136 |
| FW1NBNE02DSN6P-1 | ct | 8 | 248 | 263 | 359 |
| FW1NBNE02DHB2R-1 | ct | 6 | 29 | 40 | 329 |
| FW1NBNE02EEZQN-1 | ct | 5 | 8 | 17 | 376 |
| FW1NBNE02EO6G6-1 | ct | 5 | 82 | 91 | 440 |
| FW1NBNE02DQVZ7-1 | ct | 9 | 100 | 117 | 136 |
| FW1NBNE02CY79A-1 | ct | 9 | 28 | 45 | 93 |
| FW1NBNE02EL5D6-1 | ct | 7 | 57 | 70 | 91 |
| FW1NBNE02DI2YQ-1 | ct | 5 | 22 | 31 | 61 |
| FW1NBNE02EVS3L-1 | ct | 10 | 53 | 72 | 94 |
| FW1NBNE02DO4J6-1 | ct | 7 | 30 | 43 | 248 |
| FW1NBNE02DNLQ7-1 | ct | 5 | 39 | 48 | 61 |
| FW1NBNE02DZRDB-1 | ct | 6 | 129 | 140 | 268 |
| FW1NBNE02DNZCF-1 | ct | 7 | 36 | 49 | 407 |
| FW1NBNE02C88Q8-1 | ct | 5 | 14 | 23 | 411 |
| FW1NBNE02DXO7A-1 | ct | 5 | 22 | 31 | 494 |
| FW1NBNE02EQECK-1 | ct | 6 | 125 | 136 | 443 |
| FW1NBNE02C46NJ-1 | ct | 5 | 90 | 99 | 377 |
| FW1NBNE02ENPMF-1 | ct | 5 | 337 | 346 | 366 |
| FW1NBNE02EW0P1-1 | ct | 5 | 175 | 184 | 329 |
| FW1NBNE02C0KHK-1 | ct | 5 | 72 | 81 | 316 |
| FW1NBNE02EEMOM-1 | ct | 8 | 474 | 489 | 501 |
| FW1NBNE02DFX07-1 | ct | 6 | 68 | 79 | 84 |
| FW1NBNE02EX0P3-1 | ct | 5 | 87 | 96 | 301 |
| FW1NBNE02ET1N8-1 | ct | 6 | 31 | 42 | 274 |
| FW1NBNE02DEQ9J-1 | ct | 11 | 70 | 91 | 92 |
| FW1NBNE02C8XZO-1 | ct | 5 | 50 | 59 | 238 |
| FW1NBNE02EQT3H-1 | ct | 5 | 70 | 79 | 406 |
| FW1NBNE02EJZ3K-1 | ct | 5 | 85 | 94 | 186 |
| FW1NBNE02DDIU9-1 | ct | 5 | 119 | 128 | 309 |
| FW1NBNE02ESF2E-1 | ct | 8 | 152 | 167 | 214 |
| FW1NBNE02DEOHK-1 | ct | 8 | 20 | 35 | 84 |
| FW1NBNE02C3XB3-1 | ct | 5 | 31 | 40 | 340 |
| FW1NBNE02D2VCN-1 | ct | 5 | 10 | 19 | 164 |
| FW1NBNE02EEOXG-1 | ct | 7 | 179 | 192 | 233 |
| FW1NBNE02EJDA3-2 | cta | 5 | 20 | 34 | 162 |
| contig04161-1 | cta | 5 | 155 | 169 | 296 |
| contig10351-1 | cta | 6 | 231 | 248 | 290 |
| contig12962-1 | cta | 5 | 401 | 415 | 415 |
| FW1NBNE02CZR2W-1 | cta | 9 | 326 | 352 | 354 |
| FW1NBNE02EQLLO-1 | cta | 5 | 113 | 127 | 581 |
| FW1NBNE02C2JAD-1 | cta | 5 | 265 | 279 | 341 |
| contig01443-1 | ctaaac | 5 | 138 | 167 | 608 |
| FW1NBNE02ENPMF-2 | ctac | 5 | 345 | 364 | 366 |
| contig01375-2 | ctag | 5 | 63 | 82 | 184 |
| contig00157-1 | ctag | 5 | 48 | 67 | 1470 |
| contig01376-1 | ctag | 5 | 67 | 86 | 134 |
| contig02297-1 | ctat | 5 | 388 | 407 | 487 |
| contig03215-1 | ctat | 12 | 519 | 566 | 617 |
| contig02006-2 | ctc | 5 | 187 | 201 | 1019 |
| contig04506-2 | ctc | 6 | 85 | 102 | 2261 |
| contig01404-1 | ctc | 5 | 1579 | 1593 | 2348 |
| contig02006-1 | ctc | 5 | 36 | 50 | 1019 |
| contig03607-1 | ctc | 5 | 149 | 163 | 1108 |
| contig11250-1 | ctc | 6 | 125 | 142 | 453 |
| contig11653-1 | ctc | 6 | 2 | 19 | 452 |
| contig12762-1 | ctc | 5 | 26 | 40 | 223 |
| FW1NBNE02D3JO0-1 | ctc | 6 | 82 | 99 | 156 |
| FW1NBNE02DXFIO-1 | ctc | 5 | 183 | 197 | 376 |
| FW1NBNE02EVGN3-1 | ctc | 5 | 40 | 54 | 406 |
| contig04607-1 | ctca | 5 | 2046 | 2065 | 2083 |
| FW1NBNE02DQYBG-1 | ctcttc | 5 | 186 | 215 | 429 |
| contig09408-2 | ctg | 5 | 1004 | 1018 | 1259 |
| FW1NBNE02DVR7E-2 | ctg | 6 | 398 | 415 | 485 |
| contig00474-1 | ctg | 6 | 383 | 400 | 1207 |
| contig02450-1 | ctg | 6 | 767 | 784 | 1264 |
| contig06202-1 | ctg | 5 | 402 | 416 | 518 |
| contig07012-1 | ctg | 6 | 951 | 968 | 1237 |
| contig07225-1 | ctg | 6 | 202 | 219 | 541 |
| contig07375-1 | ctg | 5 | 201 | 215 | 231 |
| contig09073-1 | ctg | 7 | 457 | 477 | 716 |
| contig11604-1 | ctg | 6 | 39 | 56 | 759 |
| contig12201-1 | ctg | 5 | 172 | 186 | 302 |
| contig12496-1 | ctg | 6 | 184 | 201 | 416 |
| contig13514-1 | ctg | 5 | 20 | 34 | 107 |
| FW1NBNE02DU3UI-1 | ctg | 5 | 412 | 426 | 507 |
| FW1NBNE02DZAM6-1 | ctg | 7 | 121 | 141 | 444 |
| FW1NBNE02DBJ4Y-1 | ctg | 8 | 189 | 212 | 236 |
| FW1NBNE02DKLBS-1 | ctg | 5 | 331 | 345 | 480 |
| FW1NBNE02DDQU5-1 | ctg | 5 | 92 | 106 | 156 |
| FW1NBNE02EPD8K-1 | ctg | 5 | 134 | 148 | 353 |
| contig02487-1 | ctgat | 5 | 216 | 240 | 428 |
| FW1NBNE02EVGN3-2 | ctggag | 5 | 161 | 190 | 406 |
| contig07036-1 | ctgt | 8 | 1813 | 1844 | 1973 |
| contig13804-2 | ctt | 5 | 115 | 129 | 459 |
| contig02250-3 | ctt | 5 | 219 | 233 | 1579 |
| FW1NBNE02ESBZR-4 | ctt | 7 | 25 | 45 | 130 |
| contig00778-1 | ctt | 6 | 401 | 418 | 1039 |
| contig01102-1 | ctt | 5 | 757 | 771 | 914 |
| contig01963-1 | ctt | 6 | 84 | 101 | 891 |
| contig05100-1 | ctt | 5 | 518 | 532 | 564 |
| contig06165-1 | ctt | 5 | 404 | 418 | 506 |
| contig10014-1 | ctt | 5 | 92 | 106 | 504 |
| FW1NBNE02DKHY7-1 | ctt | 5 | 216 | 230 | 239 |
| FW1NBNE02DJ5EM-1 | ctt | 5 | 236 | 250 | 498 |
| FW1NBNE02DIJ0O-1 | ctt | 9 | 139 | 165 | 191 |
| FW1NBNE02ECM40-1 | ctt | 5 | 192 | 206 | 519 |
| FW1NBNE02ESK1N-1 | ctt | 5 | 218 | 232 | 524 |
| FW1NBNE02ESBCJ-1 | ctt | 5 | 279 | 293 | 519 |
| FW1NBNE02D69CX-1 | ctt | 5 | 346 | 360 | 431 |
| FW1NBNE02DUUGV-1 | ctt | 5 | 398 | 412 | 454 |
| FW1NBNE02EQ9GS-1 | ctt | 7 | 53 | 73 | 261 |
| FW1NBNE02D04I8-1 | ctt | 17 | 347 | 397 | 397 |
| FW1NBNE02ETYMV-1 | ctt | 5 | 158 | 172 | 278 |
| FW1NBNE02C839P-1 | ctt | 8 | 470 | 493 | 494 |
| FW1NBNE02C8IS9-1 | ctt | 5 | 123 | 137 | 480 |
| FW1NBNE02DPCH1-1 | ctt | 6 | 159 | 176 | 248 |
| contig08205-1 | cttt | 5 | 217 | 236 | 480 |
| contig00323-3 | cttttc | 5 | 76 | 105 | 423 |
| FW1NBNE02C59HZ-3 | cttttc | 6 | 380 | 415 | 522 |
| contig00942-2 | ga | 7 | 1751 | 1764 | 1787 |
| contig01438-2 | ga | 5 | 1312 | 1321 | 1328 |
| contig01721-2 | ga | 5 | 1934 | 1943 | 2000 |
| contig02153-2 | ga | 5 | 891 | 900 | 961 |
| contig02272-2 | ga | 9 | 1350 | 1367 | 1410 |
| contig02276-2 | ga | 5 | 810 | 819 | 945 |
| contig02682-2 | ga | 5 | 1167 | 1176 | 1314 |
| contig04423-2 | ga | 5 | 441 | 450 | 813 |
| contig06713-2 | ga | 5 | 1682 | 1691 | 1705 |
| contig07261-2 | ga | 8 | 1033 | 1048 | 1050 |
| FW1NBNE02EPN2P-2 | ga | 5 | 80 | 89 | 110 |
| FW1NBNE02DZQ64-2 | ga | 5 | 181 | 190 | 490 |
| FW1NBNE02C42VJ-2 | ga | 9 | 79 | 96 | 100 |
| FW1NBNE02DTOFW-2 | ga | 8 | 500 | 515 | 516 |
| FW1NBNE02DSGPY-2 | ga | 5 | 64 | 73 | 371 |
| FW1NBNE02DFS7D-2 | ga | 5 | 344 | 353 | 528 |
| FW1NBNE02C3VJZ-2 | ga | 7 | 144 | 157 | 297 |
| FW1NBNE02DOWZR-2 | ga | 7 | 469 | 482 | 487 |
| FW1NBNE02D62BK-2 | ga | 5 | 326 | 335 | 439 |
| FW1NBNE02DZ7W7-3 | ga | 7 | 187 | 200 | 326 |
| FW1NBNE02EOR8E-3 | ga | 15 | 56 | 85 | 97 |
| contig00167-1 | ga | 7 | 1497 | 1510 | 1514 |
| contig00222-1 | ga | 7 | 557 | 570 | 1446 |
| contig00244-1 | ga | 5 | 89 | 98 | 199 |
| contig00314-1 | ga | 6 | 506 | 517 | 532 |
| contig00465-1 | ga | 8 | 1016 | 1031 | 1197 |
| contig00531-1 | ga | 5 | 770 | 779 | 784 |
| contig00542-1 | ga | 5 | 888 | 897 | 1160 |
| contig00589-1 | ga | 9 | 580 | 597 | 641 |
| contig00612-1 | ga | 6 | 143 | 154 | 1617 |
| contig00741-1 | ga | 11 | 1214 | 1235 | 1369 |
| contig00745-1 | ga | 6 | 1899 | 1910 | 1925 |
| contig00752-1 | ga | 5 | 1188 | 1197 | 1364 |
| contig00863-1 | ga | 6 | 384 | 395 | 446 |
| contig00873-1 | ga | 6 | 24 | 35 | 1245 |
| contig00886-1 | ga | 5 | 1109 | 1118 | 1141 |
| contig00952-1 | ga | 10 | 156 | 175 | 1869 |
| contig00991-1 | ga | 12 | 588 | 611 | 621 |
| contig01059-1 | ga | 5 | 676 | 685 | 741 |
| contig01079-1 | ga | 5 | 1390 | 1399 | 1465 |
| contig01292-1 | ga | 6 | 527 | 538 | 564 |
| contig01356-1 | ga | 6 | 1513 | 1524 | 1702 |
| contig01751-1 | ga | 5 | 1759 | 1768 | 1827 |
| contig01826-1 | ga | 5 | 41 | 50 | 408 |
| contig01987-1 | ga | 5 | 661 | 670 | 675 |
| contig02004-1 | ga | 6 | 899 | 910 | 911 |
| contig02140-1 | ga | 5 | 616 | 625 | 1364 |
| contig02276-1 | ga | 6 | 793 | 804 | 945 |
| contig02400-1 | ga | 6 | 1254 | 1265 | 1266 |
| contig02408-1 | ga | 6 | 473 | 484 | 508 |
| contig02430-1 | ga | 5 | 145 | 154 | 310 |
| contig02643-1 | ga | 5 | 1376 | 1385 | 2281 |
| contig02645-1 | ga | 5 | 707 | 716 | 885 |
| contig02682-1 | ga | 6 | 1105 | 1116 | 1314 |
| contig02928-1 | ga | 5 | 343 | 352 | 379 |
| contig03021-1 | ga | 7 | 211 | 224 | 246 |
| contig03105-1 | ga | 5 | 104 | 113 | 1252 |
| contig03126-1 | ga | 7 | 958 | 971 | 972 |
| contig03157-1 | ga | 5 | 547 | 556 | 633 |
| contig03235-1 | ga | 5 | 956 | 965 | 1011 |
| contig03254-1 | ga | 5 | 1035 | 1044 | 1045 |
| contig03264-1 | ga | 5 | 1595 | 1604 | 1681 |
| contig03287-1 | ga | 5 | 63 | 72 | 241 |
| contig03534-1 | ga | 13 | 1006 | 1031 | 1033 |
| contig03545-1 | ga | 9 | 29 | 46 | 250 |
| contig03557-1 | ga | 5 | 400 | 409 | 1516 |
| contig03600-1 | ga | 5 | 747 | 756 | 898 |
| contig03840-1 | ga | 7 | 303 | 316 | 485 |
| contig03841-1 | ga | 5 | 512 | 521 | 576 |
| contig03876-1 | ga | 7 | 60 | 73 | 625 |
| contig03877-1 | ga | 6 | 615 | 626 | 627 |
| contig03919-1 | ga | 5 | 17 | 26 | 1850 |
| contig03985-1 | ga | 5 | 377 | 386 | 521 |
| contig04094-1 | ga | 5 | 917 | 926 | 1016 |
| contig04116-1 | ga | 5 | 35 | 44 | 320 |
| contig04466-1 | ga | 5 | 491 | 500 | 514 |
| contig04639-1 | ga | 10 | 903 | 922 | 922 |
| contig04776-1 | ga | 5 | 320 | 329 | 359 |
| contig04794-1 | ga | 5 | 131 | 140 | 168 |
| contig04959-1 | ga | 5 | 346 | 355 | 852 |
| contig05064-1 | ga | 5 | 865 | 874 | 920 |
| contig05157-1 | ga | 5 | 1271 | 1280 | 1314 |
| contig05336-1 | ga | 5 | 409 | 418 | 444 |
| contig05374-1 | ga | 5 | 119 | 128 | 446 |
| contig05467-1 | ga | 5 | 161 | 170 | 682 |
| contig05494-1 | ga | 10 | 282 | 301 | 464 |
| contig05519-1 | ga | 8 | 297 | 312 | 387 |
| contig05543-1 | ga | 5 | 388 | 397 | 782 |
| contig05576-1 | ga | 5 | 236 | 245 | 1406 |
| contig05640-1 | ga | 6 | 1650 | 1661 | 1728 |
| contig05872-1 | ga | 5 | 102 | 111 | 536 |
| contig06072-1 | ga | 5 | 625 | 634 | 1415 |
| contig06107-1 | ga | 6 | 67 | 78 | 527 |
| contig06176-1 | ga | 5 | 365 | 374 | 377 |
| contig06347-1 | ga | 11 | 45 | 66 | 1832 |
| contig06439-1 | ga | 5 | 25 | 34 | 336 |
| contig06713-1 | ga | 5 | 1667 | 1676 | 1705 |
| contig07261-1 | ga | 5 | 1002 | 1011 | 1050 |
| contig07276-1 | ga | 5 | 1085 | 1094 | 1468 |
| contig07716-1 | ga | 5 | 218 | 227 | 230 |
| contig07766-1 | ga | 6 | 50 | 61 | 251 |
| contig08065-1 | ga | 5 | 8 | 17 | 340 |
| contig08154-1 | ga | 7 | 262 | 275 | 503 |
| contig08224-1 | ga | 5 | 208 | 217 | 513 |
| contig08295-1 | ga | 6 | 171 | 182 | 433 |
| contig08327-1 | ga | 5 | 30 | 39 | 509 |
| contig08459-1 | ga | 6 | 480 | 491 | 518 |
| contig08649-1 | ga | 5 | 445 | 454 | 671 |
| contig08681-1 | ga | 5 | 24 | 33 | 498 |
| contig08941-1 | ga | 5 | 89 | 98 | 475 |
| contig09116-1 | ga | 6 | 921 | 932 | 948 |
| contig09156-1 | ga | 6 | 599 | 610 | 638 |
| contig09408-1 | ga | 5 | 115 | 124 | 1259 |
| contig09541-1 | ga | 5 | 265 | 274 | 333 |
| contig09566-1 | ga | 7 | 121 | 134 | 700 |
| contig09692-1 | ga | 5 | 137 | 146 | 251 |
| contig10133-1 | ga | 6 | 456 | 467 | 493 |
| contig10147-1 | ga | 7 | 190 | 203 | 583 |
| contig10185-1 | ga | 5 | 39 | 48 | 314 |
| contig10237-1 | ga | 5 | 441 | 450 | 484 |
| contig10252-1 | ga | 7 | 273 | 286 | 492 |
| contig10432-1 | ga | 8 | 857 | 872 | 960 |
| contig10560-1 | ga | 6 | 475 | 486 | 489 |
| contig10624-1 | ga | 5 | 139 | 148 | 367 |
| contig10712-1 | ga | 5 | 438 | 447 | 483 |
| contig11022-1 | ga | 5 | 131 | 140 | 1141 |
| contig11596-1 | ga | 5 | 87 | 96 | 391 |
| contig11630-1 | ga | 5 | 364 | 373 | 477 |
| contig11634-1 | ga | 5 | 352 | 361 | 443 |
| contig11792-1 | ga | 7 | 219 | 232 | 276 |
| contig12025-1 | ga | 5 | 369 | 378 | 558 |
| contig12163-1 | ga | 11 | 252 | 273 | 273 |
| contig12378-1 | ga | 6 | 342 | 353 | 362 |
| contig12633-1 | ga | 9 | 329 | 346 | 374 |
| contig12746-1 | ga | 5 | 195 | 204 | 343 |
| contig12804-1 | ga | 6 | 527 | 538 | 555 |
| contig12814-1 | ga | 5 | 71 | 80 | 264 |
| contig12893-1 | ga | 5 | 350 | 359 | 427 |
| contig12921-1 | ga | 8 | 227 | 242 | 242 |
| contig12934-1 | ga | 6 | 84 | 95 | 421 |
| contig13036-1 | ga | 7 | 65 | 78 | 266 |
| contig13228-1 | ga | 5 | 2 | 11 | 301 |
| contig13607-1 | ga | 5 | 194 | 203 | 345 |
| contig13869-1 | ga | 6 | 167 | 178 | 195 |
| contig13937-1 | ga | 5 | 122 | 131 | 163 |
| FW1NBNE02DCBIF-1 | ga | 15 | 137 | 166 | 175 |
| FW1NBNE02DGTIG-1 | ga | 5 | 162 | 171 | 457 |
| FW1NBNE02C5T7R-1 | ga | 6 | 451 | 462 | 484 |
| FW1NBNE02EPN2P-1 | ga | 7 | 62 | 75 | 110 |
| FW1NBNE02EGOG1-1 | ga | 6 | 487 | 498 | 512 |
| FW1NBNE02C0PH4-1 | ga | 7 | 21 | 34 | 475 |
| FW1NBNE02C1673-1 | ga | 5 | 413 | 422 | 490 |
| FW1NBNE02D8PJH-1 | ga | 5 | 255 | 264 | 390 |
| FW1NBNE02DFM94-1 | ga | 5 | 420 | 429 | 503 |
| FW1NBNE02ES938-1 | ga | 5 | 136 | 145 | 384 |
| FW1NBNE02DEV60-1 | ga | 6 | 45 | 56 | 82 |
| FW1NBNE02DUGA0-1 | ga | 5 | 22 | 31 | 149 |
| FW1NBNE02EBGZ9-1 | ga | 5 | 92 | 101 | 443 |
| FW1NBNE02DZJDL-1 | ga | 6 | 480 | 491 | 522 |
| FW1NBNE02DZQ64-1 | ga | 5 | 141 | 150 | 490 |
| FW1NBNE02EOEM2-1 | ga | 5 | 59 | 68 | 76 |
| FW1NBNE02EI2JX-1 | ga | 6 | 310 | 321 | 495 |
| FW1NBNE02C1F91-1 | ga | 5 | 483 | 492 | 495 |
| FW1NBNE02DQM87-1 | ga | 5 | 2 | 11 | 463 |
| FW1NBNE02DFBCQ-1 | ga | 5 | 393 | 402 | 440 |
| FW1NBNE02D9S4V-1 | ga | 5 | 281 | 290 | 402 |
| FW1NBNE02DQZZ4-1 | ga | 6 | 365 | 376 | 446 |
| FW1NBNE02C2Q63-1 | ga | 5 | 55 | 64 | 487 |
| FW1NBNE02D8NWL-1 | ga | 5 | 38 | 47 | 403 |
| FW1NBNE02D7O1K-1 | ga | 8 | 36 | 51 | 149 |
| FW1NBNE02DT4HL-1 | ga | 5 | 41 | 50 | 215 |
| FW1NBNE02DPEP8-1 | ga | 7 | 35 | 48 | 415 |
| FW1NBNE02CZRGH-1 | ga | 11 | 507 | 528 | 548 |
| FW1NBNE02DSSER-1 | ga | 5 | 46 | 55 | 511 |
| FW1NBNE02DD21K-1 | ga | 12 | 478 | 501 | 503 |
| FW1NBNE02D9O63-1 | ga | 8 | 35 | 50 | 439 |
| FW1NBNE02C72XG-1 | ga | 6 | 5 | 16 | 174 |
| FW1NBNE02DDNB5-1 | ga | 5 | 1 | 10 | 117 |
| FW1NBNE02EH1AQ-1 | ga | 12 | 195 | 218 | 275 |
| FW1NBNE02D018T-1 | ga | 6 | 75 | 86 | 439 |
| FW1NBNE02DOV39-1 | ga | 5 | 141 | 150 | 224 |
| FW1NBNE02DKJRA-1 | ga | 14 | 116 | 143 | 200 |
| FW1NBNE02C5LQD-1 | ga | 9 | 372 | 389 | 435 |
| FW1NBNE02D4905-1 | ga | 10 | 44 | 63 | 91 |
| FW1NBNE02D45FA-1 | ga | 5 | 380 | 389 | 528 |
| FW1NBNE02DXM2U-1 | ga | 5 | 243 | 252 | 474 |
| FW1NBNE02D0YV7-1 | ga | 5 | 27 | 36 | 399 |
| FW1NBNE02EJDCM-1 | ga | 6 | 200 | 211 | 424 |
| FW1NBNE02DQS6T-1 | ga | 5 | 147 | 156 | 488 |
| FW1NBNE02D4SS9-1 | ga | 5 | 486 | 495 | 520 |
| FW1NBNE02D0V7K-1 | ga | 5 | 487 | 496 | 536 |
| FW1NBNE02EQM2I-1 | ga | 5 | 9 | 18 | 471 |
| FW1NBNE02EB4LZ-1 | ga | 5 | 47 | 56 | 489 |
| FW1NBNE02C7KG8-1 | ga | 9 | 478 | 495 | 504 |
| FW1NBNE02DSNS5-1 | ga | 6 | 181 | 192 | 466 |
| FW1NBNE02C42VJ-1 | ga | 5 | 19 | 28 | 100 |
| FW1NBNE02DQNNC-1 | ga | 10 | 17 | 36 | 55 |
| FW1NBNE02C9HH3-1 | ga | 5 | 210 | 219 | 537 |
| FW1NBNE02DARH1-1 | ga | 5 | 85 | 94 | 391 |
| FW1NBNE02DAK94-1 | ga | 6 | 395 | 406 | 412 |
| FW1NBNE02DOAJA-1 | ga | 7 | 39 | 52 | 453 |
| FW1NBNE02DX62P-1 | ga | 5 | 370 | 379 | 489 |
| FW1NBNE02ETQCE-1 | ga | 10 | 35 | 54 | 130 |
| FW1NBNE02DGVJ7-1 | ga | 5 | 323 | 332 | 515 |
| FW1NBNE02D0J0R-1 | ga | 6 | 66 | 77 | 149 |
| FW1NBNE02DTOFW-1 | ga | 5 | 453 | 462 | 516 |
| FW1NBNE02DX97C-1 | ga | 6 | 74 | 85 | 85 |
| FW1NBNE02DS0YZ-1 | ga | 5 | 323 | 332 | 496 |
| FW1NBNE02DIBOW-1 | ga | 8 | 13 | 28 | 257 |
| FW1NBNE02DAIOV-1 | ga | 7 | 230 | 243 | 364 |
| FW1NBNE02D5CU4-1 | ga | 6 | 44 | 55 | 453 |
| FW1NBNE02DAVJP-1 | ga | 5 | 6 | 15 | 482 |
| FW1NBNE02DSQIE-1 | ga | 6 | 54 | 65 | 485 |
| FW1NBNE02ER9NC-1 | ga | 7 | 52 | 65 | 314 |
| FW1NBNE02EG8FF-1 | ga | 5 | 328 | 337 | 393 |
| FW1NBNE02DSGPY-1 | ga | 5 | 26 | 35 | 371 |
| FW1NBNE02EFJYD-1 | ga | 5 | 23 | 32 | 388 |
| FW1NBNE02D9HTG-1 | ga | 6 | 3 | 14 | 279 |
| FW1NBNE02EW0E3-1 | ga | 7 | 137 | 150 | 556 |
| FW1NBNE02EO1RG-1 | ga | 5 | 37 | 46 | 510 |
| FW1NBNE02DWI0R-1 | ga | 5 | 442 | 451 | 536 |
| FW1NBNE02D2Z2E-1 | ga | 7 | 189 | 202 | 264 |
| FW1NBNE02DW3PJ-1 | ga | 5 | 217 | 226 | 525 |
| FW1NBNE02C01JO-1 | ga | 7 | 44 | 57 | 505 |
| FW1NBNE02D0SMU-1 | ga | 7 | 121 | 134 | 412 |
| FW1NBNE02DGZ7W-1 | ga | 5 | 26 | 35 | 470 |
| FW1NBNE02D168J-1 | ga | 5 | 83 | 92 | 514 |
| FW1NBNE02ECT7D-1 | ga | 5 | 111 | 120 | 395 |
| FW1NBNE02D105X-1 | ga | 5 | 63 | 72 | 393 |
| FW1NBNE02D9JBW-1 | ga | 6 | 56 | 67 | 483 |
| FW1NBNE02DNABB-1 | ga | 5 | 30 | 39 | 416 |
| FW1NBNE02EL1ZQ-1 | ga | 6 | 24 | 35 | 476 |
| FW1NBNE02C2DOJ-1 | ga | 5 | 271 | 280 | 463 |
| FW1NBNE02ETJXS-1 | ga | 5 | 393 | 402 | 479 |
| FW1NBNE02DGP9K-1 | ga | 13 | 45 | 70 | 140 |
| FW1NBNE02DEOWE-1 | ga | 6 | 505 | 516 | 554 |
| FW1NBNE02D48IG-1 | ga | 15 | 38 | 67 | 71 |
| FW1NBNE02D048F-1 | ga | 5 | 343 | 352 | 482 |
| FW1NBNE02DSOEJ-1 | ga | 5 | 22 | 31 | 407 |
| FW1NBNE02EOX82-1 | ga | 5 | 72 | 81 | 465 |
| FW1NBNE02D6AZH-1 | ga | 5 | 324 | 333 | 447 |
| FW1NBNE02EHKT0-1 | ga | 9 | 172 | 189 | 192 |
| FW1NBNE02DDM8F-1 | ga | 5 | 205 | 214 | 366 |
| FW1NBNE02DGCMW-1 | ga | 5 | 3 | 12 | 365 |
| FW1NBNE02C12KK-1 | ga | 5 | 184 | 193 | 509 |
| FW1NBNE02CZPFI-1 | ga | 5 | 427 | 436 | 471 |
| FW1NBNE02C3T1B-1 | ga | 6 | 25 | 36 | 289 |
| FW1NBNE02EHG2M-1 | ga | 5 | 18 | 27 | 472 |
| FW1NBNE02DC7LW-1 | ga | 5 | 95 | 104 | 243 |
| FW1NBNE02D7W04-1 | ga | 5 | 107 | 116 | 359 |
| FW1NBNE02D0GWD-1 | ga | 6 | 364 | 375 | 389 |
| FW1NBNE02D4GE2-1 | ga | 5 | 96 | 105 | 157 |
| FW1NBNE02ETQP6-1 | ga | 5 | 288 | 297 | 455 |
| contig00474-2 | gaa | 5 | 496 | 510 | 1207 |
| contig04273-2 | gaa | 5 | 274 | 288 | 364 |
| contig10468-3 | gaa | 7 | 727 | 747 | 986 |
| contig02350-1 | gaa | 5 | 210 | 224 | 534 |
| contig03066-1 | gaa | 5 | 470 | 484 | 647 |
| contig03701-1 | gaa | 5 | 243 | 257 | 540 |
| contig04243-1 | gaa | 5 | 922 | 936 | 1077 |
| contig04487-1 | gaa | 6 | 955 | 972 | 979 |
| contig05074-1 | gaa | 5 | 440 | 454 | 1147 |
| contig05437-1 | gaa | 5 | 8 | 22 | 782 |
| contig05574-1 | gaa | 5 | 741 | 755 | 943 |
| contig06927-1 | gaa | 8 | 59 | 82 | 1750 |
| contig10929-1 | gaa | 6 | 371 | 388 | 490 |
| contig12599-1 | gaa | 6 | 213 | 230 | 392 |
| contig12945-1 | gaa | 5 | 242 | 256 | 429 |
| FW1NBNE02EOUY0-1 | gaa | 5 | 99 | 113 | 340 |
| FW1NBNE02D3M1V-1 | gaa | 6 | 49 | 66 | 503 |
| FW1NBNE02DBGGC-1 | gaa | 5 | 419 | 433 | 448 |
| FW1NBNE02DUMF7-1 | gaa | 5 | 290 | 304 | 510 |
| FW1NBNE02D7HIQ-1 | gaa | 5 | 343 | 357 | 457 |
| FW1NBNE02EK2D2-1 | gaa | 5 | 310 | 324 | 404 |
| FW1NBNE02C1YD0-1 | gaa | 7 | 398 | 418 | 456 |
| FW1NBNE02EUCKW-1 | gaa | 7 | 370 | 390 | 464 |
| FW1NBNE02ET5MG-1 | gaa | 6 | 114 | 131 | 353 |
| FW1NBNE02C7B4W-1 | gaa | 5 | 378 | 392 | 394 |
| FW1NBNE02DYDXC-1 | gaa | 7 | 41 | 61 | 319 |
| FW1NBNE02DAS6U-1 | gaa | 7 | 42 | 62 | 78 |
| contig00372-1 | gaaa | 5 | 197 | 216 | 1757 |
| FW1NBNE02EU32P-1 | gaaa | 6 | 367 | 390 | 488 |
| contig00643-1 | gaag | 5 | 220 | 239 | 522 |
| FW1NBNE02C1YTP-1 | gaagag | 6 | 169 | 204 | 454 |
| contig01219-1 | gaatt | 6 | 438 | 467 | 485 |
| contig00971-2 | gac | 5 | 729 | 743 | 1329 |
| FW1NBNE02EURUW-2 | gac | 10 | 442 | 471 | 475 |
| contig01526-1 | gac | 8 | 142 | 165 | 1035 |
| contig02908-1 | gac | 6 | 376 | 393 | 1101 |
| contig10077-1 | gac | 6 | 139 | 156 | 400 |
| FW1NBNE02D85XI-1 | gac | 10 | 214 | 243 | 517 |
| FW1NBNE02C8SEK-1 | gac | 5 | 17 | 31 | 68 |
| contig05596-1 | gacaa | 5 | 1023 | 1047 | 1156 |
| contig11525-1 | gacag | 8 | 91 | 130 | 393 |
| FW1NBNE02CZPQ1-1 | gactgg | 5 | 69 | 98 | 263 |
| contig03811-2 | gag | 6 | 205 | 222 | 355 |
| FW1NBNE02DV9UC-2 | gag | 5 | 51 | 65 | 477 |
| FW1NBNE02D2Z2E-2 | gag | 5 | 211 | 225 | 264 |
| contig00659-3 | gag | 5 | 1476 | 1490 | 1696 |
| contig00290-1 | gag | 8 | 292 | 315 | 1312 |
| contig00505-1 | gag | 5 | 260 | 274 | 375 |
| contig00917-1 | gag | 9 | 424 | 450 | 555 |
| contig01557-1 | gag | 6 | 142 | 159 | 549 |
| contig02255-1 | gag | 5 | 343 | 357 | 1107 |
| contig04698-1 | gag | 5 | 17 | 31 | 496 |
| contig12620-1 | gag | 5 | 43 | 57 | 360 |
| contig12636-1 | gag | 7 | 260 | 280 | 402 |
| FW1NBNE02DINOW-1 | gag | 8 | 272 | 295 | 508 |
| FW1NBNE02DVE59-1 | gag | 5 | 258 | 272 | 296 |
| FW1NBNE02EIW9Z-1 | gag | 5 | 355 | 369 | 489 |
| FW1NBNE02C9MSV-1 | gag | 7 | 14 | 34 | 499 |
| FW1NBNE02DAHRZ-1 | gag | 5 | 197 | 211 | 400 |
| contig01417-1 | gagag | 5 | 818 | 842 | 1039 |
| FW1NBNE02CZ8VP-1 | gagc | 5 | 467 | 486 | 523 |
| contig00011-1 | gagg | 7 | 368 | 395 | 1278 |
| FW1NBNE02D579G-1 | gagg | 5 | 105 | 124 | 252 |
| contig04019-1 | gaggtg | 6 | 3 | 38 | 195 |
| contig04116-2 | gat | 5 | 295 | 309 | 320 |
| contig10585-4 | gat | 5 | 297 | 311 | 652 |
| contig01340-1 | gat | 6 | 586 | 603 | 676 |
| contig01355-1 | gat | 5 | 431 | 445 | 703 |
| contig04239-1 | gat | 7 | 884 | 904 | 1147 |
| contig04922-1 | gat | 6 | 694 | 711 | 734 |
| contig06380-1 | gat | 8 | 561 | 584 | 1009 |
| contig06802-1 | gat | 5 | 92 | 106 | 519 |
| contig06891-1 | gat | 5 | 28 | 42 | 345 |
| contig06965-1 | gat | 5 | 437 | 451 | 518 |
| contig08508-1 | gat | 5 | 318 | 332 | 536 |
| contig09151-1 | gat | 5 | 477 | 491 | 871 |
| contig10220-1 | gat | 5 | 130 | 144 | 1070 |
| contig10915-1 | gat | 5 | 97 | 111 | 386 |
| contig11076-1 | gat | 5 | 250 | 264 | 423 |
| contig12044-1 | gat | 6 | 256 | 273 | 360 |
| contig12217-1 | gat | 8 | 251 | 274 | 346 |
| contig13762-1 | gat | 6 | 100 | 117 | 322 |
| FW1NBNE02D5438-1 | gat | 8 | 130 | 153 | 242 |
| FW1NBNE02ENVU2-1 | gat | 5 | 136 | 150 | 240 |
| FW1NBNE02C6GEL-1 | gat | 5 | 199 | 213 | 480 |
| FW1NBNE02D4W1O-1 | gat | 7 | 278 | 298 | 502 |
| FW1NBNE02DBFLG-1 | gat | 6 | 94 | 111 | 435 |
| FW1NBNE02DLZKO-1 | gat | 5 | 172 | 186 | 234 |
| FW1NBNE02EQHYS-1 | gat | 5 | 153 | 167 | 371 |
| FW1NBNE02D4IAK-1 | gat | 5 | 164 | 178 | 256 |
| contig00595-1 | gata | 5 | 657 | 676 | 689 |
| contig09028-1 | gatata | 5 | 39 | 68 | 269 |
| contig01235-2 | gatct | 5 | 747 | 771 | 873 |
| contig06049-1 | gatg | 7 | 161 | 188 | 936 |
| FW1NBNE02EMQ78-2 | gatggt | 6 | 20 | 55 | 403 |
| FW1NBNE02D54LM-1 | gc | 5 | 414 | 423 | 428 |
| contig00952-2 | gca | 5 | 257 | 271 | 1869 |
| contig04916-2 | gca | 5 | 1209 | 1223 | 1772 |
| contig12906-2 | gca | 6 | 143 | 160 | 411 |
| FW1NBNE02D2XYX-2 | gca | 5 | 277 | 291 | 428 |
| FW1NBNE02C9ZIT-2 | gca | 5 | 94 | 108 | 373 |
| FW1NBNE02DYDXC-2 | gca | 5 | 90 | 104 | 319 |
| contig06949-3 | gca | 5 | 98 | 112 | 1522 |
| contig02691-1 | gca | 5 | 334 | 348 | 1206 |
| contig05658-1 | gca | 7 | 139 | 159 | 908 |
| contig07793-1 | gca | 5 | 93 | 107 | 1075 |
| contig12707-1 | gca | 6 | 216 | 233 | 259 |
| FW1NBNE02DG760-1 | gca | 5 | 1 | 15 | 524 |
| FW1NBNE02EQK14-1 | gca | 10 | 163 | 192 | 224 |
| FW1NBNE02DMXBZ-1 | gca | 10 | 313 | 342 | 366 |
| FW1NBNE02EYVC5-1 | gca | 6 | 102 | 119 | 322 |
| FW1NBNE02D8GDV-1 | gca | 7 | 41 | 61 | 124 |
| contig05667-3 | gcc | 5 | 1408 | 1422 | 1707 |
| contig08639-1 | gcc | 7 | 389 | 409 | 497 |
| contig11121-1 | gcc | 5 | 1232 | 1246 | 1518 |
| contig12676-1 | gcc | 6 | 246 | 263 | 395 |
| contig02547-1 | gcg | 6 | 421 | 438 | 523 |
| contig02776-1 | gcg | 6 | 1975 | 1992 | 2336 |
| contig06173-1 | gcg | 6 | 181 | 198 | 364 |
| FW1NBNE02EWBKI-1 | gcgag | 5 | 361 | 385 | 496 |
| contig01999-2 | gct | 5 | 1087 | 1101 | 1155 |
| contig12166-2 | gct | 5 | 172 | 186 | 416 |
| contig12886-2 | gct | 5 | 399 | 413 | 430 |
| contig05858-1 | gct | 5 | 97 | 111 | 1300 |
| contig05913-1 | gct | 7 | 436 | 456 | 567 |
| contig07254-1 | gct | 5 | 357 | 371 | 412 |
| contig07408-1 | gct | 5 | 919 | 933 | 937 |
| contig08078-1 | gct | 5 | 585 | 599 | 601 |
| contig09488-1 | gct | 5 | 190 | 204 | 496 |
| contig09904-1 | gct | 6 | 29 | 46 | 123 |
| contig10434-1 | gct | 8 | 410 | 433 | 643 |
| contig10863-1 | gct | 5 | 514 | 528 | 569 |
| FW1NBNE02D2DI3-1 | gct | 8 | 254 | 277 | 439 |
| FW1NBNE02C1GWP-1 | gct | 6 | 173 | 190 | 491 |
| FW1NBNE02EK2UT-1 | gct | 5 | 293 | 307 | 416 |
| FW1NBNE02EAB6Z-1 | gct | 6 | 61 | 78 | 105 |
| FW1NBNE02EDGZJ-1 | gct | 6 | 59 | 76 | 469 |
| FW1NBNE02EAI8S-1 | gct | 6 | 359 | 376 | 399 |
| FW1NBNE02DANKY-1 | gctg | 6 | 30 | 53 | 474 |
| contig04992-1 | gcttg | 7 | 86 | 120 | 511 |
| contig04027-2 | gga | 5 | 302 | 316 | 722 |
| FW1NBNE02DQ1YZ-2 | gga | 5 | 90 | 104 | 564 |
| FW1NBNE02D7EYC-3 | gga | 5 | 243 | 257 | 543 |
| contig06949-4 | gga | 7 | 486 | 506 | 1522 |
| contig00332-1 | gga | 6 | 72 | 89 | 2551 |
| contig03668-1 | gga | 5 | 717 | 731 | 789 |
| contig11906-1 | gga | 5 | 356 | 370 | 423 |
| FW1NBNE02EOY7J-1 | gga | 5 | 66 | 80 | 467 |
| FW1NBNE02EUG0E-1 | gga | 5 | 216 | 230 | 500 |
| FW1NBNE02C5GNS-2 | ggaaaa | 5 | 93 | 122 | 431 |
| FW1NBNE02EEJL1-2 | ggaaaa | 5 | 92 | 121 | 499 |
| contig06106-1 | ggaaaa | 5 | 163 | 192 | 1159 |
| contig08770-1 | ggag | 6 | 173 | 196 | 488 |
| contig11354-1 | ggagca | 6 | 174 | 209 | 1060 |
| contig01174-1 | ggaggc | 5 | 1266 | 1295 | 1515 |
| contig00493-1 | ggc | 7 | 1022 | 1042 | 1186 |
| contig01373-1 | ggc | 6 | 226 | 243 | 909 |
| contig04376-1 | ggc | 6 | 326 | 343 | 619 |
| contig05787-1 | ggc | 5 | 32 | 46 | 685 |
| FW1NBNE02EL8UB-1 | ggc | 7 | 450 | 470 | 476 |
| FW1NBNE02ELDEB-1 | ggc | 5 | 328 | 342 | 480 |
| contig01176-1 | ggcc | 5 | 348 | 367 | 467 |
| contig10622-1 | ggct | 5 | 329 | 348 | 481 |
| FW1NBNE02DZ152-1 | ggct | 6 | 194 | 217 | 505 |
| contig12636-2 | ggt | 5 | 304 | 318 | 402 |
| contig10624-3 | ggt | 6 | 231 | 248 | 367 |
| contig00050-1 | ggt | 5 | 462 | 476 | 556 |
| contig02391-1 | ggt | 5 | 337 | 351 | 609 |
| contig06796-1 | ggt | 5 | 440 | 454 | 1019 |
| FW1NBNE02DKKX7-1 | ggt | 5 | 115 | 129 | 230 |
| FW1NBNE02ETCVK-1 | ggt | 6 | 50 | 67 | 72 |
| FW1NBNE02DQ1YZ-1 | ggt | 6 | 72 | 89 | 564 |
| FW1NBNE02C8CMB-1 | ggt | 5 | 61 | 75 | 150 |
| FW1NBNE02DTW7L-1 | ggt | 5 | 357 | 371 | 410 |
| contig06377-2 | ggtcga | 5 | 240 | 269 | 720 |
| FW1NBNE02DK9GT-1 | ggtggc | 5 | 308 | 337 | 508 |
| FW1NBNE02DGDL3-1 | ggttca | 6 | 183 | 218 | 411 |
| contig13632-2 | gt | 34 | 37 | 104 | 104 |
| FW1NBNE02DUD8I-2 | gt | 9 | 53 | 70 | 85 |
| FW1NBNE02DFH8Z-2 | gt | 6 | 266 | 277 | 329 |
| FW1NBNE02DEZD5-2 | gt | 5 | 106 | 115 | 142 |
| FW1NBNE02D8LYW-2 | gt | 5 | 465 | 474 | 492 |
| FW1NBNE02EOR8E-2 | gt | 6 | 44 | 55 | 97 |
| FW1NBNE02C107Z-2 | gt | 9 | 51 | 68 | 142 |
| FW1NBNE02EMS7G-2 | gt | 18 | 236 | 271 | 271 |
| contig10585-3 | gt | 5 | 562 | 571 | 652 |
| contig00174-1 | gt | 5 | 92 | 101 | 119 |
| contig00334-1 | gt | 5 | 986 | 995 | 3497 |
| contig00530-1 | gt | 5 | 1661 | 1670 | 1671 |
| contig00562-1 | gt | 5 | 2097 | 2106 | 2132 |
| contig01925-1 | gt | 5 | 645 | 654 | 683 |
| contig02312-1 | gt | 6 | 1292 | 1303 | 1315 |
| contig02747-1 | gt | 8 | 1009 | 1024 | 1068 |
| contig03206-1 | gt | 5 | 831 | 840 | 983 |
| contig04914-1 | gt | 5 | 40 | 49 | 808 |
| contig08392-1 | gt | 5 | 537 | 546 | 667 |
| contig09853-1 | gt | 5 | 369 | 378 | 399 |
| contig10789-1 | gt | 5 | 80 | 89 | 487 |
| contig13002-1 | gt | 6 | 89 | 100 | 586 |
| FW1NBNE02C8POD-1 | gt | 8 | 162 | 177 | 214 |
| FW1NBNE02EB731-1 | gt | 10 | 151 | 170 | 220 |
| FW1NBNE02D4TDX-1 | gt | 5 | 453 | 462 | 514 |
| FW1NBNE02DA40K-1 | gt | 7 | 383 | 396 | 487 |
| FW1NBNE02C3RBT-1 | gt | 8 | 158 | 173 | 209 |
| FW1NBNE02EOR8E-1 | gt | 7 | 28 | 41 | 97 |
| FW1NBNE02EQXE8-1 | gt | 5 | 366 | 375 | 436 |
| FW1NBNE02DOWZR-1 | gt | 12 | 443 | 466 | 487 |
| contig00659-1 | gta | 8 | 1076 | 1099 | 1696 |
| contig04287-1 | gta | 5 | 711 | 725 | 1017 |
| FW1NBNE02C8RGY-1 | gta | 5 | 283 | 297 | 347 |
| FW1NBNE02DX3SQ-2 | gtc | 5 | 206 | 220 | 279 |
| contig09169-1 | gtc | 6 | 23 | 40 | 159 |
| FW1NBNE02DA773-1 | gtc | 5 | 171 | 185 | 457 |
| contig11776-1 | gtcatg | 7 | 306 | 347 | 478 |
| contig09699-1 | gtct | 9 | 727 | 762 | 841 |
| contig03911-1 | gtg | 7 | 594 | 614 | 873 |
| contig05721-1 | gtg | 7 | 425 | 445 | 1029 |
| contig07485-1 | gtg | 5 | 295 | 309 | 499 |
| FW1NBNE02D1ETU-1 | gtg | 5 | 290 | 304 | 471 |
| FW1NBNE02C2QKU-1 | gtg | 5 | 283 | 297 | 361 |
| FW1NBNE02EUPGM-1 | gtg | 8 | 69 | 92 | 503 |
| FW1NBNE02DGDH0-1 | gtga | 5 | 450 | 469 | 485 |
| contig00066-1 | gtt | 5 | 110 | 124 | 305 |
| contig02638-1 | gtt | 6 | 170 | 187 | 539 |
| contig03700-1 | gtt | 5 | 816 | 830 | 830 |
| FW1NBNE02DTTAG-1 | gtt | 5 | 290 | 304 | 540 |
| FW1NBNE02C1WGM-1 | gttgg | 5 | 185 | 209 | 464 |
| contig10863-2 | gttt | 5 | 480 | 499 | 569 |
| contig01098-2 | ta | 5 | 225 | 234 | 520 |
| contig02288-2 | ta | 5 | 341 | 350 | 458 |
| contig02902-2 | ta | 5 | 190 | 199 | 1348 |
| contig07379-2 | ta | 7 | 807 | 820 | 911 |
| contig08828-2 | ta | 7 | 206 | 219 | 670 |
| contig09502-2 | ta | 5 | 409 | 418 | 460 |
| contig09668-2 | ta | 8 | 896 | 911 | 957 |
| contig10585-2 | ta | 6 | 425 | 436 | 652 |
| contig12570-2 | ta | 5 | 483 | 492 | 622 |
| contig12829-2 | ta | 12 | 94 | 117 | 458 |
| contig13002-2 | ta | 10 | 100 | 119 | 586 |
| contig13188-2 | ta | 5 | 223 | 232 | 265 |
| FW1NBNE02DEQL5-2 | ta | 15 | 178 | 207 | 221 |
| FW1NBNE02C8POD-2 | ta | 6 | 177 | 188 | 214 |
| FW1NBNE02DL5CE-2 | ta | 5 | 118 | 127 | 225 |
| FW1NBNE02D2C78-2 | ta | 5 | 483 | 492 | 492 |
| FW1NBNE02D58MH-2 | ta | 100 | 45 | 244 | 410 |
| FW1NBNE02C521M-2 | ta | 10 | 107 | 126 | 224 |
| FW1NBNE02DZ7W7-2 | ta | 5 | 137 | 146 | 326 |
| FW1NBNE02DVLU7-2 | ta | 5 | 121 | 130 | 318 |
| FW1NBNE02C3RBT-2 | ta | 5 | 173 | 182 | 209 |
| FW1NBNE02C1ZLL-2 | ta | 8 | 415 | 430 | 493 |
| FW1NBNE02EW71D-2 | ta | 8 | 104 | 119 | 183 |
| FW1NBNE02C8CU1-2 | ta | 10 | 55 | 74 | 93 |
| FW1NBNE02C5TD5-2 | ta | 7 | 104 | 117 | 267 |
| FW1NBNE02DA7WZ-2 | ta | 6 | 333 | 344 | 345 |
| FW1NBNE02D2NXX-2 | ta | 5 | 161 | 170 | 388 |
| FW1NBNE02DS73D-2 | ta | 8 | 303 | 318 | 364 |
| FW1NBNE02DW9W9-2 | ta | 8 | 379 | 394 | 411 |
| FW1NBNE02C4AF4-2 | ta | 5 | 377 | 386 | 493 |
| FW1NBNE02DV5AL-2 | ta | 7 | 407 | 420 | 466 |
| FW1NBNE02EYG8R-2 | ta | 5 | 377 | 386 | 458 |
| FW1NBNE02ED46X-2 | ta | 5 | 23 | 32 | 296 |
| FW1NBNE02ELBE3-2 | ta | 8 | 138 | 153 | 274 |
| FW1NBNE02EWO1K-2 | ta | 9 | 275 | 292 | 329 |
| FW1NBNE02C1SR8-2 | ta | 11 | 367 | 388 | 401 |
| FW1NBNE02EJYAI-2 | ta | 8 | 26 | 41 | 90 |
| FW1NBNE02C3KQJ-2 | ta | 5 | 175 | 184 | 344 |
| FW1NBNE02EMCQD-2 | ta | 9 | 107 | 124 | 195 |
| FW1NBNE02ESN5E-2 | ta | 6 | 159 | 170 | 257 |
| FW1NBNE02DFH8Z-3 | ta | 8 | 277 | 292 | 329 |
| FW1NBNE02D58MH-3 | ta | 80 | 251 | 410 | 410 |
| FW1NBNE02D8LYW-3 | ta | 8 | 474 | 489 | 492 |
| FW1NBNE02DS73D-3 | ta | 8 | 343 | 358 | 364 |
| FW1NBNE02C29CZ-3 | ta | 7 | 262 | 275 | 369 |
| FW1NBNE02EGFRF-3 | ta | 5 | 260 | 269 | 349 |
| FW1NBNE02ESBZR-3 | ta | 13 | 96 | 121 | 130 |
| FW1NBNE02EGFRF-4 | ta | 5 | 272 | 281 | 349 |
| contig00005-1 | ta | 5 | 2661 | 2670 | 3184 |
| contig00137-1 | ta | 5 | 66 | 75 | 561 |
| contig00147-1 | ta | 5 | 18 | 27 | 1101 |
| contig00175-1 | ta | 5 | 51 | 60 | 791 |
| contig00248-1 | ta | 14 | 24 | 51 | 1085 |
| contig00378-1 | ta | 7 | 226 | 239 | 293 |
| contig00419-1 | ta | 5 | 330 | 339 | 669 |
| contig00613-1 | ta | 8 | 28 | 43 | 1516 |
| contig00633-1 | ta | 5 | 206 | 215 | 585 |
| contig00828-1 | ta | 5 | 22 | 31 | 1965 |
| contig00911-1 | ta | 6 | 87 | 98 | 1236 |
| contig00912-1 | ta | 5 | 16 | 25 | 538 |
| contig01072-1 | ta | 5 | 9 | 18 | 336 |
| contig01077-1 | ta | 6 | 48 | 59 | 394 |
| contig01098-1 | ta | 5 | 213 | 222 | 520 |
| contig01133-1 | ta | 5 | 89 | 98 | 1593 |
| contig01239-1 | ta | 7 | 1550 | 1563 | 1734 |
| contig01244-1 | ta | 5 | 180 | 189 | 398 |
| contig01375-1 | ta | 7 | 150 | 163 | 184 |
| contig01399-1 | ta | 5 | 505 | 514 | 542 |
| contig01495-1 | ta | 5 | 1036 | 1045 | 1079 |
| contig01749-1 | ta | 7 | 1083 | 1096 | 1208 |
| contig01845-1 | ta | 5 | 229 | 238 | 532 |
| contig01852-1 | ta | 5 | 17 | 26 | 1752 |
| contig02153-1 | ta | 6 | 876 | 887 | 961 |
| contig02188-1 | ta | 5 | 281 | 290 | 517 |
| contig02520-1 | ta | 6 | 550 | 561 | 579 |
| contig02689-1 | ta | 8 | 185 | 200 | 600 |
| contig02800-1 | ta | 8 | 310 | 325 | 526 |
| contig02956-1 | ta | 6 | 2348 | 2359 | 2409 |
| contig02969-1 | ta | 5 | 310 | 319 | 749 |
| contig03060-1 | ta | 5 | 1173 | 1182 | 1196 |
| contig03211-1 | ta | 5 | 1 | 10 | 300 |
| contig03223-1 | ta | 5 | 357 | 366 | 682 |
| contig03305-1 | ta | 5 | 387 | 396 | 452 |
| contig03325-1 | ta | 9 | 603 | 620 | 859 |
| contig03485-1 | ta | 5 | 452 | 461 | 701 |
| contig03672-1 | ta | 5 | 8 | 17 | 443 |
| contig03746-1 | ta | 6 | 250 | 261 | 787 |
| contig03760-1 | ta | 5 | 1176 | 1185 | 1205 |
| contig04106-1 | ta | 12 | 102 | 125 | 1130 |
| contig04215-1 | ta | 5 | 701 | 710 | 765 |
| contig04439-1 | ta | 5 | 242 | 251 | 522 |
| contig04447-1 | ta | 5 | 1494 | 1503 | 1647 |
| contig04552-1 | ta | 5 | 1709 | 1718 | 1753 |
| contig04746-1 | ta | 7 | 763 | 776 | 783 |
| contig04827-1 | ta | 6 | 103 | 114 | 132 |
| contig04840-1 | ta | 5 | 607 | 616 | 670 |
| contig04862-1 | ta | 5 | 465 | 474 | 587 |
| contig04902-1 | ta | 6 | 905 | 916 | 930 |
| contig04980-1 | ta | 9 | 1407 | 1424 | 1733 |
| contig04981-1 | ta | 10 | 1071 | 1090 | 1152 |
| contig05049-1 | ta | 7 | 512 | 525 | 657 |
| contig05307-1 | ta | 6 | 290 | 301 | 646 |
| contig05396-1 | ta | 5 | 79 | 88 | 520 |
| contig05481-1 | ta | 5 | 1 | 10 | 613 |
| contig05520-1 | ta | 5 | 135 | 144 | 384 |
| contig05558-1 | ta | 5 | 1134 | 1143 | 1157 |
| contig05603-1 | ta | 8 | 1 | 16 | 1140 |
| contig06057-1 | ta | 8 | 419 | 434 | 497 |
| contig06233-1 | ta | 5 | 35 | 44 | 797 |
| contig06338-1 | ta | 9 | 698 | 715 | 856 |
| contig06377-1 | ta | 5 | 91 | 100 | 720 |
| contig06448-1 | ta | 9 | 540 | 557 | 606 |
| contig06698-1 | ta | 5 | 392 | 401 | 478 |
| contig06733-1 | ta | 7 | 1 | 14 | 974 |
| contig06888-1 | ta | 5 | 623 | 632 | 779 |
| contig06903-1 | ta | 5 | 86 | 95 | 1958 |
| contig07141-1 | ta | 5 | 66 | 75 | 215 |
| contig07199-1 | ta | 5 | 1188 | 1197 | 1199 |
| contig07224-1 | ta | 6 | 560 | 571 | 589 |
| contig07286-1 | ta | 5 | 322 | 331 | 395 |
| contig07379-1 | ta | 6 | 793 | 804 | 911 |
| contig07456-1 | ta | 6 | 10 | 21 | 168 |
| contig07644-1 | ta | 5 | 127 | 136 | 624 |
| contig07654-1 | ta | 5 | 55 | 64 | 569 |
| contig08196-1 | ta | 5 | 47 | 56 | 1362 |
| contig08758-1 | ta | 5 | 306 | 315 | 368 |
| contig08787-1 | ta | 8 | 114 | 129 | 595 |
| contig09013-1 | ta | 5 | 261 | 270 | 560 |
| contig09015-1 | ta | 5 | 64 | 73 | 487 |
| contig09021-1 | ta | 6 | 158 | 169 | 547 |
| contig09245-1 | ta | 7 | 141 | 154 | 392 |
| contig09393-1 | ta | 5 | 115 | 124 | 231 |
| contig09485-1 | ta | 7 | 156 | 169 | 581 |
| contig09518-1 | ta | 5 | 697 | 706 | 831 |
| contig09668-1 | ta | 5 | 872 | 881 | 957 |
| contig09738-1 | ta | 21 | 1042 | 1083 | 1084 |
| contig10614-1 | ta | 7 | 476 | 489 | 490 |
| contig10945-1 | ta | 5 | 22 | 31 | 556 |
| contig11317-1 | ta | 5 | 171 | 180 | 493 |
| contig11345-1 | ta | 5 | 63 | 72 | 483 |
| contig11754-1 | ta | 5 | 425 | 434 | 472 |
| contig11919-1 | ta | 13 | 1 | 26 | 442 |
| contig11983-1 | ta | 6 | 95 | 106 | 473 |
| contig12033-1 | ta | 7 | 434 | 447 | 628 |
| contig12090-1 | ta | 7 | 1 | 14 | 512 |
| contig12246-1 | ta | 5 | 133 | 142 | 254 |
| contig12291-1 | ta | 6 | 73 | 84 | 122 |
| contig12304-1 | ta | 5 | 4 | 13 | 425 |
| contig12592-1 | ta | 8 | 1 | 16 | 378 |
| contig12750-1 | ta | 7 | 198 | 211 | 368 |
| contig12765-1 | ta | 8 | 349 | 364 | 508 |
| contig12772-1 | ta | 7 | 110 | 123 | 424 |
| contig12946-1 | ta | 5 | 147 | 156 | 304 |
| contig12953-1 | ta | 8 | 222 | 237 | 405 |
| contig13128-1 | ta | 5 | 230 | 239 | 280 |
| contig13226-1 | ta | 7 | 51 | 64 | 316 |
| contig13449-1 | ta | 5 | 944 | 953 | 970 |
| contig13518-1 | ta | 5 | 90 | 99 | 190 |
| contig13525-1 | ta | 14 | 389 | 416 | 419 |
| contig13723-1 | ta | 6 | 732 | 743 | 898 |
| contig13785-1 | ta | 6 | 2555 | 2566 | 2611 |
| contig14002-1 | ta | 19 | 83 | 120 | 128 |
| FW1NBNE02D75VK-1 | ta | 5 | 185 | 194 | 286 |
| FW1NBNE02EXI97-1 | ta | 9 | 33 | 50 | 50 |
| FW1NBNE02D2PU7-1 | ta | 8 | 42 | 57 | 57 |
| FW1NBNE02DHA7I-1 | ta | 10 | 40 | 59 | 60 |
| FW1NBNE02EFKPK-1 | ta | 11 | 103 | 124 | 124 |
| FW1NBNE02C7QQ5-1 | ta | 8 | 71 | 86 | 86 |
| FW1NBNE02D9LJ2-1 | ta | 17 | 36 | 69 | 70 |
| FW1NBNE02C5D3W-1 | ta | 8 | 46 | 61 | 104 |
| FW1NBNE02EN3QL-1 | ta | 9 | 7 | 24 | 55 |
| FW1NBNE02EKMU8-1 | ta | 32 | 25 | 88 | 88 |
| FW1NBNE02DR8UX-1 | ta | 5 | 1 | 10 | 66 |
| FW1NBNE02DB1RL-1 | ta | 24 | 29 | 76 | 76 |
| FW1NBNE02C27A4-1 | ta | 7 | 77 | 90 | 533 |
| FW1NBNE02EMQL6-1 | ta | 11 | 65 | 86 | 99 |
| FW1NBNE02D0JP5-1 | ta | 8 | 103 | 118 | 118 |
| FW1NBNE02DITLQ-1 | ta | 47 | 52 | 145 | 145 |
| FW1NBNE02CZBLT-1 | ta | 6 | 3 | 14 | 62 |
| FW1NBNE02EIAOZ-1 | ta | 11 | 42 | 63 | 63 |
| FW1NBNE02DNLSJ-1 | ta | 10 | 37 | 56 | 414 |
| FW1NBNE02DJVQ4-1 | ta | 6 | 133 | 144 | 537 |
| FW1NBNE02ENBY4-1 | ta | 15 | 31 | 60 | 61 |
| FW1NBNE02DMURA-1 | ta | 8 | 55 | 70 | 70 |
| FW1NBNE02EGPAM-1 | ta | 8 | 22 | 37 | 138 |
| FW1NBNE02EMUSQ-1 | ta | 58 | 66 | 181 | 181 |
| FW1NBNE02DMNU1-1 | ta | 16 | 74 | 105 | 105 |
| FW1NBNE02D00H4-1 | ta | 25 | 93 | 142 | 142 |
| FW1NBNE02DTYHS-1 | ta | 10 | 56 | 75 | 75 |
| FW1NBNE02DS71A-1 | ta | 5 | 50 | 59 | 60 |
| FW1NBNE02DBY41-1 | ta | 11 | 113 | 134 | 134 |
| FW1NBNE02DBPIE-1 | ta | 14 | 35 | 62 | 63 |
| FW1NBNE02DP4YV-1 | ta | 26 | 80 | 131 | 131 |
| FW1NBNE02D84SX-1 | ta | 57 | 108 | 221 | 221 |
| FW1NBNE02DY3TL-1 | ta | 9 | 39 | 56 | 56 |
| FW1NBNE02DDPNE-1 | ta | 5 | 273 | 282 | 373 |
| FW1NBNE02C4GB0-1 | ta | 6 | 7 | 18 | 64 |
| FW1NBNE02D8P8H-1 | ta | 8 | 71 | 86 | 86 |
| FW1NBNE02EXT05-1 | ta | 12 | 101 | 124 | 275 |
| FW1NBNE02DPFQP-1 | ta | 17 | 422 | 455 | 455 |
| FW1NBNE02DPOGA-1 | ta | 15 | 78 | 107 | 146 |
| FW1NBNE02EDMQ3-1 | ta | 6 | 238 | 249 | 406 |
| FW1NBNE02C4BGX-1 | ta | 11 | 41 | 62 | 63 |
| FW1NBNE02C2VSH-1 | ta | 9 | 35 | 52 | 52 |
| FW1NBNE02ETG1P-1 | ta | 7 | 58 | 71 | 71 |
| FW1NBNE02DIT4Q-1 | ta | 13 | 68 | 93 | 97 |
| FW1NBNE02DC9XN-1 | ta | 6 | 111 | 122 | 448 |
| FW1NBNE02DLX6W-1 | ta | 6 | 57 | 68 | 68 |
| FW1NBNE02DX2KQ-1 | ta | 5 | 308 | 317 | 383 |
| FW1NBNE02DANIX-1 | ta | 7 | 505 | 518 | 522 |
| FW1NBNE02D7EYC-1 | ta | 10 | 490 | 509 | 543 |
| FW1NBNE02EFHAU-1 | ta | 7 | 294 | 307 | 464 |
| FW1NBNE02DI0GE-1 | ta | 5 | 32 | 41 | 138 |
| FW1NBNE02C9B13-1 | ta | 15 | 80 | 109 | 113 |
| FW1NBNE02EVVT2-1 | ta | 5 | 62 | 71 | 75 |
| FW1NBNE02C9FP7-1 | ta | 8 | 55 | 70 | 70 |
| FW1NBNE02DR952-1 | ta | 198 | 1 | 396 | 396 |
| FW1NBNE02D72O7-1 | ta | 10 | 266 | 285 | 335 |
| FW1NBNE02C080M-1 | ta | 9 | 23 | 40 | 252 |
| FW1NBNE02DYQGG-1 | ta | 8 | 456 | 471 | 472 |
| FW1NBNE02C2B1L-1 | ta | 5 | 29 | 38 | 266 |
| FW1NBNE02C8JI0-1 | ta | 9 | 302 | 319 | 469 |
| FW1NBNE02D58MH-1 | ta | 6 | 20 | 31 | 410 |
| FW1NBNE02C9UOE-1 | ta | 16 | 42 | 73 | 73 |
| FW1NBNE02ERMSH-1 | ta | 5 | 41 | 50 | 50 |
| FW1NBNE02DH8ZX-1 | ta | 5 | 252 | 261 | 358 |
| FW1NBNE02D8GQV-1 | ta | 10 | 91 | 110 | 412 |
| FW1NBNE02D7E0S-1 | ta | 7 | 202 | 215 | 388 |
| FW1NBNE02DBVJW-1 | ta | 5 | 8 | 17 | 510 |
| FW1NBNE02DLDQR-1 | ta | 13 | 246 | 271 | 316 |
| FW1NBNE02D3G6I-1 | ta | 16 | 72 | 103 | 103 |
| FW1NBNE02DS2UO-1 | ta | 7 | 66 | 79 | 79 |
| FW1NBNE02CZUT9-1 | ta | 14 | 101 | 128 | 154 |
| FW1NBNE02DE24P-1 | ta | 64 | 119 | 246 | 246 |
| FW1NBNE02DEP8O-1 | ta | 6 | 50 | 61 | 62 |
| FW1NBNE02C1E8K-1 | ta | 175 | 66 | 415 | 415 |
| FW1NBNE02EAE1E-1 | ta | 7 | 367 | 380 | 416 |
| FW1NBNE02EU7U5-1 | ta | 7 | 126 | 139 | 139 |
| FW1NBNE02DBTMC-1 | ta | 6 | 12 | 23 | 74 |
| FW1NBNE02DOMJR-1 | ta | 7 | 507 | 520 | 528 |
| FW1NBNE02D5RS9-1 | ta | 5 | 312 | 321 | 482 |
| FW1NBNE02C8DAI-1 | ta | 198 | 1 | 396 | 396 |
| FW1NBNE02DSBFD-1 | ta | 11 | 204 | 225 | 241 |
| FW1NBNE02DAOTC-1 | ta | 5 | 80 | 89 | 461 |
| FW1NBNE02EXPY3-1 | ta | 12 | 147 | 170 | 355 |
| FW1NBNE02EQW9B-1 | ta | 5 | 308 | 317 | 385 |
| FW1NBNE02EW7LL-1 | ta | 5 | 133 | 142 | 431 |
| FW1NBNE02DC1AQ-1 | ta | 5 | 402 | 411 | 469 |
| FW1NBNE02D2YCP-1 | ta | 7 | 429 | 442 | 449 |
| FW1NBNE02ESZ0O-1 | ta | 8 | 19 | 34 | 72 |
| FW1NBNE02EJLH6-1 | ta | 16 | 23 | 54 | 54 |
| FW1NBNE02DLHYH-1 | ta | 5 | 81 | 90 | 278 |
| FW1NBNE02DAC1Q-1 | ta | 5 | 201 | 210 | 516 |
| FW1NBNE02ECX32-1 | ta | 8 | 457 | 472 | 474 |
| FW1NBNE02DM35J-1 | ta | 5 | 90 | 99 | 513 |
| FW1NBNE02EH3NV-1 | ta | 10 | 526 | 545 | 545 |
| FW1NBNE02D8P0K-1 | ta | 17 | 66 | 99 | 103 |
| FW1NBNE02DWIBB-1 | ta | 27 | 1 | 54 | 54 |
| FW1NBNE02EJB48-1 | ta | 7 | 53 | 66 | 513 |
| FW1NBNE02EJDCV-1 | ta | 5 | 133 | 142 | 507 |
| FW1NBNE02C05E4-1 | ta | 7 | 406 | 419 | 456 |
| FW1NBNE02C3ENL-1 | ta | 7 | 128 | 141 | 300 |
| FW1NBNE02ETUPO-1 | ta | 5 | 294 | 303 | 402 |
| FW1NBNE02EX8KO-1 | ta | 8 | 42 | 57 | 88 |
| FW1NBNE02EU52R-1 | ta | 10 | 51 | 70 | 70 |
| FW1NBNE02DRPT1-1 | ta | 5 | 108 | 117 | 515 |
| FW1NBNE02D21UP-1 | ta | 6 | 447 | 458 | 525 |
| FW1NBNE02DYLMV-1 | ta | 5 | 291 | 300 | 500 |
| FW1NBNE02EMDC3-1 | ta | 6 | 506 | 517 | 517 |
| FW1NBNE02DSUPT-1 | ta | 5 | 64 | 73 | 477 |
| FW1NBNE02DQM8V-1 | ta | 6 | 168 | 179 | 191 |
| FW1NBNE02DA7WZ-1 | ta | 38 | 248 | 323 | 345 |
| FW1NBNE02EB4OT-1 | ta | 5 | 67 | 76 | 76 |
| FW1NBNE02EJE43-1 | ta | 7 | 313 | 326 | 362 |
| FW1NBNE02ECKG5-1 | ta | 10 | 447 | 466 | 466 |
| FW1NBNE02DDNTH-1 | ta | 9 | 10 | 27 | 75 |
| FW1NBNE02EEDRO-1 | ta | 10 | 41 | 60 | 69 |
| FW1NBNE02C0BJR-1 | ta | 15 | 68 | 97 | 120 |
| FW1NBNE02EVULX-1 | ta | 9 | 58 | 75 | 75 |
| FW1NBNE02C8URL-1 | ta | 12 | 18 | 41 | 328 |
| FW1NBNE02DA6MH-1 | ta | 5 | 448 | 457 | 501 |
| FW1NBNE02DX228-1 | ta | 198 | 1 | 396 | 396 |
| FW1NBNE02EOE7O-1 | ta | 198 | 1 | 396 | 396 |
| FW1NBNE02DERGM-1 | ta | 10 | 156 | 175 | 386 |
| FW1NBNE02EL5XE-1 | ta | 5 | 11 | 20 | 157 |
| FW1NBNE02DOP26-1 | ta | 13 | 147 | 172 | 188 |
| FW1NBNE02DXPPT-1 | ta | 8 | 78 | 93 | 157 |
| FW1NBNE02EXQ2E-1 | ta | 6 | 53 | 64 | 65 |
| FW1NBNE02DY1KU-1 | ta | 6 | 372 | 383 | 450 |
| FW1NBNE02DEMF5-1 | ta | 12 | 30 | 53 | 53 |
| FW1NBNE02EMUUN-1 | ta | 14 | 144 | 171 | 234 |
| FW1NBNE02C1WJ8-1 | ta | 5 | 91 | 100 | 228 |
| FW1NBNE02DAPXO-1 | ta | 7 | 128 | 141 | 267 |
| FW1NBNE02EIN06-1 | ta | 8 | 52 | 67 | 316 |
| FW1NBNE02DETMB-1 | ta | 5 | 185 | 194 | 485 |
| FW1NBNE02C4AF4-1 | ta | 5 | 313 | 322 | 493 |
| FW1NBNE02DA2II-1 | ta | 9 | 150 | 167 | 365 |
| FW1NBNE02EICH0-1 | ta | 5 | 433 | 442 | 513 |
| FW1NBNE02DAUVI-1 | ta | 6 | 1 | 12 | 168 |
| FW1NBNE02EWYIH-1 | ta | 5 | 10 | 19 | 480 |
| FW1NBNE02DP3H1-1 | ta | 7 | 17 | 30 | 67 |
| FW1NBNE02D8ZMN-1 | ta | 6 | 74 | 85 | 400 |
| FW1NBNE02CZKHT-1 | ta | 9 | 24 | 41 | 238 |
| FW1NBNE02C8P97-1 | ta | 8 | 34 | 49 | 387 |
| FW1NBNE02D35SM-1 | ta | 5 | 29 | 38 | 541 |
| FW1NBNE02EWFEV-1 | ta | 10 | 41 | 60 | 63 |
| FW1NBNE02C5NGN-1 | ta | 59 | 1 | 118 | 119 |
| FW1NBNE02ED46X-1 | ta | 5 | 4 | 13 | 296 |
| FW1NBNE02D81N6-1 | ta | 6 | 543 | 554 | 555 |
| FW1NBNE02DVQTQ-1 | ta | 17 | 260 | 293 | 306 |
| FW1NBNE02D30RA-1 | ta | 6 | 143 | 154 | 366 |
| FW1NBNE02DVFKZ-1 | ta | 7 | 218 | 231 | 454 |
| FW1NBNE02ENM0R-1 | ta | 11 | 96 | 117 | 134 |
| FW1NBNE02C2Z5K-1 | ta | 5 | 180 | 189 | 509 |
| FW1NBNE02EWO1K-1 | ta | 6 | 24 | 35 | 329 |
| FW1NBNE02D9OHG-1 | ta | 13 | 300 | 325 | 329 |
| FW1NBNE02EZJ6E-1 | ta | 5 | 293 | 302 | 494 |
| FW1NBNE02DI46F-1 | ta | 5 | 21 | 30 | 436 |
| FW1NBNE02EISKT-1 | ta | 15 | 241 | 270 | 358 |
| FW1NBNE02D77UE-1 | ta | 6 | 172 | 183 | 519 |
| FW1NBNE02EMQWB-1 | ta | 5 | 105 | 114 | 516 |
| FW1NBNE02DGMNI-1 | ta | 26 | 1 | 52 | 57 |
| FW1NBNE02EAY62-1 | ta | 8 | 203 | 218 | 454 |
| FW1NBNE02DBDF0-1 | ta | 5 | 13 | 22 | 367 |
| FW1NBNE02EW2QG-1 | ta | 12 | 431 | 454 | 454 |
| FW1NBNE02EUL5K-1 | ta | 6 | 132 | 143 | 145 |
| FW1NBNE02EMS7G-1 | ta | 5 | 150 | 159 | 271 |
| FW1NBNE02C9CCA-1 | ta | 7 | 106 | 119 | 251 |
| FW1NBNE02D1ZVK-1 | ta | 5 | 186 | 195 | 374 |
| FW1NBNE02ED74G-1 | ta | 5 | 132 | 141 | 308 |
| FW1NBNE02DBI6A-1 | ta | 5 | 5 | 14 | 79 |
| FW1NBNE02EYECA-1 | ta | 11 | 117 | 138 | 167 |
| FW1NBNE02ESBZR-1 | ta | 5 | 55 | 64 | 130 |
| FW1NBNE02EY5M9-1 | ta | 5 | 126 | 135 | 310 |
| FW1NBNE02ELDM7-1 | ta | 8 | 46 | 61 | 135 |
| FW1NBNE02EDQJ2-1 | ta | 5 | 35 | 44 | 375 |
| contig04161-2 | taa | 5 | 245 | 259 | 296 |
| contig06850-2 | taa | 6 | 74 | 91 | 527 |
| contig09073-2 | taa | 6 | 692 | 709 | 716 |
| contig00063-1 | taa | 6 | 224 | 241 | 1166 |
| contig02179-1 | taa | 5 | 35 | 49 | 1053 |
| contig02180-1 | taa | 5 | 81 | 95 | 543 |
| contig07828-1 | taa | 5 | 67 | 81 | 1095 |
| FW1NBNE02C3JF3-1 | taa | 5 | 80 | 94 | 481 |
| FW1NBNE02EMBRB-1 | taa | 5 | 374 | 388 | 393 |
| FW1NBNE02DPOGA-3 | taac | 10 | 106 | 145 | 146 |
| FW1NBNE02CZBLT-2 | tac | 10 | 13 | 42 | 62 |
| contig12268-1 | tac | 5 | 202 | 216 | 372 |
| FW1NBNE02DER9F-1 | tac | 7 | 55 | 75 | 162 |
| FW1NBNE02C1058-1 | tac | 6 | 145 | 162 | 437 |
| contig03633-1 | tag | 6 | 151 | 168 | 522 |
| contig04528-1 | tag | 5 | 213 | 227 | 264 |
| contig10070-1 | tag | 5 | 332 | 346 | 380 |
| contig12341-1 | tag | 5 | 138 | 152 | 349 |
| FW1NBNE02DGHG3-1 | tag | 5 | 385 | 399 | 546 |
| FW1NBNE02EBWE5-1 | tag | 5 | 286 | 300 | 312 |
| FW1NBNE02EXP2M-1 | tag | 5 | 227 | 241 | 351 |
| contig06274-1 | tagat | 5 | 291 | 315 | 456 |
| contig00539-1 | tagc | 5 | 97 | 116 | 1901 |
| contig12437-1 | tagc | 5 | 22 | 41 | 424 |
| FW1NBNE02D4DGY-1 | tagtat | 5 | 19 | 48 | 505 |
| FW1NBNE02D58MH-4 | tat | 6 | 30 | 47 | 410 |
| contig01218-1 | tat | 5 | 15 | 29 | 567 |
| contig02104-1 | tat | 7 | 10 | 30 | 1342 |
| contig03055-1 | tat | 5 | 1700 | 1714 | 1918 |
| FW1NBNE02C11DR-1 | tat | 10 | 277 | 306 | 364 |
| FW1NBNE02DZ03J-1 | tat | 6 | 55 | 72 | 236 |
| FW1NBNE02EAG7B-1 | tat | 5 | 244 | 258 | 499 |
| FW1NBNE02D60PU-1 | tatc | 5 | 14 | 33 | 489 |
| contig01458-2 | tattac | 7 | 522 | 563 | 1029 |
| contig00323-2 | tc | 7 | 189 | 202 | 423 |
| contig02250-2 | tc | 14 | 474 | 501 | 1579 |
| contig05135-2 | tc | 6 | 536 | 547 | 551 |
| contig06835-2 | tc | 5 | 168 | 177 | 544 |
| contig06949-2 | tc | 5 | 43 | 52 | 1522 |
| contig08941-2 | tc | 6 | 407 | 418 | 475 |
| contig09164-2 | tc | 5 | 834 | 843 | 862 |
| contig10945-2 | tc | 5 | 498 | 507 | 556 |
| contig11825-2 | tc | 6 | 319 | 330 | 331 |
| contig13228-2 | tc | 5 | 189 | 198 | 301 |
| contig13259-2 | tc | 8 | 86 | 101 | 294 |
| FW1NBNE02C1P91-2 | tc | 5 | 26 | 35 | 57 |
| FW1NBNE02DR4FL-2 | tc | 8 | 49 | 64 | 390 |
| FW1NBNE02C59HZ-2 | tc | 6 | 499 | 510 | 522 |
| FW1NBNE02EBG4G-2 | tc | 8 | 96 | 111 | 532 |
| FW1NBNE02EKJY2-2 | tc | 6 | 403 | 414 | 426 |
| FW1NBNE02C29CZ-2 | tc | 7 | 248 | 261 | 369 |
| FW1NBNE02EGFRF-2 | tc | 5 | 194 | 203 | 349 |
| FW1NBNE02D5U69-2 | tc | 5 | 281 | 290 | 363 |
| FW1NBNE02DT559-2 | tc | 7 | 25 | 38 | 385 |
| FW1NBNE02DTTJJ-2 | tc | 6 | 104 | 115 | 277 |
| contig00119-1 | tc | 5 | 17 | 26 | 934 |
| contig00188-1 | tc | 5 | 698 | 707 | 1819 |
| contig00271-1 | tc | 8 | 1460 | 1475 | 1512 |
| contig00313-1 | tc | 6 | 16 | 27 | 1326 |
| contig00323-1 | tc | 5 | 177 | 186 | 423 |
| contig00328-1 | tc | 5 | 75 | 84 | 2586 |
| contig00512-1 | tc | 5 | 309 | 318 | 593 |
| contig00524-1 | tc | 5 | 465 | 474 | 648 |
| contig00682-1 | tc | 8 | 602 | 617 | 663 |
| contig00726-1 | tc | 6 | 245 | 256 | 602 |
| contig00746-1 | tc | 5 | 1289 | 1298 | 1325 |
| contig00801-1 | tc | 5 | 723 | 732 | 806 |
| contig01099-1 | tc | 5 | 1621 | 1630 | 1660 |
| contig01156-1 | tc | 5 | 34 | 43 | 2065 |
| contig01159-1 | tc | 9 | 2452 | 2469 | 2508 |
| contig01162-1 | tc | 5 | 646 | 655 | 689 |
| contig01203-1 | tc | 7 | 44 | 57 | 1670 |
| contig01235-1 | tc | 8 | 777 | 792 | 873 |
| contig01300-1 | tc | 5 | 164 | 173 | 1334 |
| contig01315-1 | tc | 5 | 591 | 600 | 642 |
| contig01571-1 | tc | 5 | 81 | 90 | 503 |
| contig01731-1 | tc | 8 | 140 | 155 | 977 |
| contig01769-1 | tc | 5 | 44 | 53 | 855 |
| contig01839-1 | tc | 6 | 77 | 88 | 491 |
| contig01847-1 | tc | 5 | 8 | 17 | 1003 |
| contig01871-1 | tc | 5 | 528 | 537 | 583 |
| contig01974-1 | tc | 6 | 236 | 247 | 345 |
| contig01976-1 | tc | 5 | 393 | 402 | 527 |
| contig01999-1 | tc | 5 | 84 | 93 | 1155 |
| contig02019-1 | tc | 5 | 38 | 47 | 966 |
| contig02060-1 | tc | 6 | 1026 | 1037 | 1111 |
| contig02512-1 | tc | 5 | 299 | 308 | 540 |
| contig02521-1 | tc | 14 | 1734 | 1761 | 1775 |
| contig02619-1 | tc | 5 | 1848 | 1857 | 2073 |
| contig02755-1 | tc | 5 | 4 | 13 | 455 |
| contig02856-1 | tc | 5 | 20 | 29 | 1624 |
| contig02948-1 | tc | 5 | 137 | 146 | 666 |
| contig03020-1 | tc | 5 | 142 | 151 | 1358 |
| contig03040-1 | tc | 7 | 13 | 26 | 750 |
| contig03065-1 | tc | 7 | 96 | 109 | 1086 |
| contig03073-1 | tc | 6 | 612 | 623 | 719 |
| contig03165-1 | tc | 5 | 102 | 111 | 666 |
| contig03185-1 | tc | 5 | 227 | 236 | 1089 |
| contig03401-1 | tc | 5 | 254 | 263 | 1226 |
| contig03462-1 | tc | 6 | 277 | 288 | 399 |
| contig03498-1 | tc | 5 | 138 | 147 | 351 |
| contig03537-1 | tc | 5 | 151 | 160 | 1444 |
| contig03623-1 | tc | 10 | 1112 | 1131 | 1153 |
| contig03764-1 | tc | 5 | 166 | 175 | 260 |
| contig03873-1 | tc | 5 | 455 | 464 | 571 |
| contig03945-1 | tc | 8 | 827 | 842 | 870 |
| contig03957-1 | tc | 5 | 324 | 333 | 568 |
| contig04054-1 | tc | 5 | 286 | 295 | 470 |
| contig04197-1 | tc | 7 | 547 | 560 | 917 |
| contig04217-1 | tc | 6 | 772 | 783 | 891 |
| contig04316-1 | tc | 5 | 474 | 483 | 568 |
| contig04331-1 | tc | 5 | 225 | 234 | 269 |
| contig04780-1 | tc | 6 | 349 | 360 | 361 |
| contig04793-1 | tc | 5 | 64 | 73 | 101 |
| contig04798-1 | tc | 5 | 610 | 619 | 683 |
| contig04839-1 | tc | 10 | 524 | 543 | 553 |
| contig04855-1 | tc | 5 | 766 | 775 | 787 |
| contig04856-1 | tc | 8 | 730 | 745 | 768 |
| contig04911-1 | tc | 5 | 19 | 28 | 2706 |
| contig04916-1 | tc | 8 | 417 | 432 | 1772 |
| contig05135-1 | tc | 7 | 510 | 523 | 551 |
| contig05400-1 | tc | 7 | 1899 | 1912 | 1964 |
| contig05402-1 | tc | 7 | 131 | 144 | 1419 |
| contig05610-1 | tc | 5 | 47 | 56 | 1675 |
| contig05681-1 | tc | 7 | 524 | 537 | 616 |
| contig05717-1 | tc | 5 | 312 | 321 | 391 |
| contig05842-1 | tc | 8 | 159 | 174 | 1015 |
| contig05964-1 | tc | 7 | 714 | 727 | 785 |
| contig06019-1 | tc | 5 | 27 | 36 | 514 |
| contig06119-1 | tc | 7 | 1619 | 1632 | 1662 |
| contig06278-1 | tc | 6 | 15 | 26 | 1364 |
| contig06289-1 | tc | 7 | 372 | 385 | 404 |
| contig06520-1 | tc | 5 | 13 | 22 | 591 |
| contig06592-1 | tc | 7 | 955 | 968 | 995 |
| contig06804-1 | tc | 6 | 57 | 68 | 514 |
| contig06823-1 | tc | 6 | 11 | 22 | 603 |
| contig06887-1 | tc | 5 | 171 | 180 | 1249 |
| contig06949-1 | tc | 5 | 31 | 40 | 1522 |
| contig06958-1 | tc | 6 | 338 | 349 | 353 |
| contig07037-1 | tc | 8 | 87 | 102 | 965 |
| contig07172-1 | tc | 5 | 20 | 29 | 232 |
| contig07209-1 | tc | 5 | 575 | 584 | 613 |
| contig07364-1 | tc | 5 | 125 | 134 | 540 |
| contig07553-1 | tc | 5 | 303 | 312 | 497 |
| contig07629-1 | tc | 6 | 12 | 23 | 613 |
| contig07686-1 | tc | 6 | 952 | 963 | 1001 |
| contig07733-1 | tc | 6 | 25 | 36 | 304 |
| contig07745-1 | tc | 6 | 123 | 134 | 501 |
| contig07753-1 | tc | 6 | 959 | 970 | 1001 |
| contig07834-1 | tc | 5 | 580 | 589 | 653 |
| contig07940-1 | tc | 5 | 582 | 591 | 672 |
| contig07976-1 | tc | 5 | 1140 | 1149 | 1273 |
| contig07990-1 | tc | 6 | 1723 | 1734 | 1734 |
| contig08088-1 | tc | 5 | 92 | 101 | 512 |
| contig08155-1 | tc | 5 | 241 | 250 | 433 |
| contig08409-1 | tc | 9 | 248 | 265 | 578 |
| contig08427-1 | tc | 7 | 440 | 453 | 479 |
| contig09100-1 | tc | 5 | 279 | 288 | 551 |
| contig09127-1 | tc | 6 | 422 | 433 | 437 |
| contig09143-1 | tc | 5 | 373 | 382 | 501 |
| contig09164-1 | tc | 5 | 820 | 829 | 862 |
| contig09270-1 | tc | 5 | 100 | 109 | 663 |
| contig09277-1 | tc | 6 | 350 | 361 | 372 |
| contig09284-1 | tc | 5 | 55 | 64 | 121 |
| contig09299-1 | tc | 5 | 339 | 348 | 493 |
| contig09365-1 | tc | 5 | 994 | 1003 | 1122 |
| contig09817-1 | tc | 5 | 587 | 596 | 630 |
| contig09896-1 | tc | 5 | 1084 | 1093 | 1103 |
| contig09988-1 | tc | 7 | 471 | 484 | 775 |
| contig10011-1 | tc | 5 | 513 | 522 | 591 |
| contig10331-1 | tc | 5 | 147 | 156 | 576 |
| contig10403-1 | tc | 5 | 18 | 27 | 488 |
| contig10561-1 | tc | 5 | 1149 | 1158 | 1187 |
| contig10571-1 | tc | 5 | 66 | 75 | 642 |
| contig10577-1 | tc | 8 | 602 | 617 | 659 |
| contig10606-1 | tc | 5 | 24 | 33 | 923 |
| contig10629-1 | tc | 5 | 27 | 36 | 247 |
| contig10714-1 | tc | 7 | 337 | 350 | 356 |
| contig10882-1 | tc | 5 | 71 | 80 | 358 |
| contig10932-1 | tc | 6 | 195 | 206 | 628 |
| contig10939-1 | tc | 5 | 4 | 13 | 540 |
| contig11090-1 | tc | 5 | 238 | 247 | 262 |
| contig11107-1 | tc | 7 | 20 | 33 | 374 |
| contig11200-1 | tc | 5 | 400 | 409 | 487 |
| contig11202-1 | tc | 5 | 472 | 481 | 716 |
| contig11382-1 | tc | 5 | 281 | 290 | 1303 |
| contig11391-1 | tc | 5 | 233 | 242 | 879 |
| contig11726-1 | tc | 5 | 171 | 180 | 1436 |
| contig11737-1 | tc | 6 | 72 | 83 | 621 |
| contig12011-1 | tc | 5 | 232 | 241 | 431 |
| contig12133-1 | tc | 5 | 11 | 20 | 466 |
| contig12196-1 | tc | 5 | 87 | 96 | 469 |
| contig12250-1 | tc | 9 | 14 | 31 | 244 |
| contig12275-1 | tc | 7 | 126 | 139 | 460 |
| contig12307-1 | tc | 5 | 295 | 304 | 465 |
| contig12310-1 | tc | 9 | 451 | 468 | 469 |
| contig12402-1 | tc | 5 | 390 | 399 | 458 |
| contig12638-1 | tc | 8 | 30 | 45 | 416 |
| contig12656-1 | tc | 5 | 200 | 209 | 330 |
| contig12688-1 | tc | 5 | 393 | 402 | 448 |
| contig12775-1 | tc | 6 | 263 | 274 | 404 |
| contig12787-1 | tc | 5 | 23 | 32 | 304 |
| contig12799-1 | tc | 5 | 128 | 137 | 338 |
| contig12889-1 | tc | 6 | 57 | 68 | 427 |
| contig12947-1 | tc | 5 | 46 | 55 | 415 |
| contig12987-1 | tc | 7 | 89 | 102 | 399 |
| contig13186-1 | tc | 6 | 69 | 80 | 321 |
| contig13188-1 | tc | 8 | 207 | 222 | 265 |
| contig13236-1 | tc | 5 | 207 | 216 | 245 |
| contig13281-1 | tc | 16 | 719 | 750 | 798 |
| contig13351-1 | tc | 6 | 543 | 554 | 636 |
| contig13418-1 | tc | 5 | 68 | 77 | 140 |
| contig13424-1 | tc | 5 | 74 | 83 | 574 |
| contig13804-1 | tc | 8 | 352 | 367 | 459 |
| contig13927-1 | tc | 12 | 322 | 345 | 386 |
| FW1NBNE02D0PZS-1 | tc | 5 | 432 | 441 | 444 |
| FW1NBNE02DOLUW-1 | tc | 5 | 38 | 47 | 138 |
| FW1NBNE02CZWZY-1 | tc | 5 | 47 | 56 | 59 |
| FW1NBNE02D6II6-1 | tc | 5 | 126 | 135 | 514 |
| FW1NBNE02EBYAM-1 | tc | 6 | 54 | 65 | 432 |
| FW1NBNE02DVR7E-1 | tc | 7 | 470 | 483 | 485 |
| FW1NBNE02C0LNR-1 | tc | 5 | 11 | 20 | 205 |
| FW1NBNE02EO1MN-1 | tc | 5 | 26 | 35 | 96 |
| FW1NBNE02ENPPF-1 | tc | 6 | 408 | 419 | 522 |
| FW1NBNE02EPHB9-1 | tc | 6 | 181 | 192 | 572 |
| FW1NBNE02DNCSY-1 | tc | 5 | 232 | 241 | 471 |
| FW1NBNE02DG607-1 | tc | 7 | 85 | 98 | 148 |
| FW1NBNE02D388T-1 | tc | 5 | 83 | 92 | 97 |
| FW1NBNE02D4D34-1 | tc | 6 | 50 | 61 | 494 |
| FW1NBNE02C1E8U-1 | tc | 5 | 109 | 118 | 301 |
| FW1NBNE02D0JBU-1 | tc | 5 | 106 | 115 | 466 |
| FW1NBNE02EUW22-1 | tc | 6 | 500 | 511 | 511 |
| FW1NBNE02C3QQ4-1 | tc | 14 | 138 | 165 | 186 |
| FW1NBNE02C0019-1 | tc | 6 | 59 | 70 | 71 |
| FW1NBNE02EXCY2-1 | tc | 6 | 64 | 75 | 427 |
| FW1NBNE02DLP09-1 | tc | 5 | 38 | 47 | 442 |
| FW1NBNE02DOQVJ-1 | tc | 8 | 130 | 145 | 274 |
| FW1NBNE02DNTC7-1 | tc | 7 | 23 | 36 | 396 |
| FW1NBNE02DJQLM-1 | tc | 6 | 27 | 38 | 510 |
| FW1NBNE02D9KL5-1 | tc | 5 | 420 | 429 | 491 |
| FW1NBNE02DLEBD-1 | tc | 5 | 30 | 39 | 209 |
| FW1NBNE02EF58S-1 | tc | 18 | 32 | 67 | 68 |
| FW1NBNE02DZ7W7-1 | tc | 5 | 29 | 38 | 326 |
| FW1NBNE02EBVEC-1 | tc | 5 | 293 | 302 | 551 |
| FW1NBNE02D0438-1 | tc | 5 | 136 | 145 | 306 |
| FW1NBNE02ESRUR-1 | tc | 5 | 47 | 56 | 76 |
| FW1NBNE02EB3KK-1 | tc | 5 | 18 | 27 | 126 |
| FW1NBNE02C2ORU-1 | tc | 6 | 66 | 77 | 554 |
| FW1NBNE02DUTHX-1 | tc | 12 | 33 | 56 | 474 |
| FW1NBNE02DTE56-1 | tc | 9 | 134 | 151 | 170 |
| FW1NBNE02C1P91-1 | tc | 5 | 14 | 23 | 57 |
| FW1NBNE02EDA38-1 | tc | 5 | 28 | 37 | 502 |
| FW1NBNE02ERXLX-1 | tc | 5 | 88 | 97 | 367 |
| FW1NBNE02DR4FL-1 | tc | 5 | 19 | 28 | 390 |
| FW1NBNE02DLKIX-1 | tc | 5 | 55 | 64 | 436 |
| FW1NBNE02D3FMI-1 | tc | 5 | 481 | 490 | 519 |
| FW1NBNE02C1ZLL-1 | tc | 10 | 392 | 411 | 493 |
| FW1NBNE02EHIYA-1 | tc | 8 | 465 | 480 | 506 |
| FW1NBNE02EUYD0-1 | tc | 8 | 27 | 42 | 459 |
| FW1NBNE02EQNWY-1 | tc | 5 | 35 | 44 | 74 |
| FW1NBNE02C59HZ-1 | tc | 5 | 487 | 496 | 522 |
| FW1NBNE02EOQJ7-1 | tc | 8 | 463 | 478 | 479 |
| FW1NBNE02D9HIW-1 | tc | 5 | 338 | 347 | 515 |
| FW1NBNE02C8JA7-1 | tc | 5 | 244 | 253 | 496 |
| FW1NBNE02D1Z0B-1 | tc | 7 | 205 | 218 | 534 |
| FW1NBNE02DRPEU-1 | tc | 9 | 99 | 116 | 185 |
| FW1NBNE02EBG4G-1 | tc | 5 | 11 | 20 | 532 |
| FW1NBNE02DLB9J-1 | tc | 5 | 70 | 79 | 199 |
| FW1NBNE02EFSY7-1 | tc | 8 | 85 | 100 | 231 |
| FW1NBNE02EUWPY-1 | tc | 5 | 149 | 158 | 541 |
| FW1NBNE02EPPEQ-1 | tc | 7 | 135 | 148 | 318 |
| FW1NBNE02DNPQW-1 | tc | 10 | 23 | 42 | 72 |
| FW1NBNE02DYO2D-1 | tc | 5 | 124 | 133 | 468 |
| FW1NBNE02ETG7J-1 | tc | 5 | 47 | 56 | 70 |
| FW1NBNE02C437P-1 | tc | 5 | 44 | 53 | 57 |
| FW1NBNE02EKJY2-1 | tc | 5 | 391 | 400 | 426 |
| FW1NBNE02C4WNN-1 | tc | 5 | 29 | 38 | 408 |
| FW1NBNE02DEGI9-1 | tc | 5 | 180 | 189 | 433 |
| FW1NBNE02DBAH9-1 | tc | 9 | 48 | 65 | 80 |
| FW1NBNE02DB1J6-1 | tc | 5 | 91 | 100 | 425 |
| FW1NBNE02C5TD5-1 | tc | 8 | 88 | 103 | 267 |
| FW1NBNE02ES6DY-1 | tc | 5 | 344 | 353 | 489 |
| FW1NBNE02DD42P-1 | tc | 6 | 350 | 361 | 450 |
| FW1NBNE02DUOWA-1 | tc | 5 | 20 | 29 | 524 |
| FW1NBNE02EWJLQ-1 | tc | 9 | 3 | 20 | 475 |
| FW1NBNE02DVVCM-1 | tc | 7 | 143 | 156 | 409 |
| FW1NBNE02DRH3L-1 | tc | 5 | 123 | 132 | 501 |
| FW1NBNE02C8GOL-1 | tc | 5 | 344 | 353 | 534 |
| FW1NBNE02ECH09-1 | tc | 7 | 61 | 74 | 169 |
| FW1NBNE02DPRFF-1 | tc | 5 | 80 | 89 | 451 |
| FW1NBNE02DII1X-1 | tc | 5 | 43 | 52 | 514 |
| FW1NBNE02C6QTD-1 | tc | 5 | 113 | 122 | 459 |
| FW1NBNE02D4TXX-1 | tc | 6 | 494 | 505 | 520 |
| FW1NBNE02DS73D-1 | tc | 9 | 285 | 302 | 364 |
| FW1NBNE02ERV6O-1 | tc | 6 | 154 | 165 | 517 |
| FW1NBNE02DU2DJ-1 | tc | 9 | 24 | 41 | 73 |
| FW1NBNE02DV592-1 | tc | 7 | 71 | 84 | 90 |
| FW1NBNE02EA17R-1 | tc | 7 | 47 | 60 | 60 |
| FW1NBNE02ENECS-1 | tc | 6 | 18 | 29 | 468 |
| FW1NBNE02EURUW-1 | tc | 5 | 235 | 244 | 475 |
| FW1NBNE02DJ5C8-1 | tc | 5 | 52 | 61 | 468 |
| FW1NBNE02EC6Y2-1 | tc | 5 | 36 | 45 | 291 |
| FW1NBNE02EUYMF-1 | tc | 8 | 459 | 474 | 482 |
| FW1NBNE02DX6V7-1 | tc | 6 | 470 | 481 | 518 |
| FW1NBNE02EQG3F-1 | tc | 7 | 8 | 21 | 190 |
| FW1NBNE02DEASR-1 | tc | 5 | 59 | 68 | 68 |
| FW1NBNE02EGVQ3-1 | tc | 5 | 132 | 141 | 480 |
| FW1NBNE02EXQWL-1 | tc | 7 | 233 | 246 | 339 |
| FW1NBNE02EHUYI-1 | tc | 5 | 47 | 56 | 505 |
| FW1NBNE02DW9W9-1 | tc | 5 | 363 | 372 | 411 |
| FW1NBNE02EEAAW-1 | tc | 6 | 191 | 202 | 221 |
| FW1NBNE02DMLXY-1 | tc | 5 | 41 | 50 | 452 |
| FW1NBNE02EDEOM-1 | tc | 7 | 26 | 39 | 330 |
| FW1NBNE02EXLSO-1 | tc | 5 | 37 | 46 | 223 |
| FW1NBNE02EISTI-1 | tc | 5 | 31 | 40 | 145 |
| FW1NBNE02DKK95-1 | tc | 19 | 39 | 76 | 76 |
| FW1NBNE02C0HKC-1 | tc | 6 | 21 | 32 | 455 |
| FW1NBNE02C10CM-1 | tc | 9 | 35 | 52 | 66 |
| FW1NBNE02EHX1Q-1 | tc | 5 | 56 | 65 | 457 |
| FW1NBNE02EPVKP-1 | tc | 5 | 234 | 243 | 312 |
| FW1NBNE02CZOC3-1 | tc | 5 | 438 | 447 | 461 |
| FW1NBNE02EGD1M-1 | tc | 5 | 78 | 87 | 327 |
| FW1NBNE02DI7JY-1 | tc | 5 | 15 | 24 | 68 |
| FW1NBNE02EJ2HK-1 | tc | 5 | 176 | 185 | 412 |
| FW1NBNE02DII6X-1 | tc | 6 | 288 | 299 | 509 |
| FW1NBNE02ELBE3-1 | tc | 5 | 128 | 137 | 274 |
| FW1NBNE02ENW2E-1 | tc | 5 | 108 | 117 | 432 |
| FW1NBNE02C9021-1 | tc | 5 | 76 | 85 | 509 |
| FW1NBNE02EM75Y-1 | tc | 5 | 408 | 417 | 455 |
| FW1NBNE02DNBZ1-1 | tc | 6 | 313 | 324 | 352 |
| FW1NBNE02ENLOP-1 | tc | 7 | 127 | 140 | 192 |
| FW1NBNE02DE7BR-1 | tc | 5 | 83 | 92 | 419 |
| FW1NBNE02DPXU6-1 | tc | 6 | 48 | 59 | 213 |
| FW1NBNE02EWYV9-1 | tc | 6 | 19 | 30 | 418 |
| FW1NBNE02C1SR8-1 | tc | 8 | 351 | 366 | 401 |
| FW1NBNE02EUPMO-1 | tc | 5 | 84 | 93 | 379 |
| FW1NBNE02DXCGY-1 | tc | 5 | 40 | 49 | 471 |
| FW1NBNE02D4T6W-1 | tc | 6 | 257 | 268 | 463 |
| FW1NBNE02C3VJZ-1 | tc | 5 | 36 | 45 | 297 |
| FW1NBNE02EU7UH-1 | tc | 17 | 47 | 80 | 81 |
| FW1NBNE02DG9LI-1 | tc | 5 | 39 | 48 | 354 |
| FW1NBNE02CZ1EY-1 | tc | 5 | 269 | 278 | 388 |
| FW1NBNE02D6HUJ-1 | tc | 5 | 95 | 104 | 537 |
| FW1NBNE02C821N-1 | tc | 5 | 331 | 340 | 392 |
| FW1NBNE02EIRI0-1 | tc | 8 | 49 | 64 | 159 |
| FW1NBNE02EK9AW-1 | tc | 6 | 42 | 53 | 432 |
| FW1NBNE02ERY8R-1 | tc | 5 | 92 | 101 | 429 |
| FW1NBNE02DOJJV-1 | tc | 5 | 393 | 402 | 474 |
| FW1NBNE02D7WLS-1 | tc | 6 | 28 | 39 | 493 |
| FW1NBNE02D5TI0-1 | tc | 11 | 196 | 217 | 380 |
| FW1NBNE02DVQVM-1 | tc | 5 | 134 | 143 | 368 |
| FW1NBNE02D2TPQ-1 | tc | 5 | 6 | 15 | 400 |
| FW1NBNE02C4IX1-1 | tc | 5 | 30 | 39 | 445 |
| FW1NBNE02DL694-1 | tc | 6 | 69 | 80 | 351 |
| FW1NBNE02ESRM0-1 | tc | 6 | 88 | 99 | 99 |
| FW1NBNE02C8T44-1 | tc | 5 | 488 | 497 | 499 |
| FW1NBNE02DAP6H-1 | tc | 5 | 70 | 79 | 302 |
| FW1NBNE02EHX0D-1 | tc | 5 | 71 | 80 | 363 |
| FW1NBNE02EAIRW-1 | tc | 5 | 67 | 76 | 207 |
| FW1NBNE02DGDZI-1 | tc | 9 | 37 | 54 | 55 |
| FW1NBNE02DZLQ8-1 | tc | 9 | 55 | 72 | 262 |
| FW1NBNE02DTTJJ-1 | tc | 10 | 13 | 32 | 277 |
| FW1NBNE02DH62P-1 | tc | 5 | 51 | 60 | 364 |
| FW1NBNE02DX3SQ-1 | tc | 5 | 35 | 44 | 279 |
| FW1NBNE02EA807-1 | tc | 7 | 122 | 135 | 181 |
| FW1NBNE02ESN5E-1 | tc | 9 | 141 | 158 | 257 |
| FW1NBNE02D2L4G-1 | tc | 7 | 52 | 65 | 112 |
| contig03161-2 | tca | 8 | 597 | 620 | 1155 |
| contig03534-2 | tca | 6 | 312 | 329 | 1033 |
| contig03760-2 | tca | 5 | 258 | 272 | 1205 |
| contig06796-2 | tca | 5 | 857 | 871 | 1019 |
| contig12384-2 | tca | 5 | 141 | 155 | 855 |
| contig02022-1 | tca | 6 | 77 | 94 | 2011 |
| contig03302-1 | tca | 6 | 351 | 368 | 671 |
| contig04578-1 | tca | 10 | 313 | 342 | 495 |
| contig04608-1 | tca | 5 | 767 | 781 | 1253 |
| contig04784-1 | tca | 10 | 981 | 1010 | 1392 |
| contig05451-1 | tca | 5 | 284 | 298 | 520 |
| contig06092-1 | tca | 8 | 295 | 318 | 546 |
| contig07422-1 | tca | 6 | 294 | 311 | 1312 |
| contig10848-1 | tca | 6 | 277 | 294 | 483 |
| FW1NBNE02D5YB7-1 | tca | 6 | 347 | 364 | 529 |
| FW1NBNE02DFG48-1 | tca | 8 | 322 | 345 | 490 |
| FW1NBNE02DVGM7-1 | tca | 5 | 267 | 281 | 497 |
| FW1NBNE02DX1J1-1 | tca | 5 | 20 | 34 | 86 |
| FW1NBNE02DPL23-1 | tca | 5 | 368 | 382 | 550 |
| FW1NBNE02DJQHZ-1 | tca | 6 | 25 | 42 | 129 |
| FW1NBNE02DNW1Q-1 | tcaggc | 12 | 96 | 167 | 172 |
| FW1NBNE02DWK95-2 | tcc | 5 | 39 | 53 | 104 |
| FW1NBNE02DYL29-2 | tcc | 5 | 9 | 23 | 474 |
| FW1NBNE02ET1N8-2 | tcc | 6 | 14 | 31 | 274 |
| contig04506-4 | tcc | 5 | 119 | 133 | 2261 |
| contig00538-1 | tcc | 7 | 210 | 230 | 1190 |
| contig02138-1 | tcc | 5 | 85 | 99 | 886 |
| contig09098-1 | tcc | 5 | 697 | 711 | 792 |
| contig09814-1 | tcc | 5 | 250 | 264 | 484 |
| contig12384-1 | tcc | 6 | 14 | 31 | 855 |
| FW1NBNE02C8QBA-1 | tcc | 7 | 27 | 47 | 381 |
| FW1NBNE02CZJTE-1 | tcc | 7 | 182 | 202 | 441 |
| FW1NBNE02DFWKF-1 | tcc | 5 | 40 | 54 | 84 |
| FW1NBNE02C6OEP-1 | tcc | 5 | 37 | 51 | 483 |
| FW1NBNE02C6COY-1 | tccagc | 5 | 207 | 236 | 287 |
| contig00159-1 | tccc | 7 | 1653 | 1680 | 1704 |
| FW1NBNE02DIPUE-1 | tccg | 5 | 179 | 198 | 237 |
| contig03421-1 | tccga | 5 | 131 | 155 | 156 |
| contig08265-1 | tcctg | 5 | 143 | 167 | 246 |
| FW1NBNE02DUTKH-1 | tcg | 8 | 443 | 466 | 475 |
| FW1NBNE02D5RMU-1 | tcg | 6 | 470 | 487 | 508 |
| FW1NBNE02ELNZT-1 | tcgccg | 5 | 188 | 217 | 402 |
| FW1NBNE02C4VKG-3 | tcgg | 7 | 92 | 119 | 430 |
| contig08453-1 | tcgt | 5 | 2 | 21 | 498 |
| contig00853-2 | tct | 6 | 318 | 335 | 1530 |
| contig01051-2 | tct | 5 | 2275 | 2289 | 2590 |
| contig03841-2 | tct | 5 | 434 | 448 | 576 |
| contig13281-2 | tct | 6 | 695 | 712 | 798 |
| contig13927-2 | tct | 7 | 304 | 324 | 386 |
| FW1NBNE02DNPOT-2 | tct | 5 | 445 | 459 | 482 |
| FW1NBNE02ES9WF-2 | tct | 6 | 108 | 125 | 477 |
| FW1NBNE02EBZYY-2 | tct | 7 | 391 | 411 | 450 |
| contig03534-3 | tct | 6 | 345 | 362 | 1033 |
| contig00706-1 | tct | 6 | 115 | 132 | 1659 |
| contig02388-1 | tct | 5 | 13 | 27 | 273 |
| contig02916-1 | tct | 5 | 317 | 331 | 533 |
| contig02987-1 | tct | 8 | 924 | 947 | 986 |
| contig04565-1 | tct | 7 | 55 | 75 | 597 |
| contig04735-1 | tct | 7 | 203 | 223 | 1082 |
| contig08307-1 | tct | 5 | 39 | 53 | 289 |
| contig10262-1 | tct | 6 | 283 | 300 | 490 |
| contig10810-1 | tct | 5 | 360 | 374 | 443 |
| contig11247-1 | tct | 5 | 77 | 91 | 271 |
| contig12374-1 | tct | 5 | 413 | 427 | 450 |
| contig12527-1 | tct | 7 | 129 | 149 | 388 |
| contig12657-1 | tct | 9 | 101 | 127 | 371 |
| FW1NBNE02DEVII-1 | tct | 5 | 137 | 151 | 475 |
| FW1NBNE02D6JD3-1 | tct | 7 | 25 | 45 | 536 |
| FW1NBNE02DPWS4-1 | tct | 6 | 435 | 452 | 498 |
| FW1NBNE02DNHLV-1 | tct | 7 | 43 | 63 | 542 |
| FW1NBNE02ERXA0-1 | tct | 6 | 280 | 297 | 449 |
| FW1NBNE02DQA9V-1 | tct | 6 | 488 | 505 | 515 |
| FW1NBNE02EF7EF-1 | tct | 6 | 470 | 487 | 512 |
| FW1NBNE02ENNVR-1 | tct | 5 | 456 | 470 | 472 |
| FW1NBNE02D00IG-1 | tct | 5 | 283 | 297 | 470 |
| FW1NBNE02DMP9Q-1 | tct | 6 | 164 | 181 | 469 |
| FW1NBNE02DNV58-1 | tct | 6 | 132 | 149 | 436 |
| FW1NBNE02CZ2CR-2 | tcta | 7 | 72 | 99 | 343 |
| FW1NBNE02EAIGZ-1 | tctt | 6 | 75 | 98 | 491 |
| FW1NBNE02EF51O-1 | tctt | 9 | 68 | 103 | 208 |
| contig10102-1 | tcttca | 6 | 382 | 417 | 505 |
| contig05558-2 | tg | 5 | 1144 | 1153 | 1157 |
| contig06617-2 | tg | 5 | 812 | 821 | 1068 |
| contig09343-2 | tg | 9 | 482 | 499 | 501 |
| contig12772-2 | tg | 5 | 128 | 137 | 424 |
| contig13607-2 | tg | 6 | 301 | 312 | 345 |
| FW1NBNE02ECEJ8-2 | tg | 34 | 20 | 87 | 88 |
| FW1NBNE02C261O-2 | tg | 23 | 22 | 67 | 68 |
| FW1NBNE02D7EYC-2 | tg | 17 | 510 | 543 | 543 |
| FW1NBNE02EB731-2 | tg | 16 | 176 | 207 | 220 |
| FW1NBNE02D4905-2 | tg | 10 | 72 | 91 | 91 |
| FW1NBNE02DRJHE-2 | tg | 23 | 20 | 65 | 66 |
| FW1NBNE02EG3NV-2 | tg | 21 | 29 | 70 | 75 |
| FW1NBNE02D7IY3-2 | tg | 5 | 48 | 57 | 404 |
| contig00118-3 | tg | 5 | 501 | 510 | 621 |
| FW1NBNE02C8POD-3 | tg | 9 | 197 | 214 | 214 |
| FW1NBNE02C3RBT-3 | tg | 13 | 183 | 208 | 209 |
| contig00956-1 | tg | 5 | 98 | 107 | 595 |
| contig01153-1 | tg | 5 | 395 | 404 | 430 |
| contig01652-1 | tg | 5 | 551 | 560 | 577 |
| contig03577-1 | tg | 17 | 549 | 582 | 801 |
| contig03817-1 | tg | 6 | 257 | 268 | 686 |
| contig04401-1 | tg | 5 | 777 | 786 | 998 |
| contig05837-1 | tg | 5 | 238 | 247 | 377 |
| contig06458-1 | tg | 6 | 515 | 526 | 567 |
| contig08228-1 | tg | 5 | 1066 | 1075 | 1192 |
| contig09831-1 | tg | 6 | 554 | 565 | 676 |
| contig10468-1 | tg | 5 | 910 | 919 | 986 |
| contig10578-1 | tg | 5 | 397 | 406 | 462 |
| contig12570-1 | tg | 9 | 458 | 475 | 622 |
| contig13242-1 | tg | 5 | 252 | 261 | 279 |
| FW1NBNE02DZX7G-1 | tg | 24 | 5 | 52 | 53 |
| FW1NBNE02DEQL5-1 | tg | 13 | 152 | 177 | 221 |
| FW1NBNE02DUD8I-1 | tg | 9 | 34 | 51 | 85 |
| FW1NBNE02C47Y2-1 | tg | 9 | 96 | 113 | 505 |
| FW1NBNE02DFH8Z-1 | tg | 11 | 243 | 264 | 329 |
| FW1NBNE02DWBMW-1 | tg | 5 | 33 | 42 | 398 |
| FW1NBNE02D8LYW-1 | tg | 11 | 442 | 463 | 492 |
| FW1NBNE02DBJ7Z-1 | tg | 5 | 412 | 421 | 471 |
| FW1NBNE02C8CU1-1 | tg | 5 | 45 | 54 | 93 |
| FW1NBNE02DH40R-1 | tg | 5 | 29 | 38 | 488 |
| FW1NBNE02DSXZF-1 | tg | 7 | 494 | 507 | 510 |
| FW1NBNE02C6YAM-1 | tg | 8 | 284 | 299 | 391 |
| FW1NBNE02D13FM-1 | tg | 11 | 476 | 497 | 522 |
| FW1NBNE02EGFRF-1 | tg | 6 | 127 | 138 | 349 |
| FW1NBNE02C107Z-1 | tg | 9 | 32 | 49 | 142 |
| FW1NBNE02DUH9T-1 | tg | 5 | 313 | 322 | 363 |
| FW1NBNE02EM8RU-1 | tg | 5 | 356 | 365 | 465 |
| FW1NBNE02D2BRC-1 | tg | 14 | 123 | 150 | 166 |
| FW1NBNE02EY36G-1 | tg | 7 | 44 | 57 | 62 |
| FW1NBNE02D6R87-1 | tg | 5 | 385 | 394 | 424 |
| FW1NBNE02C37Y1-1 | tg | 5 | 403 | 412 | 500 |
| contig01852-2 | tga | 6 | 1618 | 1635 | 1752 |
| contig10468-2 | tga | 5 | 714 | 728 | 986 |
| contig12765-2 | tga | 5 | 204 | 218 | 508 |
| FW1NBNE02DYL29-3 | tga | 5 | 417 | 431 | 474 |
| FW1NBNE02DYDXC-3 | tga | 9 | 178 | 204 | 319 |
| contig01688-1 | tga | 5 | 300 | 314 | 801 |
| contig01805-1 | tga | 5 | 873 | 887 | 1151 |
| contig04066-1 | tga | 5 | 545 | 559 | 891 |
| contig04077-1 | tga | 6 | 322 | 339 | 568 |
| contig05273-1 | tga | 5 | 137 | 151 | 1894 |
| contig06779-1 | tga | 6 | 134 | 151 | 334 |
| FW1NBNE02DM71U-1 | tga | 8 | 219 | 242 | 458 |
| FW1NBNE02EBVO5-1 | tga | 10 | 31 | 60 | 173 |
| FW1NBNE02EKKPA-1 | tga | 5 | 1 | 15 | 179 |
| FW1NBNE02EMWU9-1 | tga | 6 | 166 | 183 | 507 |
| FW1NBNE02DDJW9-1 | tga | 5 | 402 | 416 | 420 |
| FW1NBNE02C8847-1 | tga | 6 | 451 | 468 | 470 |
| FW1NBNE02DC1J9-1 | tga | 5 | 22 | 36 | 137 |
| FW1NBNE02EG06N-1 | tga | 5 | 174 | 188 | 327 |
| FW1NBNE02D173Q-1 | tga | 7 | 460 | 480 | 484 |
| FW1NBNE02D9L0C-1 | tga | 5 | 192 | 206 | 339 |
| FW1NBNE02DXB7I-1 | tga | 6 | 87 | 104 | 297 |
| FW1NBNE02EG8DO-1 | tgaag | 5 | 377 | 401 | 409 |
| contig05172-1 | tgag | 8 | 2381 | 2412 | 2477 |
| contig08726-1 | tgagac | 5 | 44 | 73 | 423 |
| contig02189-2 | tgc | 6 | 1364 | 1381 | 1960 |
| contig07224-2 | tgc | 5 | 224 | 238 | 589 |
| contig10544-2 | tgc | 7 | 327 | 347 | 446 |
| contig00786-1 | tgc | 6 | 597 | 614 | 627 |
| contig04001-1 | tgc | 5 | 462 | 476 | 617 |
| contig10941-1 | tgc | 6 | 201 | 218 | 748 |
| contig11336-1 | tgc | 5 | 406 | 420 | 455 |
| FW1NBNE02EP46D-1 | tgc | 5 | 166 | 180 | 537 |
| FW1NBNE02DUOV0-1 | tgc | 6 | 112 | 129 | 358 |
| FW1NBNE02EOXMP-1 | tgc | 6 | 250 | 267 | 546 |
| FW1NBNE02EJDA3-1 | tgc | 5 | 6 | 20 | 162 |
| FW1NBNE02ER4R7-1 | tgc | 5 | 396 | 410 | 414 |
| contig04698-2 | tgcc | 6 | 316 | 339 | 496 |
| contig07303-1 | tgcc | 5 | 250 | 269 | 1235 |
| contig10702-1 | tgcc | 5 | 547 | 566 | 638 |
| contig12394-1 | tgccc | 5 | 15 | 39 | 633 |
| FW1NBNE02DG8RH-1 | tgctgg | 5 | 275 | 304 | 477 |
| contig06173-2 | tgg | 7 | 270 | 290 | 364 |
| contig00663-1 | tgg | 5 | 62 | 76 | 651 |
| contig06716-1 | tgg | 5 | 350 | 364 | 481 |
| contig08121-1 | tgg | 5 | 109 | 123 | 542 |
| contig10156-1 | tgg | 6 | 164 | 181 | 318 |
| FW1NBNE02C4YRF-1 | tgg | 5 | 368 | 382 | 424 |
| FW1NBNE02DV8YM-1 | tgg | 6 | 425 | 442 | 491 |
| FW1NBNE02DRMHS-1 | tgg | 5 | 12 | 26 | 88 |
| FW1NBNE02EL0IG-1 | tgg | 6 | 176 | 193 | 501 |
| FW1NBNE02EMQ78-1 | tgg | 6 | 52 | 69 | 403 |
| FW1NBNE02EFKRY-1 | tgg | 8 | 112 | 135 | 186 |
| FW1NBNE02EODML-1 | tgg | 5 | 306 | 320 | 546 |
| FW1NBNE02C2NQ7-1 | tgg | 5 | 363 | 377 | 413 |
| FW1NBNE02C842J-1 | tgg | 7 | 343 | 363 | 529 |
| FW1NBNE02EPVPA-1 | tgg | 7 | 22 | 42 | 438 |
| contig09940-1 | tgga | 5 | 811 | 830 | 869 |
| FW1NBNE02D9T7A-1 | tgga | 5 | 46 | 65 | 325 |
| contig10333-1 | tggaaa | 5 | 138 | 167 | 528 |
| contig07712-1 | tggaag | 6 | 368 | 403 | 514 |
| FW1NBNE02EPO7I-1 | tggat | 5 | 136 | 160 | 472 |
| contig05667-2 | tgt | 8 | 1291 | 1314 | 1707 |
| contig00971-3 | tgt | 5 | 1216 | 1230 | 1329 |
| contig10544-3 | tgt | 5 | 366 | 380 | 446 |
| contig10544-1 | tgt | 6 | 309 | 326 | 446 |
| FW1NBNE02DO06I-1 | tgt | 5 | 332 | 346 | 553 |
| FW1NBNE02C4HSX-1 | tgt | 5 | 375 | 389 | 454 |
| FW1NBNE02DKWMI-1 | tgt | 6 | 211 | 228 | 568 |
| contig02853-1 | tgttgg | 5 | 103 | 132 | 260 |
| contig00683-2 | tta | 11 | 872 | 904 | 1046 |
| FW1NBNE02ENBY4-2 | tta | 5 | 18 | 32 | 61 |
| FW1NBNE02EMUSQ-2 | tta | 5 | 53 | 67 | 181 |
| FW1NBNE02D84SX-2 | tta | 5 | 95 | 109 | 221 |
| FW1NBNE02DY3TL-2 | tta | 5 | 26 | 40 | 56 |
| FW1NBNE02EU7U5-2 | tta | 7 | 107 | 127 | 139 |
| contig01558-1 | tta | 6 | 402 | 419 | 512 |
| contig01720-1 | tta | 5 | 150 | 164 | 1514 |
| contig06827-1 | tta | 5 | 889 | 903 | 1138 |
| contig13044-1 | tta | 5 | 347 | 361 | 361 |
| contig13967-1 | tta | 5 | 92 | 106 | 156 |
| FW1NBNE02C0PY9-1 | tta | 6 | 466 | 483 | 505 |
| FW1NBNE02C5D97-1 | tta | 5 | 443 | 457 | 480 |
| FW1NBNE02D0547-1 | tta | 10 | 534 | 563 | 566 |
| contig01577-1 | ttag | 5 | 651 | 670 | 943 |
| FW1NBNE02EQIIK-1 | ttagc | 5 | 193 | 217 | 230 |
| FW1NBNE02DVLU7-3 | ttat | 6 | 146 | 169 | 318 |
| FW1NBNE02EJA6O-1 | ttat | 6 | 55 | 78 | 345 |
| contig00659-2 | ttc | 6 | 1427 | 1444 | 1696 |
| contig00850-2 | ttc | 7 | 79 | 99 | 938 |
| FW1NBNE02EPPEQ-2 | ttc | 5 | 122 | 136 | 318 |
| contig00567-1 | ttc | 5 | 341 | 355 | 505 |
| contig00930-1 | ttc | 9 | 797 | 823 | 1071 |
| contig01051-1 | ttc | 5 | 2117 | 2131 | 2590 |
| contig04250-1 | ttc | 6 | 611 | 628 | 686 |
| contig04361-1 | ttc | 5 | 684 | 698 | 781 |
| contig04457-1 | ttc | 5 | 1559 | 1573 | 2180 |
| contig05247-1 | ttc | 5 | 630 | 644 | 712 |
| contig05418-1 | ttc | 5 | 1193 | 1207 | 1335 |
| contig06565-1 | ttc | 6 | 47 | 64 | 668 |
| contig07405-1 | ttc | 6 | 105 | 122 | 1121 |
| contig08520-1 | ttc | 5 | 767 | 781 | 1195 |
| contig08572-1 | ttc | 5 | 4 | 18 | 479 |
| contig10267-1 | ttc | 5 | 810 | 824 | 832 |
| contig11783-1 | ttc | 5 | 176 | 190 | 491 |
| contig12986-1 | ttc | 6 | 54 | 71 | 384 |
| contig13448-1 | ttc | 5 | 183 | 197 | 209 |
| FW1NBNE02EQGO3-1 | ttc | 9 | 162 | 188 | 502 |
| FW1NBNE02EMVQE-1 | ttc | 5 | 208 | 222 | 439 |
| FW1NBNE02DPZ4F-1 | ttc | 6 | 288 | 305 | 511 |
| FW1NBNE02D6OE6-1 | ttc | 5 | 262 | 276 | 468 |
| FW1NBNE02ENCXB-1 | ttc | 5 | 218 | 232 | 483 |
| FW1NBNE02EIKOH-1 | ttc | 5 | 87 | 101 | 220 |
| FW1NBNE02DTZKO-1 | ttc | 7 | 1 | 21 | 538 |
| FW1NBNE02DZ2HP-1 | ttc | 6 | 415 | 432 | 492 |
| FW1NBNE02CZY0W-1 | ttc | 5 | 31 | 45 | 197 |
| FW1NBNE02ETJRT-1 | ttc | 5 | 161 | 175 | 442 |
| FW1NBNE02EJ67M-1 | ttc | 5 | 104 | 118 | 369 |
| FW1NBNE02EVY46-1 | ttc | 7 | 90 | 110 | 430 |
| FW1NBNE02D2CWW-1 | ttcaa | 5 | 62 | 86 | 518 |
| FW1NBNE02DWSD7-1 | ttcagg | 5 | 289 | 318 | 399 |
| contig00040-1 | ttccca | 6 | 255 | 290 | 534 |
| contig00800-1 | ttct | 6 | 33 | 56 | 1006 |
| contig06255-1 | ttctc | 6 | 453 | 482 | 513 |
| contig00066-2 | ttg | 5 | 135 | 149 | 305 |
| contig00707-2 | ttg | 6 | 579 | 596 | 1498 |
| contig01558-2 | ttg | 5 | 455 | 469 | 512 |
| FW1NBNE02EDN8B-2 | ttg | 6 | 362 | 379 | 534 |
| contig00443-1 | ttg | 5 | 405 | 419 | 500 |
| contig00820-1 | ttg | 5 | 1547 | 1561 | 1715 |
| contig07600-1 | ttg | 6 | 40 | 57 | 216 |
| contig10475-1 | ttg | 7 | 402 | 422 | 490 |
| FW1NBNE02C6DMW-1 | ttg | 5 | 261 | 275 | 537 |
| FW1NBNE02DJ86T-1 | ttg | 5 | 435 | 449 | 510 |
| FW1NBNE02EPN7V-1 | ttga | 5 | 480 | 499 | 515 |
| contig01269-1 | ttgt | 5 | 99 | 118 | 316 |
| contig14002-2 | ttta | 5 | 62 | 81 | 128 |
| FW1NBNE02D2PU7-2 | ttta | 5 | 15 | 34 | 57 |
| FW1NBNE02EFKPK-2 | ttta | 7 | 68 | 95 | 124 |
| FW1NBNE02D00H4-2 | ttta | 5 | 66 | 85 | 142 |
| FW1NBNE02DBPIE-2 | ttta | 5 | 17 | 36 | 63 |
| FW1NBNE02DP4YV-2 | ttta | 5 | 56 | 75 | 131 |
| FW1NBNE02EMUSQ-3 | ttta | 5 | 36 | 55 | 181 |
| FW1NBNE02D84SX-3 | ttta | 6 | 74 | 97 | 221 |
| FW1NBNE02EU7U5-3 | ttta | 8 | 78 | 109 | 139 |
| contig13142-1 | ttta | 5 | 7 | 26 | 382 |
| FW1NBNE02EI93G-1 | ttta | 6 | 46 | 69 | 69 |
| FW1NBNE02C5JL2-1 | ttta | 17 | 38 | 105 | 105 |
| FW1NBNE02C6GJQ-1 | ttta | 7 | 66 | 93 | 99 |
| FW1NBNE02C0ZEX-1 | ttta | 6 | 51 | 74 | 74 |
| FW1NBNE02DIYS1-1 | ttta | 5 | 49 | 68 | 68 |
| FW1NBNE02C4HVI-1 | ttta | 6 | 103 | 126 | 126 |
| FW1NBNE02ELPO4-1 | ttta | 6 | 83 | 106 | 106 |
| FW1NBNE02EGJL7-1 | ttta | 5 | 75 | 94 | 94 |
| FW1NBNE02DEBZP-1 | ttta | 5 | 36 | 55 | 55 |
| FW1NBNE02C2QG9-1 | ttta | 7 | 138 | 165 | 165 |
| FW1NBNE02C43TI-1 | ttta | 6 | 76 | 99 | 101 |
| FW1NBNE02DMO77-1 | tttaa | 5 | 2 | 26 | 566 |
| FW1NBNE02DHA7I-3 | tttat | 7 | 1 | 35 | 60 |
| FW1NBNE02EO6G6-2 | tttc | 5 | 52 | 71 | 440 |
| FW1NBNE02EQ6DM-1 | tttc | 8 | 478 | 509 | 509 |
| FW1NBNE02EKJY2-3 | tttcct | 5 | 286 | 315 | 426 |
| contig01599-1 | tttgtt | 5 | 584 | 613 | 735 |
| FW1NBNE02EI93G-2 | tttta | 9 | 5 | 49 | 69 |
| FW1NBNE02C6GJQ-2 | tttta | 7 | 35 | 69 | 99 |
| FW1NBNE02DIYS1-2 | tttta | 7 | 18 | 52 | 68 |
| FW1NBNE02D0JP5-2 | tttta | 8 | 54 | 93 | 118 |
| FW1NBNE02C4HVI-2 | tttta | 7 | 72 | 106 | 126 |
| FW1NBNE02DMURA-2 | tttta | 5 | 29 | 53 | 70 |
| FW1NBNE02ELPO4-2 | tttta | 10 | 23 | 72 | 106 |
| FW1NBNE02EGJL7-2 | tttta | 6 | 49 | 78 | 94 |
| FW1NBNE02DLX6W-2 | tttta | 7 | 3 | 37 | 68 |
| FW1NBNE02C2QG9-2 | tttta | 25 | 17 | 141 | 165 |
| FW1NBNE02C43TI-2 | tttta | 6 | 50 | 79 | 101 |
| FW1NBNE02C0ZEX-2 | tttta | 5 | 30 | 54 | 74 |
| FW1NBNE02DTYHS-3 | tttta | 5 | 25 | 49 | 75 |
| FW1NBNE02DE24P-3 | tttta | 5 | 75 | 99 | 246 |
| FW1NBNE02EU7U5-4 | tttta | 5 | 57 | 81 | 139 |
| FW1NBNE02EMUSQ-5 | tttta | 7 | 5 | 39 | 181 |
| FW1NBNE02D84SX-5 | tttta | 6 | 48 | 77 | 221 |
| FW1NBNE02EGX7C-1 | tttta | 7 | 44 | 78 | 99 |
| FW1NBNE02DA5QE-1 | tttta | 8 | 49 | 88 | 100 |
| FW1NBNE02EIEZD-1 | tttta | 7 | 58 | 92 | 107 |
| FW1NBNE02EBLOL-1 | tttta | 5 | 24 | 48 | 55 |
| FW1NBNE02C59FN-1 | tttta | 6 | 15 | 44 | 55 |
| contig11044-1 | ttttc | 6 | 1 | 30 | 877 |
| contig01604-1 | ttttgt | 6 | 857 | 892 | 1048 |
|  |  |  |  |  |  |
