# Supplementary material for: Analysis of the transcriptome of Panax notoginseng root uncovers putative triterpene saponin-biosynthetic genes and genetic markers
Source: BMC Genomics. 2011 Dec 23;12(Suppl 5):S5. doi: 10.1186/1471-2164-12-S5-S5 (PMC3287501; doi:10.1186/1471-2164-12-S5-S5)
Supplement: Additional file 5 — The discovery of SSR motifs in the putative triterpene saponin-biosynthetic genes. The SSR motifs were detected in the putative triterpene saponin-biosynthetic genes including AACT (acetyl-CoA acetyltransferase), HMGR (HMG-CoA reductase), SS (squalene synthase), SE (squalene epoxidase) and DS (dammarenediol-II synthase). [file 1471-2164-12-S5-S5-S5.doc]

**Additional file 5 The discovery of SSR motifs in the putative triterpene saponin-biosynthetic genes**

| **Gene name** | **Unique sequence** | **SSR motif** | **No. of repeats** | **SSR start**  **(bp)** | **SSR end**  **(bp)** | **Sequence length (bp)** |
| --- | --- | --- | --- | --- | --- | --- |
| *AACT* | contig00313 | tc | 6 | 16 | 27 | 1326 |
| *HMGR* | contig13725 | ct | 7 | 607 | 620 | 787 |
| *SS* | contig04447 | ta | 5 | 1494 | 1503 | 1647 |
| *SE* | contig03811 | cat | 7 | 76 | 96 | 355 |
| *SE* | contig03811 | gag | 6 | 205 | 222 | 355 |
| *SE* | contig13936 | ag | 9 | 41 | 58 | 335 |
| *DS* | contig13785 | ta | 6 | 2555 | 2566 | 2611 |
