# Supplementary material for: Analysis of the transcriptome of Panax notoginseng root uncovers putative triterpene saponin-biosynthetic genes and genetic markers
Source: BMC Genomics. 2011 Dec 23;12(Suppl 5):S5. doi: 10.1186/1471-2164-12-S5-S5 (PMC3287501; doi:10.1186/1471-2164-12-S5-S5)
Supplement: Additional file 7 — Major transcription factor families identified from P. notoginseng using Inter-Pro. The unique sequences from P. notoginseng with similarities to genes encoding transcription factors. [file 1471-2164-12-S5-S5-S7.doc]

## Additional file 7 Major transcription factor families identified from *P*. *notoginseng* using Inter-Pro

| **Transcription factor**  **family descriptions** | **Inter-Pro**  **accession Nos.** | **Frequency** |
| --- | --- | --- |
| AUX/IAA | IPR003311  IPR011525 | 33  61 |
| ARF | IPR010525 | 50 |
| B3 | IPR003340 | 58 |
| Basic helix-loop-helix | IPR001092 | 76 |
| Basic leucine zipper (bZIP) | IPR011700  IPR004827  IPR011616 | 5  42  34 |
| Helix-loop-helix | IPR011598 | 72 |
| Helix-turn-helix | IPR000047  IPR001387 | 10  2 |
| Homeobox | IPR006455  IPR017970  IPR001356  IPR003106 | 1  54  68  15 |
| Homeodomain-like | IPR009057 | 180 |
| Homeodomain-related | IPR012287 | 146 |
| Myb | IPR015495  IPR014778  IPR017877  IPR006447  IPR017930 | 34  115  16  59  98 |
| NAC | IPR002715 | 1 |
| Pathogenesis-related/ERF | IPR001471 | 55 |
| WRKY | IPR003657  IPR017396  IPR018872 | 39  2  7 |
| Zinc finger, C2H2 | IPR015880  IPR013087  IPR007087 | 44  4  44 |
| Zinc finger, CCCH | IPR000571 | 24 |
| Zinc finger, RING | IPR013083  IPR017907  IPR001841 | 13  2  13 |
