# Supplementary material for: Analysis of the transcriptome of Panax notoginseng root uncovers putative triterpene saponin-biosynthetic genes and genetic markers
Source: BMC Genomics. 2011 Dec 23;12(Suppl 5):S5. doi: 10.1186/1471-2164-12-S5-S5 (PMC3287501; doi:10.1186/1471-2164-12-S5-S5)
Supplement: Additional file 10 — The primers used for RACE in this study. The primers used in 5'-RACE for the amplification of unique sequences including Pn01024, Pn02132, Pn03717, and Pn00788. [file 1471-2164-12-S5-S5-S10.doc]

**Additional file 10 The primers used for RACE in this study**

| **Primer name** | **Oligo sequences (5'to3')** |
| --- | --- |
| 5Pn01024out | GCGAGGACTTTAGCATAGATGTGAGG |
| 5Pn01024in | CCCTCGCCATTGGAAATCATAACG |
| 5Pn02132out | CGAGGAATGGGGCTACTGGGTGC |
| 5Pn02132in | GTGGGCTGTTGAGCAAAAGTGACRT |
| 5Pn03717out | GCTTGGTTCTCACGGGTATAGTGTATCC |
| 5Pn03717in | CGCCCAAAGCCGCCATCAAGTCG |
| 5Pn00788out | CGGGGTTTCCCTTAAAGTGAGGTACGG |
| 5Pn00788in | TCATCATCGGAATCGTCGAGGAC |
